# A Flavoprotein Dioxygenase Steers Bacterial Tropone Biosynthesis via Coenzyme A-Ester Oxygenolysis and Ring Epoxidation

**DOI:** 10.1021/jacs.1c04996

**Published:** 2021-07-01

**Authors:** Ying Duan, Marina Toplak, Anwei Hou, Nelson L. Brock, Jeroen S. Dickschat, Robin Teufel

**Affiliations:** † Faculty of Biology, University of Freiburg, Schänzlestrasse 1, 79104 Freiburg, Germany; ‡ Kekulé-Institute of Organic Chemistry and Biochemistry, University of Bonn, Gerhard-Domagk-Strasse 1, 53121 Bonn, Germany; § Institute of Organic Chemistry, TU Braunschweig, Hagenring 30, 38106 Braunschweig, Germany

## Abstract

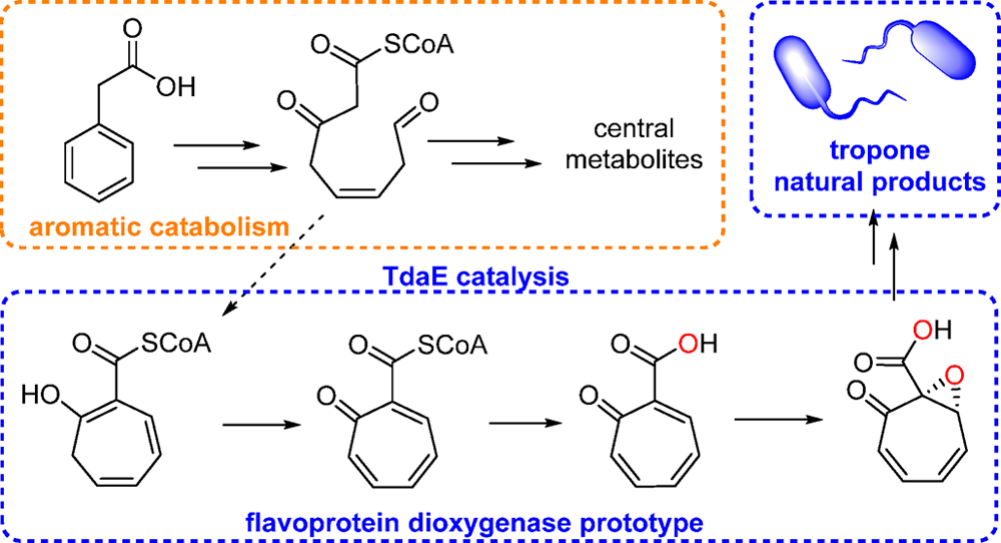

Bacterial tropone natural products such as tropolone, tropodithietic acid, or the roseobacticides play crucial roles in various terrestrial and marine symbiotic interactions as virulence factors, antibiotics, algaecides, or quorum sensing signals. We now show that their poorly understood biosynthesis depends on a shunt product from aerobic CoA-dependent phenylacetic acid catabolism that is salvaged by the dedicated acyl-CoA dehydrogenase-like flavoenzyme TdaE. Further characterization of TdaE revealed an unanticipated complex catalysis, comprising substrate dehydrogenation, noncanonical CoA-ester oxygenolysis, and final ring epoxidation. The enzyme thereby functions as an archetypal flavoprotein dioxygenase that incorporates both oxygen atoms from O_2_ into the substrate, most likely involving flavin-N5-peroxide and flavin-N5-oxide species for consecutive CoA-ester cleavage and epoxidation, respectively. The subsequent spontaneous decarboxylation of the reactive enzyme product yields tropolone, which serves as a key virulence factor in rice panicle blight caused by pathogenic edaphic *Burkholderia plantarii*. Alternatively, the TdaE product is most likely converted to more complex sulfur-containing secondary metabolites such as tropodithietic acid from predominant marine *Rhodobacteraceae* (e.g., *Phaeobacter inhibens*).

## Introduction

**Figure 1:**
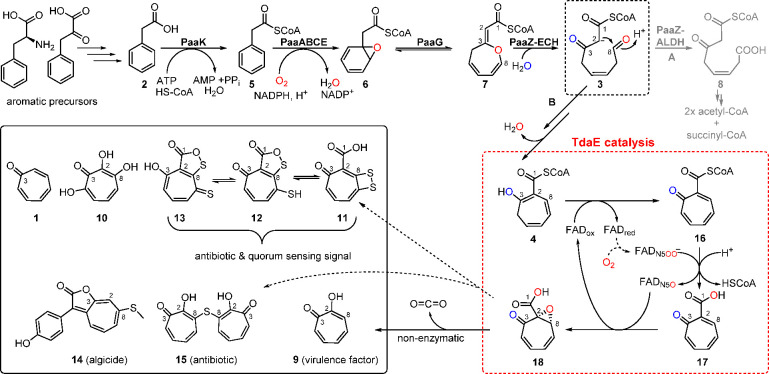
Bacterial **2** catabolic pathway and generation of the proposed universal tropone precursor **4**. Catabolic steps generate reactive **3**, which is oxidized at C8 to the stable carboxylic acid **8**, before final β-oxidation-like steps generate central metabolites (pathway A, gray arrows). Alternatively, **3** spontaneously cyclizes to **4** via an intramolecular Knoevenagel condensation (pathway B, black arrows). TdaE then converts **4** into **18** (red dashed box), which is prone to undergo decarboxylation to natural product **9**. In addition, **18** is likely the precursor for sulfur-containing tropone natural products such as **11**, **14**, and **15**. Oxygen atoms shown in red and blue indicate incorporation from O_2_ and H_2_O, respectively (based on ^18^O-isotope labeling experiments). For details on the flavin-dependent TdaE catalysis, see text and Figure 5. Examples of mature tropone natural products and selected bioactivities are shown in the black box. The carbon numbering for all compounds is according to **3**.

Bacterial natural products that feature a non-benzenoid aromatic tropone core (**1**, Figure 1) are of environmental and pharmaceutical importance and are produced by numerous marine and terrestrial bacteria.^1,2^ Their biosynthesis was previously linked to phenylacetic acid (paa) (**2**) degradation,^3,4^ in which a reactive semialdehyde intermediate (**3**) undergoes an intramolecular condensation reaction to yield the shunt product 2-hydroxycyclohepta-1,4,6-triene-1-formyl-CoA (**4**), which was hypothesized to be the universal tropone precursor based on its structural features (Figure 1).^5^ Compound **2** is typically obtained from the environment and may also arise from the catabolism of other aromatic compounds such as styrene, ethylbenzene, or phenylalanine.^3,6^ In addition, **2** can be generated from the anabolic shikimate pathway product phenylpyruvic acid, which is likely a common strategy for tropone natural product forming bacteria.^5,7^ The formation of **4** from **2** typically requires four enzymes. First, **2** is activated by the phenylacetate:CoA ligase PaaK, which generates phenylacetyl-CoA (**5**).^8−10^ Alternatively, **5** is directly produced from phenylpyruvic acid, as previously shown for *Phaeobacter inhibens*.^6^ Compound **5** is then epoxidized and dearomatized to 1,2-epoxyphenylacetyl-CoA (**6**) by the di-iron-dependent multicomponent monooxygenase PaaABCE,^3,11,12^ before the isomerase PaaG converts **6** into (*Z*)-2-(oxepin-2(3*H*)-ylidene)-acetyl-CoA (“oxepin-CoA”, **7**).^3,13,14^ The α,β-unsaturated **7** is further processed by a ring-cleaving enoyl-CoA hydratase (ECH), either as a standalone enzyme or as part of the bifunctional fusion protein PaaZ, which typically comprises a C-terminal ECH and an N-terminal aldehyde dehydrogenase (ALDH) domain.^5^ The formed semialdehyde intermediate **3** is highly reactive and could not be observed or captured by derivatization so far.^5^ Either this aldehyde group is immediately oxidized to the more stable carboxylate (**8**) by the PaaZ-ALDH domain or a separate ALDH along the downstream steps of the *paa* catabolon that is followed by β-oxidation-like steps,^3^ or a rapid spontaneous intramolecular Knoevenagel condensation to shunt product **4** occurs (Figure 1).

Some bacteria appear to have developed mechanisms to boost **4** formation, as exemplified by *P. inhibens*, which encodes a PaaZ homologue with an ALDH domain that lacks the catalytic residues for aldehyde oxidation and thereby likely drives its accumulation.^15^ Compound **4** may then be converted, for example, into tropolone (**9**) and hydroxytropolones (e.g., **10**) by *Burkholderia* spp. (including plant pathogens such as *B. plantarii*),^16−18^*Pseudomonas donghuensis*,^19,20^ and *Streptomyces* spp.^21^ In addition, **4** is most likely the precursor for more complex sulfur-containing derivatives, i.e., tropodithietic acid (**11**) (and its tautomers troposulfenin (**12**) and thiotropocine (**13**))^4,15,22^ and the roseobacticides A–G^23^ (e.g., roseobacticide A; **14**) from predominant marine *Rhodobacteraceae* (*Roseobacter* spp., *Phaeobacter* spp., or *Pseudovibrio* spp. among others), as well as a sulfur-bridged tropolone dimer (ditropolonyl sulfide) (**15**) from the human pathogen *Burkholderia cenocepacia*,^24^ which can infect cystic fibrosis patients. Many of these compounds are critical for symbiotic interactions; for example, **11**(25,26) is produced by bacteria that often live associated with marine invertebrates (sponges, tunicates, soft and stony corals, tube worms, shellfish, among others) and algae^1,26^ and likely serves as an antibiotic that protects the host organisms against pathogens such as *Vibrio* spp.. Interestingly, **11** was also shown to act as a quorum sensing signal that triggers major changes in bacterial gene expression.^22^ Similarly, tropolones play important roles in symbiotic interactions, most notably the antagonism of *Burkholderia* spp. such as *B. plantarii* that cause bacterial panicle blight in rice plant seedlings and thus pose a threat to global rice production.^16,27^ It was shown that **9** is the key virulence factor of *B. plantarii* and likely deprives the plants of essential iron via chelation.^28,29^

As of yet, the enzymatic step that links bacterial tropone biosynthesis with **2** catabolism has not been verified, and downstream biosynthesis consequently remains poorly understood.^1,30^ Biosynthetic gene clusters (BGCs) were previously reported for **11** (also required for the roseobacticides)^4,31,32^ from *Phaeobacter* spp. and for **10** from *Streptomyces* spp.,^21^ but direct evidence for the roles of the encoded enzymes is lacking.^1^ In contrast, BGCs for the formation of toxic **9** and the antibiotic **15** from *B. plantarii and B. cenocepacia* have not been reported to date.

We now show that **4** indeed serves as a central precursor for structurally distinct bacterial tropone natural products and as a substrate for the key flavoenzyme TdaE, which is encoded by the previously reported **11**-BGCs of marine *Rhodobacteraceae* and by the newly identified putative BGCs for the generation of **9** and **15** in *Burkholderia* spp. Our studies include the detailed analysis of the reactions catalyzed by heterologously produced TdaE homologues and the probing of the enzyme mechanism using LC-HRMS and ^18^O isotope labeling experiments among other techniques, which reveal a surprising prototypal dioxygenase functionality. The rapid spontaneous decomposition of the unstable TdaE product strongly hampered its structure elucidation, which could only be achieved through the use of ^13^C-labeled precursors and by a combination of chemical derivatization and comparison to an enantioselectively synthesized reference compound. Ultimately, the reactive TdaE product either spontaneously forms **9** or is likely further transformed into **11** and other sulfur-containing tropone natural products.

## Results

### *Burkholderia* spp. Harbor *tdaE* Homologues for the Production of Tropolone and Ditropolonyl Sulfide

**Figure 2:**
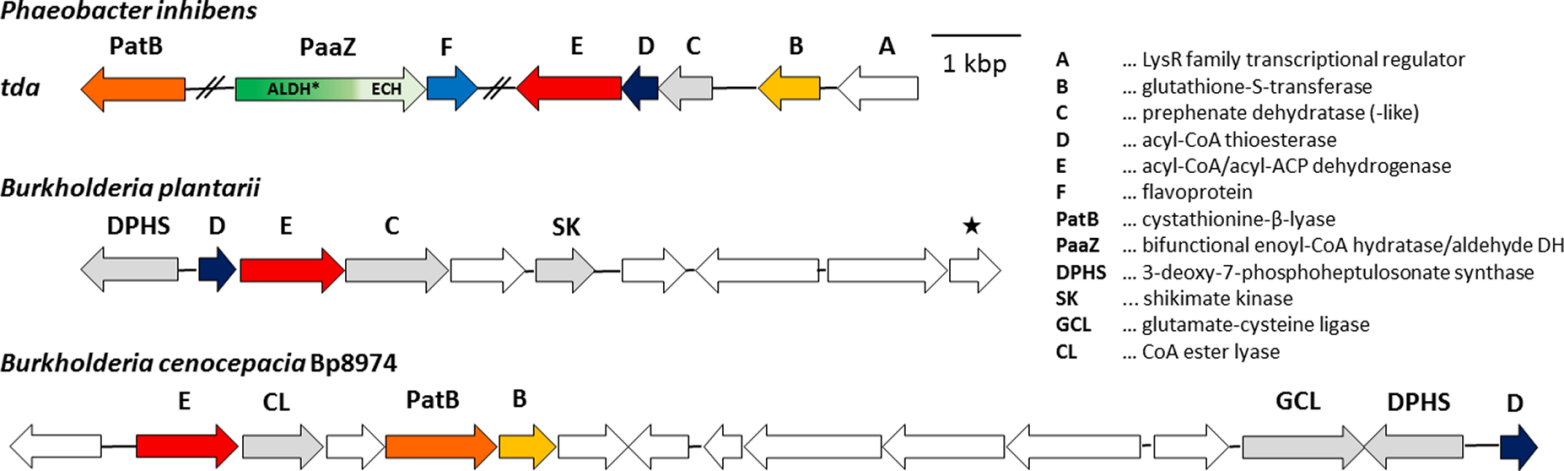
Proposed biosynthetic gene clusters for bacterial tropone natural products with the encoded proteins shown on top (*paa* catabolic genes are located elsewhere in the genome, cf. Figure S1). Top: Verified *tda* gene cluster from *P*. *inhibens* for production of **11** that contains a second copy of *paaZ* encoding an enzyme with a dysfunctional ALDH domain. The newly identified putative gene clusters for **9** and **15** formation from *B*. *plantarii* and *B*. *cenocepacia* are shown below. Genes predicted to encode enzymes involved in supplying pathway precursors are shown in gray with the exception of *patB* (orange) and *tdaB* (yellow), which presumably encode enzymes for formation and incorporation of the sulfur precursor, respectively. Other genes encoding proposed enzymes involved in downstream processing of **4** toward mature tropone natural products are also color coded individually (*tdaE* is shown in red). Genes encoding transcriptional regulators, putative transporters, and proteins of unknown function are shown in white. The gene encoding a putative decarboxylase of *B. plantarii* that was investigated in this work is marked with an asterisk. See Tables S1–S3 for details and accession numbers of the predicted proteins.

To identify putative BGCs of tropone natural products in pathogenic *Burkholderia* species, protein BLAST searches were conducted using proteins as queries that were previously associated with tropone biosynthesis in addition to enzymes involved in producing aromatic precursors *de novo* via the shikimate pathway.^1^ The search was focused on *B. plantarii* and *B. cenocepacia* strains, reported to produce **9** and **15**, respectively. Initially, a predicted thioesterase and two flavoprotein monooxygenases (FPMOs) from the recently reported *Streptomyces* spp. were used as queries. Both these enzymes are essential for the production of hydroxytropolones such as **10**,^21^ presumably by mediating CoA-thioester cleavage as well as ring oxidation and hydroxylation.^1,21^ However, no genomic regions encoding such enzymes were found; instead, homologues of genes from **11** biosynthesis of *P. inhibens* were identified in both *B. plantarii* and *B. cenocepacia* Bp8974 as part of putative BGCs. These genes encoded enzymes of the shikimate pathway as well as homologues of the predicted flavoenzyme TdaE from **11** biosynthesis that was suggested to be involved in the downstream processing of **4** (Figure 2).^1,15^ TdaE has low similarity to flavin-dependent acyl-CoA dehydrogenases (ACADs) and was previously proposed to catalyze the two-electron oxidation of the dihydrotropone moiety of **4**.^15^ In addition, consistent with the structure of **15**, the BGC of *B. cenocepacia* encoded homologues of putative sulfur precursor-synthesizing (PatB) and -incorporating (TdaB) enzymes that were previously found in the BGCs of **11** producers (Figure 2).^33^

### TdaE Catalysis Involves Initial Substrate Dehydrogenation

**Figure 3:**
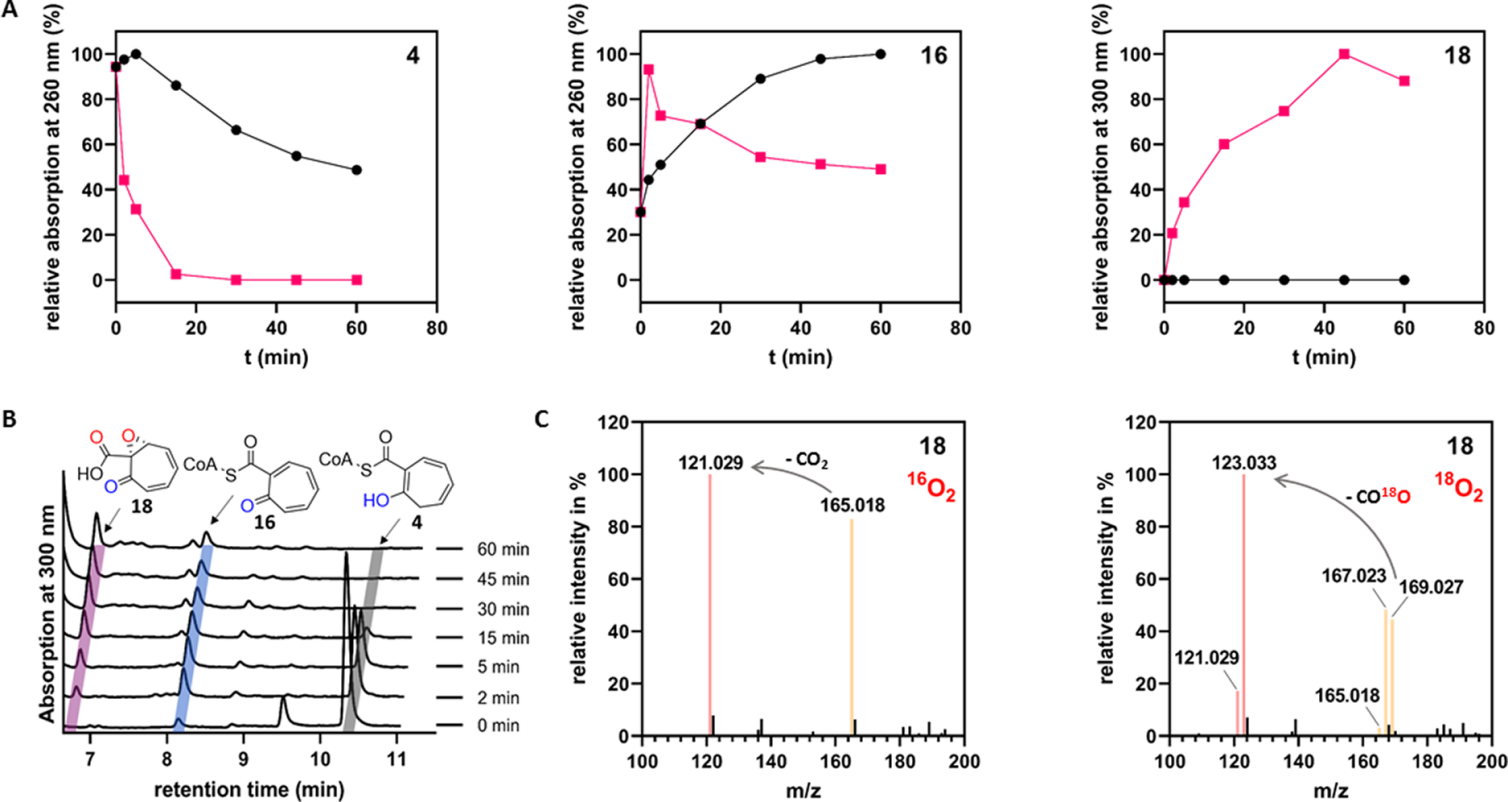
Time course of **4** conversion into **18** by TdaE*Pi* and analysis of ^18^O incorporation into compound **18** by LC-HRMS. (A) Time course for the conversion of **4** via **16** into **18**, as determined by RP-HPLC analysis. The pink curves correspond to the assays containing TdaE*Pi*, demonstrating that after a rapid conversion of **4** into **16**, compound **18** is produced. The control reaction (black curves) only shows the spontaneous oxidation of **4** to **16** without formation of **18**. (B) HPLC chromatograms (at 300 nm) corresponding to the time course graphs shown in A, with the substrate **4**, the reaction intermediate **16**, and the final product **18** indicated by gray, blue, and purple lines, respectively. The peak with a retention time of 9.5 min (at *t*(0 min), prior to the addition of TdaE*Pi*) forms spontaneously from **4** and likely represents a tautomer that is converted into **16** by TdaE. (C) MS fragmentation pattern of compound **18** (negative ion mode) generated in TdaE assays in the presence of either ^16^O_2_ (left) or ^18^O_2_ (right), in which the in-source fragmentation of **18** to **9** by decarboxylation can be observed.

To investigate the role of TdaE in the biosynthesis of sulfur-containing tropone derivatives and of tropolone, the TdaE homologues encoded by the BGCs of *P. inhibens* (TdaE^*Pi*^; NCBI accession ID: WP_014881725.1) and *B. plantarii* (TdaE^*Bp*^; NCBI accession ID: WP_042624079.1) were heterologously produced and purified. Both enzymes could eventually be obtained in soluble form (Figures S2–S4) with an N-terminal maltose-binding protein (MBP) for TdaE^*Pi*^ and an N-terminal GB1 (subunit B of protein G) as well as a C-terminal polyhistidine tag for TdaE^*Bp*^ (both tags were required to obtain soluble and stable enzyme). After protein purification, TdaE^*Pi*^ was obtained almost colorless, whereas TdaE^*Bp*^ was weakly yellow due to a loosely bound FAD cofactor (Figures S5, S6) (based on c_280_/c_450_ stoichiometry protein:FAD ca. 5:1). To investigate a possible biosynthetic role of TdaE, *in vitro* enzyme assays were established in which chemically synthesized **5** was used as a substrate for heterologously produced PaaABCE, PaaG, and PaaZ-E256Q (a PaaZ variant with inactive ALDH domain that reroutes the *paa* catabolic pathway to the formation of **4**).^5^ Addition of either TdaE^*Pi*^ or TdaE^*Bp*^ further converted **4** into a new compound that also formed spontaneously at much lower rates and was retained in the aqueous phase after organic extraction with ethyl acetate (EtOAc). LC-HRMS analysis supported the envisaged two-electron oxidation reaction of **4** to **16** (MH^+^*m/z* 900.145) (Figure S7). To verify the proposed structure of **16**, the CoA ester was hydrolyzed with heterologously produced thioesterase PaaY, which normally salvages trapped CoA from the inhibitory shunt product **4** in **2**-degrading bacteria.^11^ Following the extraction with EtOAc, the hydrolysis product was identified as tropone-2-carboxylate (**17**) (MH^–^*m*/*z* 149.024) as it exhibited the same retention time as well as identical UV–vis and HRMS spectra compared to a chemically synthesized standard^25^ (Figure S8). Notably, **16** also formed spontaneously from **4** in TdaE-free control reactions, albeit significantly slower (Figure 3A,B). These findings confirmed the TdaE dehydrogenase functionality, consistent with the homology to ACADs. However, following the initial accumulation of **16** in the TdaE assays, a rapid decrease of this compound was observed, suggesting that **16** may only represent an intermediate in the TdaE-catalyzed reaction (Figure 3A,B). To investigate this, TdaE assays were scrutinized over time, revealing the generation of a distinct final product that could not be observed in control assays (Figure 3A,B; Figures S9, S10).

### Product Characterization Reveals Subsequent TdaE-Mediated CoA-Ester Cleavage and Ring Epoxidation

The comparably low polarity of the newly formed TdaE product suggested the loss of the CoA moiety, in line with the results from LC-HRMS analysis (MH^−^*m*/*z* 165.018) that pointed to the incorporation of another oxygen atom (calculated for C_8_H_5_O_4_^–^*m*/*z* 165.019, Figure 3C, left panel). In contrast, heterologously produced TdaD (Figures S11, S12), a thioesterase-like enzyme previously speculated to be responsible for CoA-ester cleavage in **11** biosynthesis and also encoded in the *Burkholderia* spp. gene clusters (Figure 2), processed neither **4** nor **16**, which together with the observation that TdaE itself eliminates CoA implies a different function for TdaD. Both enzymes TdaE^*Pi*^ and TdaE^*Bp*^ catalyzed the same reaction, suggesting that the conversion of **4** by TdaE is relevant for the formation of tropolone as well as of sulfur-containing tropone natural products (see Figure S13 and *vide infra* for a gene deletion experiment). To elucidate the structure of the final TdaE product **18**, large-scale enzymatic assays were conducted; however, the compound proved unstable and slowly decomposed in the enzyme assays (Figure S14) and more rapidly during NMR sample preparation into a volatile compound that was easily lost in concentration steps. Several trials to isolate the TdaE product in sufficient amounts failed, precluding its structure elucidation by standard NMR-based methods (only partial 1D and 2D NMR spectra showing signals for five hydrogens, but only two signals for olefinic/aromatic CH groups could be obtained; Figures S15 and S16). Therefore, an isotopic labeling strategy was employed to enhance the missing ^13^C NMR signals for elucidation of the structure of the TdaE product. For this purpose, (^13^C_8_)-**2** was chemically synthesized according to Scheme S1 in the Materials and Methods (for NMR spectra of intermediates and (^13^C_8_)-**2** cf. Figures S17–S25) and enzymatically converted into (^13^C_8_)-**5** by PaaK to serve as a substrate for the enzyme assay with PaaABCE, PaaG, PaaZ-E256Q, and TdaE. The product was enriched by RP-HPLC and analyzed by 1D and 2D ^13^C NMR spectroscopic methods. During measurement in CD_3_CN, however, the labeled compound once more gradually degraded under accumulation of a breakdown product, showing cross-peaks in the ^13^C,^13^C-COSY NMR (Figure S26) only between four sp^2^ carbons (one quaternary and three CH groups). This spin system was reflected by ^13^C–^13^C couplings in the ^13^C NMR spectrum (Figure S15) in line with the pseudosymmetrical (*C*_2*v*_) structure of (^13^C_7_)-**9**. In this compound the two halves can interconvert by fast keto–enol tautomerism, making them identical on the NMR time scale. The identity of (^13^C_7_)-**9** was confirmed by spiking the NMR sample with commercially available unlabeled **9** (Figure S27).

For a full understanding of the formation of **9**, the identification of its unstable precursor **18** was required. Enzymatic conversion of (^13^C_8_)-**2** and commercially available (1,2-^13^C_2_)-**2** with optimization of the workup procedure and immediate NMR measurements of the freshly obtained samples allowed the identification of the final TdaE products by ^13^C NMR, ^13^C,^13^C-COSY NMR, and HSQC (Figures S28–S31 and Table S4) through the strong enhancement of all ^13^C-based NMR signals as (^13^C_8_)- and (2-^13^C)-2,3-epoxytropone-2-(^13^C)-carboxylate (**18**). Hence, these results suggest that following the oxidation of substrate **16** by dehydrogenation, TdaE surprisingly cleaves off the CoA moiety and epoxidizes the tropone ring to afford the final product **18** (possibly via intermediate **17**), which decomposes to **9** by facile decarboxylative epoxide ring opening during sample preparation and in the course of NMR and LCMS measurements (Figures S27 and S32 and Figure 3C). Notably, in contrast to previously reported similar epoxidation reactions,^34^**18** formation did not involve a 1,2-rearrangement of the carboxylate group based on the ^1^*J*_C,C_ doublet couplings observed for (^13^C_2_)-**18** (Figure S29).

To determine the absolute configuration of **18**, an enantioselective synthesis of methyl (2*S*,3*S*)-2,3-epoxytropone-2-carboxylate (**24**) was conducted starting from cycloheptan-1,3-dione (**19**) by condensation with two units of formaldehyde to **20** and reduction with diisobutylaluminum hydride (DIBAL-H) to the allyl alcohol **21**. Sharpless epoxidation with l-(+)-diisopropyl tartrate to (2*S*,3*S*)-**22** (94% *ee*), oxidation to the carboxylic acid **23**, and methylation resulted in (2*S*,3*S*)-**24** (Figure 4A, for NMR spectra of synthetic intermediates and **24**, cf. Figures S33–S44). This synthetic compound was compared to the TdaE product **18**, which was converted into **24** by microderivatization (catalytic hydrogenation and methylation; Figure 4B). Analysis by GC/MS on a chiral stationary phase in comparison to synthetic (*rac*)-**24** (prepared according to Scheme S3 in the Materials and Methods, relevant NMR spectra are shown in Figures S45–S53) and (2*S*,3*S*)-**24** revealed that **24** obtained from the enzyme product **18** is the opposite enantiomer of synthetic (2*S*,3*S*)-**24** (Figures S54) and thus has (2*R*,3*R*) configuration; that is, the TdaE enzyme product is (2*R*,3*R*)-**18**.

**Figure 4:**
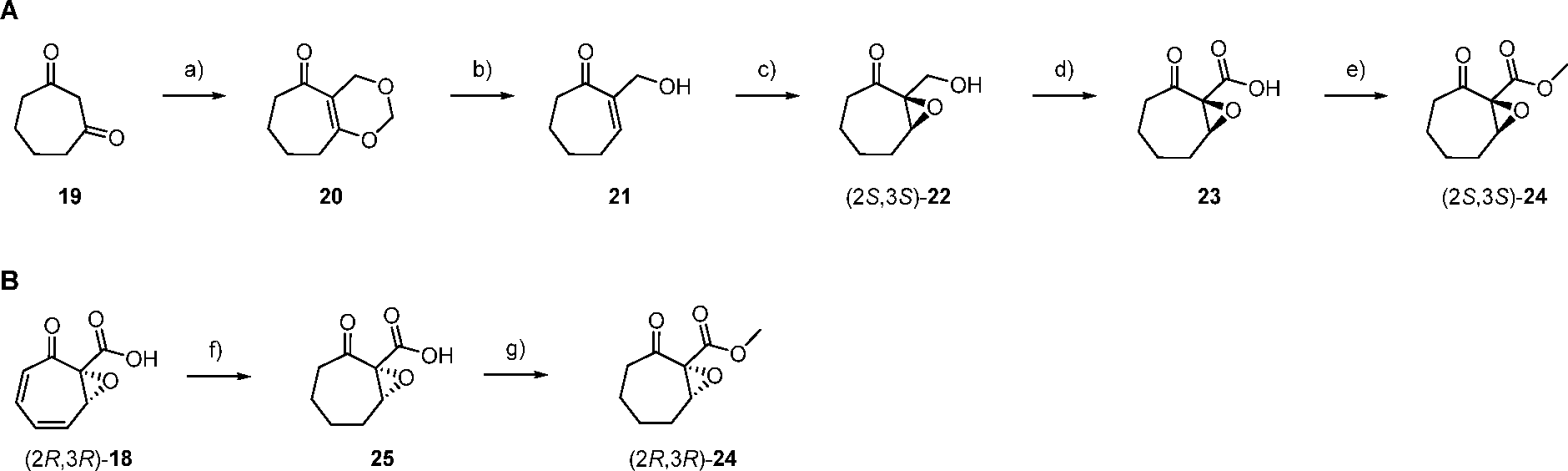
Determination of the absolute configuration of enzymatically generated **18**. (A) Enantioselective synthesis of (2*S*,3*S*)-**24**. Reaction conditions: (a) paraformaldehyde, BF_3_·OEt_2_, CH_2_Cl_2_, room temperature, 3 h, 35%; (b) DIBAL-H, THF, −78 °C, 2 h, 78%; (c) l-(+)-diisopropyl tartrate, Ti(OiPr)_4_, 4 Å molecular sieves, *t*-BuOOH, CH_2_Cl_2_, −17 °C, 20 h, 47%; (d) Jones reagent, acetone, room temperature, 3 h; (e) trimethylsilyldiazomethane, Et_2_O, 0 °C, 40 min, 24% over two steps. (B) Microderivatization of **18**: (f) Pd/C, MeOH, room temperature, 30 min; (g) trimethylsilyldiazomethane, benzene, room temperature, 30 min.

### TdaE Functions as an Archetypal Internal Flavoprotein Dioxygenase

**Figure 5:**
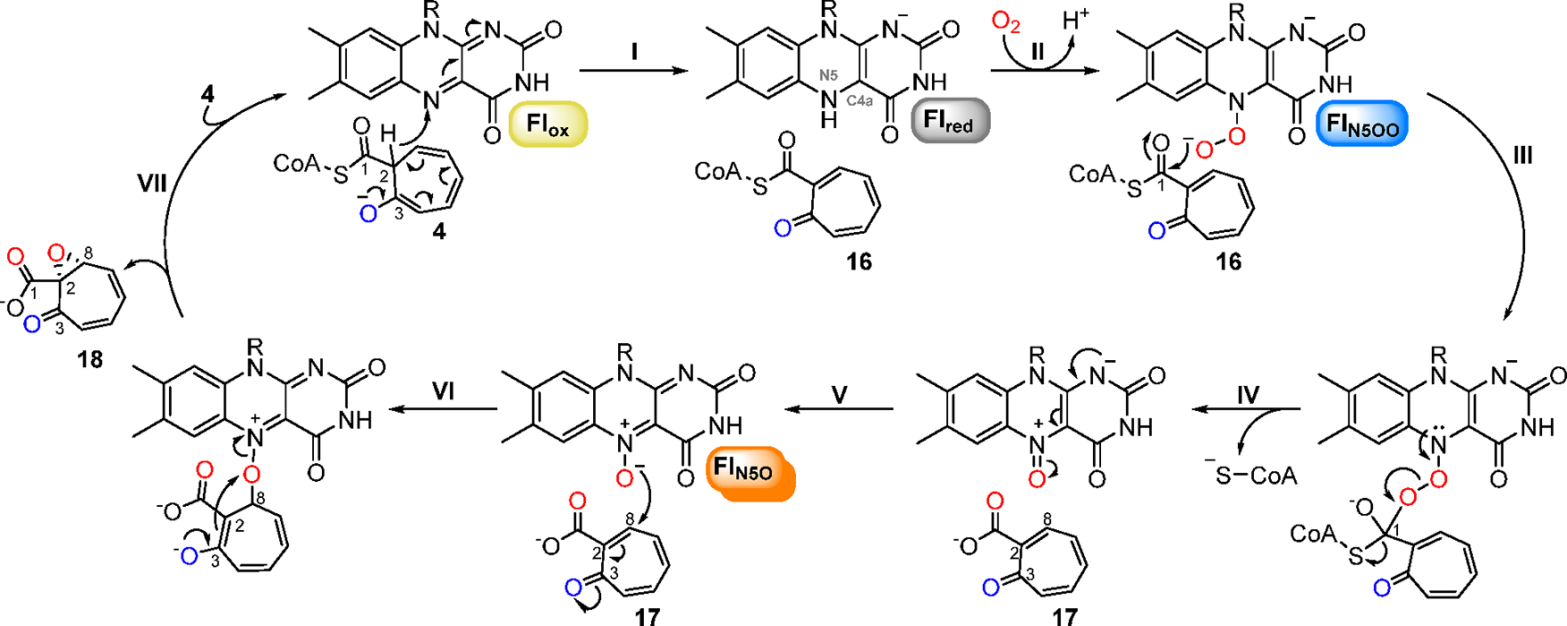
Mechanistic scheme for TdaE catalysis. Note that a tautomer of **4** is shown as substrate for TdaE. The carbon numbering of all compounds is according to Figure 1. See text for details on the individual steps. R = ribityl-ADP.

To further study the formation of **18**, ^18^O-isotope labeling experiments with H_2_^18^O and ^18^O_2_ were conducted. Unexpectedly, no ^18^O incorporation from H_2_^18^O into the carboxy group of **18** was observed by LCMS in the TdaE assays, inconsistent with conventional hydrolytic CoA-ester cleavage (Figures S55 and S56). Instead, two ^18^O atoms were incorporated into **18** from ^18^O_2_ (Figure 3C), implying that both added oxygens result from enzyme-mediated oxygenation. This was further confirmed by the observed in-source fragmentation of **18** via decarboxylation to **9** during the LCMS measurements that demonstrated the loss of one of the two ^18^O_2_-derived ^18^O labels (Figure 3C). Together, these data suggest that TdaE functions as a rare internal oxygenase by using its own substrate **4** as electron donor for flavin reduction and O_2_ activation (rather than external oxygenases, which require NAD(P)H as “co-substrate”).^35^ Consistent with that, TdaE-Fl_ox_ did not react with NAD(P)H based on UV–vis spectroscopic analysis (Figure S57) and remained active in enzyme assays lacking NADPH (normally required by PaaABCE) that were started from purified **7** rather than **5** (Figure 3C, left panel; Figure S55). Notably, TdaE-Fl_ox_ reduction by **4** would likely require the C2-protonated tautomer, which may be formed in the active site of TdaE. In normal TdaE assays, however, such a tautomerization could not be observed, most likely because the subsequent steps proceeded too fast. To further investigate this, TdaE with low FAD cofactor loading was used (to slow down dehydrogenation), which indeed led to the rapid conversion of **4** into a new compound that likely represents the proposed tautomer (as shown in Figure 5). This compound also formed spontaneously in much lower amounts in control samples lacking TdaE (Figure 3, 9.5 min peak in the *t*(0 min) sample and Figure S58).

Subsequent to **16** formation, TdaE-Fl_red_ reacts with O_2_ and incorporates both activated oxygen atoms into the substrate, which is highly unusual for flavoproteins that normally exclusively function as monooxygenases.^35^ Moreover, the chemical properties of the well-studied classical flavin-C4a-(hydro)peroxide (Fl_C4aOO(H)_) oxygenating species^35−37^ seem inconsistent with the observed reactions. First, the Fl_C4aOO(H)_ species could only be formed once per catalytic cycle with two available electrons for O_2_ activation, and despite many known functions of Fl_C4aOO(H)_-dependent FPMOs,^35,38^ the incorporation of both oxygens into a substrate has not been reported.^35,36^ Second, the observed oxygenation chemistry appears incompatible with the chemical properties of the Fl_C4aOO(H)_,^35−37^ specifically the required redox neutral transfer of an [OH]^−^ for CoA-ester oxygenolysis. Typically, such a reaction is achieved by water-activating hydrolases such as thioesterases. Recently, however, novel paradigms for FPMOs were demonstrated that involved N5-oxygenated flavin cofactors in the form of the flavin-N5-peroxide (Fl_N5OO_) and the flavin-N5-oxide (Fl_N5O_) employed for the redox-neutral oxygenolytic cleavage of carbon–heteroatom bonds (e.g., the amide-bond cleaving pyrimidine oxygenase RutA)^35,39^ and for polyketide hydroxylation (EncM from enterocin biosynthesis),^40,41^ respectively. TdaE may thus constitute the first member of a novel class of flavoprotein dioxygenases, which combines Fl_N5OO_ and Fl_N5O_ catalysis by first oxygenolytically cleaving the CoA ester with the Fl_N5OO_ species, before epoxidizing the tropone ring with the help of the formed Fl_N5O_, thereby giving rise to **18** and Fl_ox_ (Figures 1 and 5).

To gain a better understanding of the oxygenation mechanism, it was further investigated whether TdaE-Fl_ox_ can convert its intermediate **16** in the absence of its native electron donor **4**. For that, **16** was isolated and then separately incubated with TdaE. As anticipated, **16** was not processed by TdaE in the presence of NAD(P)H, whereas formation of **18** was observed when Fl_red_ was generated by a separate flavin reductase, once more underling that CoA-ester cleavage proceeds oxygenolytically rather than by classical hydrolysis. Importantly, in contrast to previous assays, **17** accumulated in the presence of the flavin reductase aside from **18**, which suggests the partial reduction of Fl_N5O_ to Fl_ox_ that prevented the second oxygen transfer (Figure S59). This observation also provides evidence that thioester oxygenolysis precedes ring epoxidation, fully in line with the mechanistic proposal. Crucially, scrupulous LC-HRMS analysis indicated small amounts of transient Fl_N5O_ species in the TdaE assays (quenched shortly after the reaction start before complete conversion of **4** into **18**) that could be identified based on characteristic mass spectral data and retention time (Figure S60). To test whether Fl_N5O_ catalysis proceeds via radical intermediates, radical scavengers (ascorbate, 5,5-dimethyl-1-pyrroline-*N*-oxide) were added to the enzyme assays. However, 5,5-dimethyl-1-pyrroline-*N*-oxide hardly affected catalysis, and only mild effects were observed for ascorbate (≈30% lowered product formation), pointing toward a nonradical epoxidation mechanism.

Taken together, TdaE catalysis may first involve the deprotonation of the C3-hydroxyl group of **4** under concomitant transfer of a C2-hydride to the N5 of FAD, similar to oxidations catalyzed by classical ACADs (step I, Figure 5). Then, the formed Fl_red_ reacts with O_2_ to the Fl_N5OO_ (step II) most likely via transient flavin semiquinone (Fl_SQ_) and superoxide radicals.^42^ The CoA ester of the produced **16** is subsequently attacked by the nucleophilic Fl_N5OO_ to form a tetrahedral covalent adduct (step III), followed by CoA-ester oxygenolysis via heterolytic cleavage of the peroxy species (step IV). The resulting Fl_N5O_ should then be properly positioned for a Michael addition at C8 of **17**, which ultimately leads to Fl_ox_ elimination via C2,C8-epoxide formation (steps V and VI) and the generation of **18** (step VII).

### TdaE Is Distinct from Classical ACAD-like Flavoenzymes

To analyze the relationship of TdaE with characterized ACADs and group D FPMOs with ACAD fold, a multiple sequence alignment and a homology model of TdaE^*Pi*^ was generated. Strikingly, while TdaE operates as an oxygenase, the predicted overall structure and active-site architecture more resemble classical ACADs. Moreover, the sequence alignment revealed highly conserved amino acids, including active site residues, in all predicted functional homologues of TdaE from both *Burkholderiacea* (β-Proteobacteria) and *Roseobacteracea* (α-Proteobacteria). These residues were lacking in both classical acyl-CoA dehydrogenases and group D FPMOs (Figures S61 and S62), consistent with the unusual TdaE functionality.

### TdaE Connects the Phenylacetate Catabolon with Tropone Biosynthesis *inVivo*

To further verify **18** as a key intermediate in the biosynthesis of tropone natural products, cell-free lysates from *B. plantarii* and *P. inhibens* were prepared from liquid cultures in the production phases of **9** and **11**, respectively. The lysate from *P. inhibens* converted enzymatically produced (^13^C_8_)-**4** into (^13^C_8_)-**18** (Figure S63), pointing to the presence of TdaE during **11**-production. This result was confirmed by RT-qPCR, revealing a strong upregulation of *tdaE*^*Pi*^ expression in the main phase of **11** production (Figure S64, top). The requirement of TdaE for the generation of **11** was further shown by construction of a *P. inhibens* Δ*tdaE* mutant in which the *tdaE* gene was replaced with a kanamycin resistance cassette, leading to an abolished production of **11** under accumulation of **1** (Figure S65). Similar to that, the production of **1** was previously reported in a **2**-degrading *Azoarcus evansii* mutant strain that lacked a functional **3**-oxidizing ALDH^30^ and therefore most likely accumulated **4** analogous to *P. inhibens* Δ*tdaE*. These observations suggest that unprocessed **4** degrades to **1** within these mutant strains by CoA-ester hydrolysis, decarboxylation, and oxidation. Comparable to *P. inhibens*, (^13^C_8_)-**4** was converted into (^13^C_8_)-**18** by the *B. plantarii* lysate, and strong upregulation of *tdaE*^*Bp*^ expression was observed in the **9**-production phase (Figure S64, bottom). In addition, (^13^C_8_)-**18** was more rapidly transformed into (^13^C_8_)-**9** by the cell lysate from *B. plantarii* in comparison to that from *P. inhibens* or to the spontaneous decomposition of **18** into **9** observed in the *in vitro* TdaE assays (Figure S66). In the BGC of *B. plantarii*, a gene encoding a putative decarboxylase (NCBI accession ID: WP_052498255.1) was found in the vicinity of *tdaE*^*Bp*^ (Figure 2). To test if the corresponding enzyme boosts **9** formation, it was heterologously produced with an N-terminal MBP-tag and purified (Figure S67). However, the soluble decarboxylase-like enzyme had no effect on the formation rate of key virulence factor **9** in the *in vitro* enzyme assays, thus suggesting that decarboxylation is possibly accelerated by another enzyme (Figure S68). Overall, these data support the proposed role of TdaE homologues as bacterial key enzymes for formation of structurally diverse tropone-containing natural products by sequestering shunt product **4** from the *paa* catabolon.

## Discussion

In this work we provide evidence for a biosynthetic route in which a dead-end product from aromatic catabolism is sequestered by the bacterial flavoenzyme TdaE as precursor for bioactive tropone natural products and thereby illustrate an unusual intertwining of primary and secondary metabolism. Our genomic analyses revealed previously unknown BGCs most likely required for **9** and **15** biosynthesis in *B. plantarii* and *B. cenocepacia*, respectively, which encode homologues of TdaE originally identified in the **11** BGCs of marine *Rhodobacteraceae*(e.g., *P. inhibens*). The comparison and investigation of both TdaE^*Bp*^ and TdaE^*Pi*^ showed that they catalyze the virtual identical conversion of **4** into **18** via the intermediates **16** and **17**. TdaE homologues therefore appear to play pivotal roles for the biosynthesis of an abundance of structurally distinct tropone natural products including **9** as well as more complex sulfur-containing **11**, **14** (and other roseobacticides B–K), and **15**. This suggests that the final TdaE product **18** represents an advanced intermediate for formation of these compounds, which is supported by the observed conversion of **4** into **18** by both cell-free lysates of **11**-producing *P. inhibens* and **9**-producing *B. plantarii*. Remarkably, following the efficient TdaE-catalyzed conversion of the catabolic shunt product **4** into **18**, compound **9** is spontaneously formed via rearomatization and decarboxylation, which is facilitated by the epoxide moiety of **18**. Overall, this suggests that TdaE is key to the formation of the critical virulence factor **9** in *B. plantarii* and thus a driving factor for rice seedling blight. Future studies may aim at the development of TdaE inhibitors to shut down **9** formation in such pathogens. On the other hand, the downstream biosynthetic steps to sulfur-containing tropones are more elaborate, requiring sulfur precursors presumably formed and incorporated into the tropone ring by PatB and TdaB homologues, respectively (Figure 2).^15,33^ Notably, **18** seems predisposed to react with nucleophiles, and sulfur incorporation may thus proceed via 1,6-conjugate addition at C7 and epoxide ring opening at C8 en route to **11**. The biosynthesis of **15** and the roseobacticides involves further steps such as the elimination of the carboxyl side chain. Given the highly promising pharmaceutical features and biotechnological potential of these compounds that are also critical for numerous marine and terrestrial symbiotic interactions,^1^ TdaE could therefore be exploited for the future bioengineering of tropone natural product producer strains.

The investigation of TdaE catalysis furthermore exposed an unanticipated series of reactions. First, TdaE relies on its substrate **4** as electron donor (rather than NAD(P)H) for O_2_ activation and covalent flavin-oxygen adduct formation. Then, TdaE incorporates both O_2_-derived oxygen atoms into the substrate most likely via transient Fl_N5OO_ and Fl_N5O_ species, thereby breaking the CoA thioester bond and epoxidizing the tropone ring. This novel paradigm for natural product tailoring via N5-oxygenated flavins effectuating dioxygenation is supported by the observed formation of the Fl_N5O_ species during TdaE catalysis. Thus far, flavoproteins were exclusively reported as monooxygenases that typically rely on transiently produced Fl_C4aOO_ species to process a substantial variety of different substrates.^36−38,43−46^ This is exemplified by the group D FPMO *p*-hydroxyphenylacetate 3-hydroxylase,^38,47^ which shares the ACAD protein fold with TdaE and utilizes the canonical Fl_C4aOO_ species for aromatic hydroxylation.^35,37^ This monooxygenase dogma hitherto also held true for flavoenzymes relying on N5-oxygenated flavin cofactors for catalysis,^35^ i.e., Fl_N5OO_-dependent RutA-like group C FPMOs,^39^ which generate the Fl_N5O_ as a byproduct (that is not used for a second oxygen transfer),^39,48^ and the putative group I FPMO EncM,^40−42^ which transiently forms the Fl_N5OO_ as a precursor for its stable Fl_N5O_ oxygenating species.^41,42^ TdaE accordingly represents the first known flavoprotein dioxygenase, and the discovery of Fl_N5O(O)_ species in a third structural type of flavoproteins furthermore underlines the notion that the microenvironment around the flavin cofactor rather than the overall fold controls O_2_ reactivity and thereby enzyme functionality.

It is noteworthy that aminoperoxide species comparable to the Fl_N5OO_ do not seem to play a role in organic chemistry probably as a result of their instability.^39^ TdaE, however, employs the Fl_N5OO_ for an unusual redox-neutral (nonoxidative) oxygenation that involves the hydrolysis-like formal transfer of an [OH]^−^, which is normally mediated by water-activating enzymes rather than oxygenases. Accordingly, TdaE constitutes, to the best of our knowledge, the first enzyme that oxygenolytically rather than hydrolytically cleaves a CoA thioester bond, analogous to the case of amide bond cleavage by RutA.^35,39,48,49^ The Fl_N5OO_ is a potent soft α-nucleophile that is distinct in reactivity from activated water (i.e., a hard nucleophile).^35,39^ Hence, other RutA-like group C FPMOs expectedly catalyze more demanding Fl_N5OO_-dependent oxygenation reactions, as exemplified by C–Cl bond cleavage (dehalogenation) of hexachlorobenzene by HcbA1 or C–S bond cleavage of dibenzothiophene sulfone by DszA.^35,39^ Key to such “pseudohydrolysis” reactions is the lone pair of electrons of the flavin-N5, which enables the elimination of the oxygenated product as a leaving group from a covalent Fl_N5OO_-substrate adduct via heterolytic cleavage of the O–O bond.^35,39^ This contrasts with classical oxidative oxygenation chemistry by enzymes in which the cofactor serves as leaving group and takes up the electrons during heterolytic peroxide cleavage.^35^ However, while the Fl_N5O_ is seemingly a byproduct of reactions catalyzed by RutA-like enzymes, the proficient TdaE additionally utilizes this species for a second oxidative oxygenation reaction via formal transfer of an [OH]^+^. The Fl_N5O_ corresponds to a nitrone (an oxoammonium in the resonance form) that can be converted to a nitroxyl radical upon single-electron reduction. These functional groups are widely used in synthetic chemistry also for radical and nonradical oxidation and oxygenation reactions, e.g., the nitroxyl radical PINO (phthalimide-*N*-oxyl) or the nitrone TEMPO (2,2,6,6-tetramethylpiperidin-1-yl)oxyl),^35,50^ and it is therefore congruous that enzymes such as EncM and TdaE evolved to exploit the Fl_N5O_ species. On the basis of the electrophilic properties of **17** at the oxygenation site and consistent with our results, we propose a Michael addition of the nucleophilic Fl_N5O_ to the tropone ring that subsequently enables epoxide formation under elimination of Fl_ox_, although a radical mechanism cannot be ruled out.

## Conclusion

In summary, TdaE, ostensibly an inconspicuous member of the ACAD enzyme family, was revealed as a remarkably efficient key tailoring enzyme for the biosynthesis of environmentally and pharmaceutically important tropone natural products from marine and terrestrial bacteria. TdaE catalysis combines classical dehydrogenation with subsequent aminoperoxide and aminoxide/nitrone chemistry to mediate CoA-ester oxygenolysis and ring epoxidation via consecutive chemo- and regioselective oxygen transfer steps. These findings exemplify how a single enzyme can take advantage of the distinct chemical features of two different oxygen transferring species in the form of the Fl_N5OO_ and the Fl_N5O_ species to achieve noncanonical dual oxygenation. Hence, flavin-N5-oxygen adducts in enzymology seem more pervasive and versatile than previously appreciated, and TdaE accordingly represents a new prototype of an internal flavoprotein dioxygenase.

## Supporting Information Available

Experimental procedures and characterization data for the reported compounds, Figures S1–S68, and Tables S1–S4 (PDF)

## References

[ref1] Duan Y.; Petzold M.; Saleem-Batcha R.; Teufel R. Bacterial tropone natural products and derivatives: Overview on the biosynthesis, bioactivities, ecological role and biotechnological potential. ChemBioChem 2020, 21, 2384–2407. 10.1002/cbic.201900786. 32239689 PMC7497051

[ref2] Guo H.; Roman D.; Beemelmanns C. Tropolone natural products. Nat. Prod. Rep. 2019, 36, 1137–1155. 10.1039/C8NP00078F. 30556819

[ref3] Teufel R.; Mascaraque V.; Ismail W.; Voss M.; Perera J.; Eisenreich W.; Haehnel W.; Fuchs G. Bacterial phenylalanine and phenylacetate catabolic pathway revealed. Proc. Natl. Acad. Sci. U. S. A. 2010, 107, 14390–14395. 10.1073/pnas.1005399107. 20660314 PMC2922514

[ref4] Geng H.; Bruhn J. B.; Nielsen K. F.; Gram L.; Belas R. Genetic dissection of tropodithietic acid biosynthesis by marine roseobacters. Appl. Environ. Microbiol. 2008, 74, 1535–1545. 10.1128/AEM.02339-07. 18192410 PMC2258615

[ref5] Teufel R.; Gantert C.; Voss M.; Eisenreich W.; Haehnel W.; Fuchs G. Studies on the mechanism of ring hydrolysis in phenylacetate degradation: a metabolic branching point. J. Biol. Chem. 2011, 286, 11021–11034. 10.1074/jbc.M110.196667. 21296885 PMC3064157

[ref6] Berger M.; Brock N. L.; Liesegang H.; Dogs M.; Preuth I.; Simon M.; Dickschat J. S.; Brinkhoff T. Genetic analysis of the upper phenylacetate catabolic pathway in the production of tropodithietic acid by Phaeobacter gallaeciensis. Appl. Environ. Microbiol. 2012, 78, 3539–3551. 10.1128/AEM.07657-11. 22407685 PMC3346364

[ref7] Cane D. E.; Wu Z.; van Epp J. E. Thiotropocin biosynthesis. Shikimate origin of a sulfur-containing tropolone derivative. J. Am. Chem. Soc. 1992, 114, 8479–8483. 10.1021/ja00048a019.

[ref8] Erb T. J.; Ismail W.; Fuchs G. Phenylacetate metabolism in thermophiles: characterization of phenylacetate-CoA ligase, the initial enzyme of the hybrid pathway in Thermus thermophilus. Curr. Microbiol. 2008, 57, 27–32. 10.1007/s00284-008-9147-3. 18414813

[ref9] El-Said Mohamed M. Biochemical and molecular characterization of phenylacetate-coenzyme A ligase, an enzyme catalyzing the first step in aerobic metabolism of phenylacetic acid in Azoarcus evansii. J. Bacteriol. 2000, 182, 286–294. 10.1128/JB.182.2.286-294.2000. 10629172 PMC94275

[ref10] Martínez-Blanco H.; Reglero A.; Rodriguez-Aparicio L. B.; Luengo J. M. Purification and biochemical characterization of phenylacetyl-CoA ligase from Pseudomonas putida. A specific enzyme for the catabolism of phenylacetic acid. J. Biol. Chem. 1990, 265, 7084–7090. 10.1016/S0021-9258(19)39262-2. 2324116

[ref11] Teufel R.; Friedrich T.; Fuchs G. An oxygenase that forms and deoxygenates toxic epoxide. Nature 2012, 483, 359–362. 10.1038/nature10862. 22398448

[ref12] Grishin A. M.; Ajamian E.; Tao L.; Zhang L.; Menard R.; Cygler M. Structural and functional studies of the Escherichia coli phenylacetyl-CoA monooxygenase complex. J. Biol. Chem. 2011, 286, 10735–10743. 10.1074/jbc.M110.194423. 21247899 PMC3060524

[ref13] Spieker M.; Saleem-Batcha R.; Teufel R. Structural and Mechanistic Basis of an Oxepin-CoA Forming Isomerase in Bacterial Primary and Secondary Metabolism. ACS Chem. Biol. 2019, 14, 2876–2886. 10.1021/acschembio.9b00742. 31689071

[ref14] Grishin A. M.; Ajamian E.; Zhang L.; Rouiller I.; Bostina M.; Cygler M. Protein-protein interactions in the β-oxidation part of the phenylacetate utilization pathway: crystal structure of the PaaF-PaaG hydratase-isomerase complex. J. Biol. Chem. 2012, 287, 37986–37996. 10.1074/jbc.M112.388231. 22961985 PMC3488069

[ref15] Brock N. L.; Nikolay A.; Dickschat J. S. Biosynthesis of the antibiotic tropodithietic acid by the marine bacterium Phaeobacter inhibens. Chem. Commun. 2014, 50, 5487–5489. 10.1039/c4cc01924e. 24723119

[ref16] Wang M.; Hashimoto M.; Hashidoko Y. Repression of tropolone production and induction of a Burkholderia plantarii pseudo-biofilm by carot-4-en-9,10-diol, a cell-to-cell signaling disrupter produced by Trichoderma virens. PLoS One 2013, 8, e78024 10.1371/journal.pone.0078024. 24223754 PMC3817171

[ref17] Azegami K.; Nishiyama K.; Watanabe Y.; Suzuki T.; Yoshida M.; Nose K.; Toda S. Tropolone as a root growth-inhibitor produced by a plant pathogenic Pseudomonas sp. causing seedling blight of rice. Nippon Shokubutsu Byori Gakkaiho 1985, 51, 315–317. 10.3186/jjphytopath.51.315.

[ref18] Azegami K.; Nishiyama K.; Kato H. Effect of Iron Limitation on ″Pseudomonas plantarii″ Growth and Tropolone and Protein Production. Appl. Environ. Microbiol. 1988, 54, 844–847. 10.1128/aem.54.3.844-847.1988. 16347592 PMC202556

[ref19] Jiang Z.; Chen M.; Yu X.; Xie Z. 7-Hydroxytropolone produced and utilized as an iron-scavenger by Pseudomonas donghuensis. BioMetals 2016, 29, 817–826. 10.1007/s10534-016-9954-0. 27542164

[ref20] Muzio F. M.; Agaras B. C.; Masi M.; Tuzi A.; Evidente A.; Valverde C. 7-hydroxytropolone is the main metabolite responsible for the fungal antagonism of Pseudomonas donghuensis strain SVBP6. Environ. Microbiol. 2020, 22, 2550–2563. 10.1111/1462-2920.14925. 31984618

[ref21] Chen X.; Xu M.; Lü J.; Xu J.; Wang Y.; Lin S.; Deng Z.; Tao M. Biosynthesis of Tropolones in Streptomyces spp.: Interweaving Biosynthesis and Degradation of Phenylacetic Acid and Hydroxylations on the Tropone Ring. Appl. Environ. Microbiol. 2018, 84, e00349–18. 10.1128/AEM.00349-18. 29654178 PMC5981077

[ref22] Berger M.; Neumann A.; Schulz S.; Simon M.; Brinkhoff T. Tropodithietic acid production in Phaeobacter gallaeciensis is regulated by N-acyl homoserine lactone-mediated quorum sensing. J. Bacteriol. 2011, 193, 6576–6585. 10.1128/JB.05818-11. 21949069 PMC3232910

[ref23] Seyedsayamdost M. R.; Carr G.; Kolter R.; Clardy J. Roseobacticides: small molecule modulators of an algal-bacterial symbiosis. J. Am. Chem. Soc. 2011, 133, 18343–18349. 10.1021/ja207172s. 21928816 PMC3211371

[ref24] Korth H.; Brüsewitz G.; Pulverer G. Isolierung eines antibiotisch wirkenden Tropolons aus einem Stamm von Pseudomonas cepacia. Zentralbl. Bakteriol., Mikrobiol. Hyg., Abt. 1, Orig. A 1982, 252, 83–86. 10.1016/S0174-3031(82)80090-5. 6812315

[ref25] Rabe P.; Klapschinski T. A.; Brock N. L.; Citron C. A.; D’Alvise P.; Gram L.; Dickschat J. S. Synthesis and bioactivity of analogues of the marine antibiotic tropodithietic acid. Beilstein J. Org. Chem. 2014, 10, 1796–1801. 10.3762/bjoc.10.188. 25161739 PMC4142847

[ref26] Seyedsayamdost M. R.; Case R. J.; Kolter R.; Clardy J. The Jekyll-and-Hyde chemistry of Phaeobacter gallaeciensis. Nat. Chem. 2011, 3, 331–335. 10.1038/nchem.1002. 21430694 PMC3376411

[ref27] Azegami K.; Nishiyama K.; Watanabe Y.; Kadota I.; Ohuch A.; Fukazawa C. Pseudomonas plantarii sp. nov., the Causal Agent of Rice Seedling Blight. Int. J. Syst. Bacteriol. 1987, 37, 475 10.1099/00207713-37-4-475.

[ref28] Ham J. H.; Melanson R. A.; Rush M. C. Burkholderia glumae: next major pathogen of rice?. Mol. Plant Pathol. 2011, 12, 329–339. 10.1111/j.1364-3703.2010.00676.x. 21453428 PMC6640401

[ref29] Wang M.; Tachibana S.; Murai Y.; Li L.; Lau S. Y. L.; Cao M.; Zhu G.; Hashimoto M.; Hashidoko Y. Indole-3-Acetic Acid Produced by Burkholderia heleia Acts as a Phenylacetic Acid Antagonist to Disrupt Tropolone Biosynthesis in Burkholderia plantarii. Sci. Rep. 2016, 6, 22596 10.1038/srep22596. 26935539 PMC4776283

[ref30] Rost R.; Haas S.; Hammer E.; Herrmann H.; Burchhardt G. Molecular analysis of aerobic phenylacetate degradation in Azoarcus evansii. Mol. Genet. Genomics 2002, 267, 656–663. 10.1007/s00438-002-0699-9. 12172805

[ref31] Sonnenschein E. C.; Phippen C. B. W.; Bentzon-Tilia M.; Rasmussen S. A.; Nielsen K. F.; Gram L. Phylogenetic distribution of roseobacticides in the Roseobacter group and their effect on microalgae. Environ. Microbiol. Rep. 2018, 10, 383–393. 10.1111/1758-2229.12649. 29624899

[ref32] Wang R.; Gallant É.; Seyedsayamdost M. R. Investigation of the Genetics and Biochemistry of Roseobacticide Production in the Roseobacter Clade Bacterium Phaeobacter inhibens. mBio 2016, 7, e02118 10.1128/mBio.02118-15. 27006458 PMC4807370

[ref33] Dickschat J. S.; Rinkel J.; Klapschinski T.; Petersen J. Characterisation of the l-Cystine β-Lyase PatB from Phaeobacter inhibens: An Enzyme Involved in the Biosynthesis of the Marine Antibiotic Tropodithietic Acid. ChemBioChem 2017, 18, 2260–2267. 10.1002/cbic.201700358. 28895253

[ref34] Schoenian I.; Paetz C.; Dickschat J. S.; Aigle B.; Leblond P.; Spiteller D. An unprecedented 1,2-shift in the biosynthesis of the 3-aminosalicylate moiety of antimycins. ChemBioChem 2012, 13, 769–773. 10.1002/cbic.201200033. 22378565

[ref35] Toplak M.; Matthews A.; Teufel R. The devil is in the details: The chemical basis and mechanistic versatility of flavoprotein monooxygenases. Arch. Biochem. Biophys. 2021, 698, 108732 10.1016/j.abb.2020.108732. 33358998

[ref36] Palfey B. A.; McDonald C. A. Control of catalysis in flavin-dependent monooxygenases. Arch. Biochem. Biophys. 2010, 493, 26–36. 10.1016/j.abb.2009.11.028. 19944667

[ref37] Chaiyen P.; Fraaije M. W.; Mattevi A. The enigmatic reaction of flavins with oxygen. Trends Biochem. Sci. 2012, 37, 373–380. 10.1016/j.tibs.2012.06.005. 22819837

[ref38] Huijbers M. M. E.; Montersino S.; Westphal A. H.; Tischler D.; van Berkel W. J. H. Flavin dependent monooxygenases. Arch. Biochem. Biophys. 2014, 544, 2–17. 10.1016/j.abb.2013.12.005. 24361254

[ref39] Matthews A.; Saleem-Batcha R.; Sanders J. N.; Stull F.; Houk K. N.; Teufel R. Aminoperoxide adducts expand the catalytic repertoire of flavin monooxygenases. Nat. Chem. Biol. 2020, 16, 556–563. 10.1038/s41589-020-0476-2. 32066967

[ref40] Teufel R.; Miyanaga A.; Michaudel Q.; Stull F.; Louie G.; Noel J. P.; Baran P. S.; Palfey B.; Moore B. S. Flavin-mediated dual oxidation controls an enzymatic Favorskii-type rearrangement. Nature 2013, 503, 552–556. 10.1038/nature12643. 24162851 PMC3844076

[ref41] Teufel R.; Stull F.; Meehan M. J.; Michaudel Q.; Dorrestein P. C.; Palfey B.; Moore B. S. Biochemical Establishment and Characterization of EncM’s Flavin-N5-oxide Cofactor. J. Am. Chem. Soc. 2015, 137, 8078–8085. 10.1021/jacs.5b03983. 26067765 PMC4720136

[ref42] Saleem-Batcha R.; Stull F.; Sanders J. N.; Moore B. S.; Palfey B. A.; Houk K. N.; Teufel R. Enzymatic control of dioxygen binding and functionalization of the flavin cofactor. Proc. Natl. Acad. Sci. U. S. A. 2018, 115, 4909–4914. 10.1073/pnas.1801189115. 29686059 PMC5949001

[ref43] Romero E.; Gómez Castellanos J. R.; Gadda G.; Fraaije M. W.; Mattevi A. Same Substrate, Many Reactions: Oxygen Activation in Flavoenzymes. Chem. Rev. 2018, 118, 1742–1769. 10.1021/acs.chemrev.7b00650. 29323892

[ref44] Frensch B.; Lechtenberg T.; Kather M.; Yunt Z.; Betschart M.; Kammerer B.; Lüdeke S.; Müller M.; Piel J.; Teufel R. Enzymatic spiroketal formation via oxidative rearrangement of pentangular polyketides. Nat. Commun. 2021, 12, 1431 10.1038/s41467-021-21432-9. 33664266 PMC7933358

[ref45] Teufel R. Flavin-catalyzed redox tailoring reactions in natural product biosynthesis. Arch. Biochem. Biophys. 2017, 632, 20–27. 10.1016/j.abb.2017.06.008. 28619619

[ref46] Paul C. E.; Eggerichs D.; Westphal A. H.; Tischler D.; van Berkel W. J. H. Flavoprotein monooxygenases: Versatile biocatalysts. Biotechnol. Adv. 2021, 107712 10.1016/j.biotechadv.2021.107712. 33588053

[ref47] Alfieri A.; Fersini F.; Ruangchan N.; Prongjit M.; Chaiyen P.; Mattevi A. Structure of the monooxygenase component of a two-component flavoprotein monooxygenase. Proc. Natl. Acad. Sci. U. S. A. 2007, 104, 1177–1182. 10.1073/pnas.0608381104. 17227849 PMC1783134

[ref48] Adak S.; Begley T. P. RutA-Catalyzed Oxidative Cleavage of the Uracil Amide Involves Formation of a Flavin-N5-oxide. Biochemistry 2017, 56, 3708–3709. 10.1021/acs.biochem.7b00493. 28661684 PMC6040642

[ref49] Mukherjee T.; Zhang Y.; Abdelwahed S.; Ealick S. E.; Begley T. P. Catalysis of a flavoenzyme-mediated amide hydrolysis. J. Am. Chem. Soc. 2010, 132, 5550–5551. 10.1021/ja9107676. 20369853 PMC2873085

[ref50] Coseri S. Phthalimide- N -oxyl (PINO) Radical, a Powerful Catalytic Agent: Its Generation and Versatility Towards Various Organic Substrates. Catal. Rev.: Sci. Eng. 2009, 51, 218–292. 10.1080/01614940902743841.

