# Supplementary material for: A Flavoprotein Dioxygenase Steers Bacterial Tropone Biosynthesis via Coenzyme A-Ester Oxygenolysis and Ring Epoxidation
Source: J Am Chem Soc. 2021 Jul 1;143(27):10413–21. doi: 10.1021/jacs.1c04996 (PMC8283759; doi:10.1021/jacs.1c04996)
Supplement: Supplementary file 1 — ja1c04996_si_001.pdf [file ja1c04996_si_001.pdf]

Supporting Information

A flavoprotein dioxygenase steers bacterial tropone biosynthesis via coenzyme A-ester oxygenolysis and ring epoxidation

Authors: Ying Duan[a,1], Marina Toplak[a,1], Anwei Hou[b,1], Nelson L Brock[c], Jeroen S. Dickschat[b,c,*], Robin Teufel[a,*]

[a]Faculty of Biology, University of Freiburg, Schänzlestrasse 1, 79104 Freiburg, Germany.

[b]Kekulé-Institute of Organic Chemistry and Biochemistry, University of Bonn, Gerhard-Domagk-Strasse 1, 53121 Bonn, Germany.
[c]Institute of Organic Chemistry, TU Braunschweig, Hagenring 30, 38106 Braunschweig, Germany.

[1]these authors contributed equally

[*]correspondence to: Dr. Robin Teufel (robin.teufel@zbsa.uni-freiburg.de) or
Prof. Dr. Jeroen S. Dickschat (dickschat@uni-bonn.de)

Experimental Procedures

General (Biochemical investigation)

All chemicals and reagents used were purchased from Carl Roth (Karlsruhe, Germany), Sigma-Aldrich (St. Louis, MO, USA), Fisher Scientific (Hampton, NH, USA) and Biomol (Hamburg, Germany). Enzymes and materials used for molecular cloning were obtained from New England Biolabs (NEB), Thermo Fisher Scientific (Waltham, MA, USA), and Qiagen (Hilden, Germany) and oligonucleotides (PCR-primers) were purchased from Sigma Aldrich. For protein purification and concentration, equipment (Ni-NTA-columns, gelfiltration columns) from GE Healthcare/Cytiva (Chicago, IL, USA) and PALL (New York, USA) was used. Agarose- and SDS-PAGE analysis was performed using devices from Bio-Rad (Hercules, CA, USA).

Cloning and recombinant production –TdaE$^{\mathbf{Pi}}$

For the recombinant production of TdaE^{Pi} (NCBI-accession number: WP_014881725.1) the respective gene was purchased from BioCat (Heidelberg, Germany), codon optimized for *Escherichia coli*. After restriction digestion with NcoI (5') and NotI (3'), the gene was cloned into the pET-M11-His-TEV vector, which additionally contained a coding sequence for the solubility enhancer protein MBP (maltose binding protein) between the hexahistidine-tag and the tobacco etch virus (TEV)-cleavage site. Having confirmed the proper insertion of the *tdaE*^{Pi} gene into the vector of interest by automated sequencing, the recombinant plasmid was transformed into *E. coli* BL21 (DE3)-cells (Thermo Fisher Scientific, Waltham, MA, USA). For gene expression, TB-medium supplemented with kanamycin (50 µg mL^{-1}) was inoculated with a pre-culture grown in LB-medium (supplemented with the same amount of antibiotic) to an OD_{600} of ~0.1 and incubated at 37 °C and 130 rpm until an OD_{600} of ~0.6 was reached. Then, the temperature in the incubator was set to 18 °C and protein production was induced by the addition of 0.5 mM IPTG. After overnight incubation at 18 °C and 130 rpm, cells were harvested by centrifugation (4000 g for 15 min).

Protein purification - TdaE$^{\mathbf{Pi}}$

For protein purification, cells were resuspended in 20 mM Tris, 200 mM KCl, 10 % glycerol pH 8 + 10 µM FAD (buffer A) and a spatula tip of FAD (Roth, Germany), 1 mg mL^{-1} lysozyme and 0.1 mg mL^{-1} DNase were added. After 30 min of incubation on ice, cells were lysed by ultrasonication (1 s pulse, 3 s pause; 3 min pulse time, 2 times). Then, the lysate was cleared by centrifugation (18 000 g for 30 min), filtered, and loaded onto MBP-trap columns (Cytiva, Waltham, MA, USA) pre-equilibrated with buffer A. Unspecifically bound proteins were removed by washing with about 10 column volumes of buffer A and the MBP-tagged TdaE^{Pi} was eluted using a linear gradient of buffer A and buffer B (20 mM Tris, 200 mM KCl, 10 mM maltose, 10 % glycerol pH 8; 0 % B to 100 % B within 6 column volumes). The protein-containing fractions were pooled and concentrated (to ~250 µM, 1 eq) and 2 eq of FAD (~500 µM) were added. Finally, the protein was flash-frozen in liquid nitrogen and stored at -80 °C until further use.

Cloning and recombinant production –TdaE$^{\mathbf{Bp}}$

For the recombinant production of TdaE^{Bp} (NCBI-accession number: WP_042624079.1), the respective gene was purchased from BioCat (Heidelberg, Germany), codon optimized for *Escherichia coli*. After restriction digestion with NcoI (5') and NotI (3'), the gene was cloned into the pET-M11-His-TEV vector, which additionally contained a coding sequence for the solubility enhancer protein GB1 (B1-domain of protein G) between the hexahistidine-tag and the tobacco etch virus (TEV)-cleavage site. Furthermore, the sequence was cloned in frame with a C-terminal histidine-tag, which turned out to be crucial for the solubility and stability of the protein as well. Having confirmed the proper insertion of

the *tdaE*^{Bp} gene into the vector of interest by automated sequencing, the recombinant plasmid was transformed into *E. coli* BL21 (DE3)-cells (Thermo Fisher Scientific, Waltham, MA, USA).

For gene expression, TB-medium supplemented with kanamycin (50 µg mL^{-1}) was inoculated with a pre-culture grown in LB-medium (supplemented with the same amount of antibiotic) to an OD_{600} of ~0.1 and incubated at 37 °C and 130 rpm until an OD_{600} of ~0.5 was reached. Then, the temperature in the incubator was set to 18 °C and protein production was induced by the addition of 0.1 mM IPTG at an OD_{600} of ~0.7-0.8. After overnight incubation at 18°C and 130 rpm, cells were harvested by centrifugation (4000 g for 15 min).

Protein purification –TdaE^{Bp}

For protein purification, cells were resuspended in binding buffer (50 mM Tris, 300 mM NaCl, 10 % glycerol, pH 7.4) containing a spatula tip of FAD (Roth, Germany) and lysed by ultrasonication (3 s pulse, 2 s pause, 5 min pulse time). Having cleared the lysate by centrifugation (18000 g for 40 min), the latter was loaded onto a 5 mL Ni-NTA FF crude column (Cytiva, Marlborough, MA, USA) pre-equilibrated with binding buffer. Unspecifically bound proteins were washed from the column using wash buffer (50 mM Tris, 300 mM NaCl, 30 mM imidazole, 10 % glycerol, pH 7.4), before eluting TdaE^{Bp} with elution buffer (50 mM Tris, 300 mM NaCl, 500 mM imidazole, 10 % glycerol, pH 7.4). Yellow fractions were combined and concentrated and buffer was exchanged to wash buffer (NOTE: the protein precipitates upon re-buffering to binding buffer) using desalting columns (HiTrap Desalt, Cytiva). Finally, the protein was concentrated to ~60 µM (cofactor concentration), flash-frozen in liquid N_2 and stored at -80°C until further use.

Cloning, recombinant production and protein purification – putative decarboxylase

Cloning and recombinant production of the putative decarboxylase (NCBI-accession number: WP_052498255.1; MBP-tagged) from *Burkholderia plantarii* was performed as described for TdaE^{Pi}.

For protein purification, cells were resuspended in 20 mM Tris, 200 mM KCl, 10 % glycerol pH 8 (buffer A) and lysed by ultrasonication (3 s pulse, 2 s pause; 5 min pulse time). Then, the lysate was cleared by centrifugation (18 000 g for 40 min) and loaded onto a 5 mL MBP-trap column pre-equilibrated with buffer A. Unspecifically bound proteins were removed by washing with about 10 column volumes of buffer A and the MBP-tagged decarboxylase was eluted using a linear gradient of buffer A and buffer B (20 mM Tris, 200 mM KCl, 10 mM maltose, 10 % glycerol pH 8; 0 % B to 100 % B within 5 column volumes). The most concentrated protein fraction was flash-frozen in liquid nitrogen and stored at -80 °C until further use.

Recombinant production and purification – PaaABCE, PaaG, PaaZ-E256Q, PaaY, flavin reductase (Fre)

To obtain pure PaaABCE, PaaG, PaaZ-E256Q, PaaY and Fre, their recombinant production and purification was carried out as described previously[1–3].

Recombinant production and purification – PaaK^{Tt}

PaaK from *Thermus thermophilus* (PaaK^{Tt}) was recombinantly produced as described previously[4] and subsequently partially purified by heat precipitation. Accordingly, harvested cells were resuspended in 20 mM Tris, 250 mM KCl, pH 8 + 0.1 mg mL^{-1} DNase and lysed by ultrasonication (0.5 s pulse, 1.5 s pause, 1 min pulse time, 3 times). After 30 min of centrifugation (18 000 g), the cleared lysate was incubated at 75 °C for 10 min and denatured proteins were removed by another 10 min of centrifugation (denaturation and centrifugation was repeated once). Finally, the clear protein containing supernatant was flash frozen in liquid nitrogen and stored at -80 °C until further use.

Analytical size-exclusion chromatography

To determine the oligomeric state of the different proteins in solution, analytical size-exclusion chromatography was carried out using a Superdex 200 GL 10/300 column (Cytiva) pre-equilibrated with 20 mM Tris, 200 mM KCl, 10 % glycerol pH 8 (for TdaE^{Pi} additionally 10 µM FAD were added to the buffer) or 50 mM Tris, 300 mM NaCl, pH 7.4 (for TdaE^{Bp} only). Molecular weights of the proteins eluting in the main peak fractions were estimated based on a previously generated calibration curve.

Determination of the molar extinction coefficient of TdaE^{Bp}-bound FAD

To determine the molar extinction coefficient of the FAD-cofactor bound to TdaE^{Bp}, UV-visible absorption spectra of the native protein solution as well as of the enzyme denatured with 0.5 % SDS were recorded between 300 and 800 nm. By assuming that the molar extinction coefficient of the FAD cofactor in the denatured protein sample equals the one of free FAD (ε_{450}: 11 300 $M^{-1}cm^{-1}$), the extinction coefficient of TdaE^{Bp}-bound FAD at 450 nm was calculated to ~14 000 M^{-1} cm^{-1}.

Multiple sequence alignment TdaE versus classical acyl-CoA dehydrogenases

Sequences for the alignment were chosen based on the result of a blastp of the amino acid sequence of TdaE^{Pi} against all non-redundant protein sequences deposited in the NCBI database. One amino acid sequence per species (within the best 100 hits) was selected and amino acid sequences of classical acyl CoA dehydrogenases (SCAD and IVD) from *P. inhibens*, *B. plantarii* and *R. norvegicus* were added. Then, a structure-based alignment was generated using the online program T-coffee[5] and visualized using SeaView[6]. Final color editing to highlight important (catalytic) residues was carried out in PowerPoint.

Homology modeling TdaE^{Pi}

A homology model of TdaE^{Pi} was generated based on the crystal structure of rat short chain acyl CoA-dehydrogenase (SCAD, PDB-ID: 1jqi) using the SWISSmodel-server[7]. This template was chosen, as SWISSmodel predicted both the highest sequence identity (26 %), as well as the highest sequence coverage (95 %) of TdaE^{Pi} with/to SCAD compared to all other protein structures deposited in the PDB.

Chemical synthesis of phenylacetyl (Pa)-CoA (5)

Unlabeled Pa-CoA (**5**) was chemically synthesized from phenylacetyl succinimide and coenzyme A as described previously[1,8] and purified by means of preparative HPLC. The filtered sample was applied to a Eurospher II 100-5 C18 column (250x20 mm; KNAUER Wissenschaftliche Geräte GmbH) equilibrated with 98 % 10 mM ammonium acetate (pH 4.5) and 2 % acetonitrile at a flow of 8 mL min^{-1}. The column was developed by a linear gradient from 2 % acetonitrile to 50 % acetonitrile in 10 mM ammonium acetate buffer (pH 4.5) within 30 min. Elution was monitored at 260 nm with an UV diode array detector.

***Production of $^{13}C_2$ and $^{13}C_8$-labeled** 5*

To produce $^{13}C_2$- and $^{13}C_8$-labeled **5**, $^{13}C_2$- (Sigma, St. Louis, MO, USA) and $^{13}C_8$-phenylacetate (chemically synthesized, see below) (0.5 mM), respectively, was mixed with 1 mM CoA, 2 mM ATP, 2 mM $MgCl_2$ and 10 µM PaaK^{Tt} (200 mL assay volume) and incubated at 75 °C for 5 min. Then, the reaction was quenched with 200 mL of EtOAc and the water phase was flash frozen and lyophilized overnight. The following day, the residue was re-dissolved in buffer and purified by means of preparative HPLC. Isolated **5** was again flash frozen in liquid N_2 and solvents were removed by lyophilization for around 60 h (yield: ~ 25 mg, ~ 0.028 mmol).

Timecourse TdaE^Pi^

To produce **4**, a reaction mixture containing 0.5 mM Pa-CoA (**5**), 1.5 mM NADPH, 1 µM PaaG, 1.5 µM PaaZ-E256Q and 1 µg μL^{-1} PaaABCE was prepared in 50 mM Tris-HCl pH 8 and incubated at 30 °C and 900 rpm for 10 min. Then, a sample was taken (50 µL), mixed with an equal volume of 50 mM Tris-HCl pH 8 and quenched with abs. MeOH (200 µL; 0 min time point). Subsequently, 400 µL of the substrate mixture were either mixed with an equal volume of 50 mM Tris-HCl pH 8 (control reaction) or an equal volume of a 2 µM $TdaE^{Pi}$-solution (in 50 mM Tris-HCl pH 8; 1 µM final enzyme concentration) and the samples were again incubated at 30 °C and 900 rpm. 100 µL of sample were withdrawn from each tube after 2, 5, 15, 30, 45, and 60 min and quenched in 200 µL of abs. MeOH. After 10 min of centrifugation at 20 000 g, the supernatants were transferred to fresh reaction tubes and dried in the speed-vac for 1.75 h. Finally, the sample volume was adjusted to 140 µL using reaction buffer and all samples were analyzed by HPLC-DAD.

***Enzymatic production and isolation of** 16*

To be able to isolate pure **16** for structure confirmation as well as for additional enzyme assays, **4** was produced as described above (see "Timecourse $TdaE^{Pi}$") and mixed with 3 µM $TdaE^{Pi}$ (10 ml assay). After 10 min of incubation at 30 °C and 900 rpm, reactions were quenched with double volume of MeOH and centrifuged at 20 000 rpm for 10 min. Supernatants were concentrated in the speed-vac and **16** was purified by HPLC (manual fractionation). Isolated **16** was flash frozen in liquid N_2 immediately and solvent was removed by lyophilization – yield: ~ 0.3 mg, ~ 0.33 µmol.

*16**-turnover by PaaY***

To confirm its structure, purified **16** (75 µM) was mixed with 1 µM of the thioesterase PaaY and incubated at 30 °C and 900 rpm for 10 min. Then, putative CoA-free reaction products were extracted with EtOAc + 1 % formic acid (FA) and the organic layer was concentrated in the speed-vac for 5 min. Finally, the sample was analyzed by HPLC-DAD as well as UPLC-MS.

***Production of $^{13}C_2$ and $^{13}C_8$-labeled** 18*

To produce $^{13}C_2$- or $^{13}C_8$-labeled **18** for structure elucidation by NMR, reaction mixtures containing 0.5 mM $^{13}C_2$- or $^{13}C_8$-labeled **5**, 1.5 mM NADPH, 1 µg μL^{-1} PaaABCE, 1 µM PaaG and 1.5 µM PaaZ-E256Q were prepared in 50 mM Tris-HCl pH 8 (5 x 10 mL in round bottom flasks) and stirred at 30 °C for 10 min. Then, $TdaE^{Pi}$ was added to a final concentration of 3 µM and the reaction was stirred for additional 30 min. Reaction products were extracted with EtOAc +1 % FA (20 mL per reaction) and the combined organic layers were concentrated to ~2.5 mL. Subsequently, **18** was purified by HPLC (manual collection), again extracted with EtOAc +1 % FA and concentrated to 200-300 µL. Since **18** turned out to be extremely unstable and volatile, resulting in a tremendous sample-loss upon concentration, slow solvent exchange to CD_3CN was carried out, by twice diluting the sample with 300 µL of CD_3CN and concentrating it again to a final volume of ~300 µL.

Turnover assay with $TdaE^{Bp}$

To produce the substrate (**4**) for $TdaE^{Bp}$, a reaction mixture containing 1.5 mM NADPH, 1 µg μL^{-1} PaaABCE, 1 µM PaaG and 1.5 µM PaaZ-E256Q was prepared in 50 mM Tris-HCl pH 8 and incubated at 30 °C for 2 min. Then, Pa-CoA (**5**, 1 mM final concentration) was added to initiate turnover and the reaction was incubated at 30 °C and 900 rpm. After 10 min, a sample was taken and quenched with abs. MeOH (0 min time point) and $TdaE^{Bp}$ (250 nM final concentration) was added to the remaining reaction mixture. Samples were taken after 30 s, 1 min, 2 min, 4.5 min, 7 min and 10 min and again quenched with abs. MeOH. Subsequently, all samples were centrifuged for 10 min (18 000 g) and the supernatants were dried in the speed-vac for 1.5 h. Finally, the sample volume was adjusted to 140 µL using reaction buffer and all samples were analyzed by HPLC-DAD.

Effect of L-ascorbate (L-AA) and 5,5-dimethyl-1-pyrroline-N-oxide (DMPO) on *18* ***formation***

To study the effect of two radical scavengers on **18** formation, **4** was produced as described above (see "Turnover assay with TdaE^{Bp}") and mixed with 5 or 10 mM L-AA/DMPO and 1 µM TdaE^{Pi} (final volume, 100 µL). After 30 min incubation at 30°C and 900 rpm, the reactions were quenched and extracted with 200 µL EtOAc + 1 % FA and the organic layers were dried in the speed-vac for 5 min. Finally, the sample volume was adjusted to 90 µL using EtOAc and the samples were analyzed by HPLC-DAD.

Enzymatic production of (Z)-2-(oxepin-2(3H)-ylidene)-acetyl-CoA ("oxepin-CoA", *7)*

For the enzymatic production of **7**, 0.5 mM Pa-CoA (**5**), 1.5 mM NADPH, 1 µg µL^{-1} PaaABCE and 1 µM PaaG were mixed (20 x 200 µL; 4 mL total) and incubated at 30°C and 900 rpm for 8 min. Then, the reactions were quenched with 400 µL of abs. MeOH, each, and centrifuged at 20 000 g for 10 min. Having transferred the supernatants to fresh reaction tubes, the samples were dried in the speed-vac for 1.5 h and **7** was separated from the assay mixture by HPLC (manual fractionation). Isolated **7** was flash frozen in liquid N_2 immediately after collection and solvent was removed by lyophilization (overnight) – yield: ~ 0.5 mg, ~ 0.55 µmol.

16-***turnover by TdaE^{Pi} in the presence of flavin reductase***

To find out whether TdaE^{Pi} is able to convert **16** into **18** in the presence reduced FAD, purified **16** (75 µM) was mixed with 3 µM flavin reductase, 1 mM NADPH, 1 mM NADH and 20 µM TdaE^{Pi} (in the control reaction buffer was added instead of TdaE^{Pi}) in 50 mM Tris-HCl pH 8 (200 µL assay volume) and incubated at 30 °C and 900 rpm for 30 min. Subsequently, the reactions were quenched and extracted with 400 µL of EtOAc + 1 % FA and the organic layers were dried in the speed-vac for 5-10 min. Finally, the volume was adjusted to 80 µL using EtOAc and the samples were analyzed by HPLC-DAD.

Enzymatic production of *18* ***by using*** *7* ***as substrate for the*** **in situ** ***generation of*** *4*

To be able to test the activity of TdaE^{Pi} in the absence of NADPH, **7** was used as alternative substrate for the *in situ* generation of **4**. In this case, **4** was produced by mixing **7** (250 µM) with 5 µM PaaZ-E256Q and incubating the reaction at room temperature (23 °C) for 10 min. Then, TdaE^{Pi} was added to a final concentration of 3 µM (200 µL assay volume) and the sample was incubated at 30 °C and 900 rpm for 30 min. Reaction products were extracted with 400 µL of EtOAc + 1 % FA and the organic layer was concentrated in the speed-vac for 5-10 min. Subsequently, **18** was purified by HPLC, again extracted with EtOAc + 1 % FA and concentrated to ~30 µL, before subjecting it to HR-UPLC-MS analysis.

$H_2{}^{18}O$ labeling assay

To test the incorporation of ^{18}O from $H_2{}^{18}O$ into **18**, at first, an assay mixture containing 250 µM **7** and 5 µM PaaZ-E256Q was prepared in 50 mM Tris-HCl (50 % $H_2{}^{18}O$) and incubated at 30 °C and 900 rpm for 10 min. Then, a sample (100 µL) was taken and quenched in 200 µL of abs. MeOH (control sample, PaaZ-E256Q product) and TdaE^{Pi} was added to a final concentration of 3 µM (200 µL reaction volume). After 30 min of incubation at 30 °C and 900 rpm, reaction products were extracted with EtOAc + 1 % FA and the organic layer was concentrated in the speed-vac for 5 min. Subsequently, **18** was purified by HPLC, again extracted with EtOAc + 1 % FA and concentrated to ~30 µL, before subjecting it to HR-UPLC-MS to analyze ^{18}O incorporation. The control sample, after quenching with abs. MeOH, was centrifuged at 20 000 g for 10 min and the supernatant was dried in the speed-vac for 1.5 h. Finally, also this sample was analyzed for ^{18}O incorporation by HR-UPLC-MS.

$^{18}O_2$ labeling assay

To test the incorporation of ^{18}O from $^{18}O_2$ into **18**, at first, **4** was produced by mixing **7** (250 µM) with 5 µM PaaZ-E256Q under anoxic conditions (glove box) in 50 mM Tris-HCl pH 8 and incubating the reaction at room temperature (23 °C) for 10 min. Then, TdaE^{Pi} was added to a final concentration of 3 µM (200 µL assay volume) and the reaction tube was transferred to an anaerobic air-tight bottle equipped with a septum. Upon removal of the sealed bottle from the glove box, ~2 mL of $^{18}O_2$ were injected into the vessel using a syringe and the reaction was incubated at 30 °C and 900 rpm for 30 min. Reaction products were extracted with 400 µL of EtOAc + 1 % FA and the organic layer was concentrated in the speed-vac for 5-10 min. Subsequently, **18** was purified by HPLC, again extracted with EtOAc + 1 % FA and concentrated to ~30 µL, before subjecting it to HR-UPLC-MS to analyze ^{18}O incorporation.

Trapping of the enzymatically produced FAD-N$_5$-oxide

First, compound **4** was produced as described above (see "Timecourse TdaE^{Pi}"). Then, TdaE^{Pi} with bound FAD (20 µM) or free FAD (20 µM) was added to the compound **4** mixture (sample and control, respectively) and assays were incubated at 10 °C for 10 s to trap the transient FAD-N5-oxide in the reaction. Assays were quenched with EtOAc + 1 % FA and the water phases of both sample and control were analyzed by UPLC-HRMS in positive ion mode.

Activity test of putative decarboxylase

After production of compound **4** as described above (see "Timecourse TdaE^{Pi}"), 3 µM TdaE^{Pi} were added to the compound **4** mixture (200 µL assay volume) and the reaction was incubated at 30 °C and 900 rpm for 30 min to obtain compound **18**. Then, 10 µM of the putative decarboxylase (or an equal volume of 50 mM Tris-HCl pH 8 for the control) were added to the reaction and the enzymatic assays were incubated at 30 °C and 900 rpm for additional 20 min. Reactions were quenched and extracted with 400 µL of EtOAc + 1 % FA and organic layers were concentrated using a speedvac. Finally, samples were analyzed by HPLC-DAD.

Microorganisms and media

The strains used in this study are *Phaeobacter inhibens* DSM 17395 (Prof. Dr. Jeroen S. Dickschat, University of Bonn) and *Burkholderia plantarii* DSM 9509 (obtained from the German Collection of Microorganisms and Cell Cultures, DSMZ). *Phaeobacter inhibens* DSM 17395 was routinely grown in 2216 marine broth as recommended by the manufacturer (Carl Roth, Germany) at 28 °C, 160 rpm. Pre-cultures of *Burkholderia plantarii* DSM 9509 were grown in PY medium (1 % peptone ex casein, 0.5 % yeast extract, 0.5 % NaCl, w/v, pH 7.0) overnight (30 °C, 100 rpm) and then transferred to AG medium (0.1 % $NH_4H_2PO_4$, 0.02 % $MgSO_4 \cdot 7\ H_2O$, 1 % glucose, w/v, pH 7.0) additionally containing 2 mM phenylacetate (the glucose solution was separately sterilized by filtration[9]). Main cultures were incubated at 30 °C and 160 rpm for 24-48 h and cells were harvested by centrifugation (20 000 g for 10 min).

Construction of* P. inhibens ΔtdaE *mutant strain

For deletion of *tdaE* in *P. inhibens* DSM 17395, primers containing restriction sites were designed to amplify chromosomal fragments upstream and downstream of the *tdaE* gene (for oligonucleotide sequences see Table 1 below). The amplified upstream region was cloned between the EcoRI and SmaI restriction sites of the pUC19 multiple cloning site. The amplified downstream region was cloned between the SmaI and HindIII sites of the previously obtained plasmids carrying the respective upstream region. The kanamycin resistance gene (amplified from pBBR1MCS-2 and digested with XmaI) was cloned into the XmaI site of the respective plasmids that already carried the upstream and downstream

regions. *P. inhibens* DSM 17395 was transformed by electroporation with the resulting plasmid, leading to the strain NB05 (Δ*tdaE*::Km).

Table 1: Primers designed and used to amplify chromosomal fragments upstream and downstream of the *tdaE* gene.

Primer name	Oligonucleotide sequence (5' to 3')
**tdaE-LA_EcoRI (upstream)**	CACGCCATTCGTGAATTCATGACCC
**tdaE-LA_SmaI (upstream)**	GGGATCAGGCGGTTTCCTGAAAAGC
**tdaE-RA_SmaI (downstream)**	GGGCGATCGTGGCTACAGCTG
**tdaE-RA_HindIII (downstream)**	CAGTCTGTAAGCTTGCGATAGGAGG

***Cell-free lysate assay* (Phaeobacter inhibens)**
For the cell lysate assays with *Phaeobacter inhibens*, bacterial cultures were harvested after 21 h cultivation, during its exponential growth period. Cells were resuspended in 50 mM Tris-HCl pH 8.0 and lysed by ultrasonication (0.5 s pulse, 1.5 s pause, 1 min pulse time, 3 times). The cleared lysate after centrifugation at 18 000 g for 10 min was used for the assay. To produce **4** or **($^{13}C_8$)-4**, a reaction mixture containing 0.5 mM Pa-CoA (**5** or **($^{13}C_8$)-5**), 1.5 mM NADPH, 1 µM PaaG, 1.5 µM PaaZ-E256Q and 1 µg µL^{-1} PaaABCE was prepared in 50 mM Tris-HCl pH 8 and incubated at 30 °C and 900 rpm for 10 min. Then, 300 µL of compound **4** or **($^{13}C_8$)-4** mixture were mixed with 300 µL cell lysate and incubated at 30 °C and 900 rpm for 2 min. Reaction products were twice extracted with 800 µL of EtOAc + 1 % FA and the organic layer was concentrated in the speed-vac. To obtain compound **18**, 300 µL of compound **4** mixture was incubated with 3 µM TdaE^{Pi} at 30 °C and 900 rpm for 30 min and subsequently twice extracted with 800 µL of EtOAc + 1 % FA. After concentrating in the speed-vac, the volume of all extracts was normalized and samples were analyzed by HPLC-DAD and HR-UPLC-MS.

***Cell-free lysate assay* (Burkholderia plantarii)**
Burkholderia plantarii cultures were harvested after 34 h cultivation in AG medium. Cell lysate was prepared as described above for *Phaeobacter inhibens* (see above). Compound **4** or **($^{13}C_8$)-4** was produced as before (see above). Then, 3 µM TdaE^{Pi} were added to the compound **4** or **($^{13}C_8$)-4** mixture to generate compound **18** or **($^{13}C_8$)-18**, repectively. Subsequently, cleared cell lysate (400 µL) was combined with 200 µL of the **18** or **($^{13}C_8$)-18** mixture and incubated at 30 °C and 900 rpm for 5 min. All the assays were quenched and twice extracted with EtOAc + 1 % FA (700 µL), and the organic phase was concentrated in a speed-vac. After normalizing the sample volume, all assays were analyzed by HPLC-DAD and the peak corresponding to **9** was collected manually. The collected fraction was then extracted with double volume of EtOAc + 1 % FA and the organic layer was again concentrated in a speed-vac. Finally, sample volumes were normalized and conversion of **($^{13}C_8$)-18** into **($^{13}C_7$)-9** was analyzed by HR-UPLC-MS.

***Analysis of TDA production by a wild type and a Δ*tdaE *mutant strain of* Phaeobacter inhibens**
The Δ*tdaE* mutant strain was cultivated as described above for the wild type strain of *Phaeobacter inhibens* (however, in the presence of 50 µg mL^{-1} kanamycin). The wild type culture was harvested after 21 h growth, and the mutant cultures were harvested after 17 h and 21 h cultivation by centrifugation (18 000 g for 20 min). Supernatants were extracted once with double volume of EtOAc + 1 % FA and the organic layers were concentrated to almost complete dryness using a rotavap. Residues were re-dissolved in an equal volume of 85 % acetonitrile and samples were analyzed by HPLC-DAD.

RT-qPCR analysis

Total RNA was extracted from the cells of *P. inhibens* harvested after 12 h and 22 h cultivation and *B. plantarii* harvested after 5 h and 33 h cultivation with TRIzol Reagent (Invitrogen) by following the manufacturer's instruction. The concentration of total RNA was determined using a Biotechnologie NANODROP 1000 Spectrophotometer. Then, reverse transcription to synthesize cDNA was performed according to the protocol of the supplier with the "High Capacity Reverse Transcription Kit" (Thermo Scientific) by using 1 µg total RNA as template. The resulting cDNA was used for RT-qPCR (Absolute qPCRSYBR Green Mix, Thermo Fisher Scientific) in an ABI Prism 7300 (Applied Biosystems). Both, for *P. inhibens* as well as for *B. plantarii*, 16S rRNA was used as a reference gene (for primers see Table 2 below).

Table 2: Primers used for RT-qPCR analysis.

Organism	Gene	Forward (5' to 3')	Reverse (5' to 3')
P. inhibens	16S rRNA	CTAGTGACAGGTGCTGCATG	ATCACGGGCAGTTTCCCTAG
	tdaE	CTGTAAGCTGAGTGACCACGG	GCACATCATCAAATCGCATTTGG
B. plantarii	16S rRNA	CTATAGGGTTGGCCGATGGC	CTGCTGCCTCCCGTAGGA
	tdaE	CACGCTGGTGATGCTCGAC	GATGTCGATCAGCTGCTGCAG

Product purification by preparative HPLC

Samples were filtered before loading to the preparative HPLC column (Eurospher II 100-5 C18, 250 x 20 mm column; KNAUER Wissenschaftliche Geräte GmbH). In each run, the column was pre-equilibrated to a starting condition of 2 % acetonitrile in 10 mM ammonium acetate (AmAc) buffer (pH 4.5) for 10 to 15 min at a flow rate of 8 mL min^{-1}. Then, a linear gradient from 2 % acetonitrile to 50 % acetonitrile within 30 min and a flow rate of 8 mL min^{-1} was applied to elute the desired compound. Chromatogram of separation was monitored at 214 nm and 260 nm with a diode array detector connected to the chromatographic system. Samples were collected after peak appearance (compound **5** ~ 19 min) and the collected fractions subsequently were subjected to lyophilization for solvent removal.

HPLC detection

Sample analysis and compound purification by HPLC was done using an Agilent 1100 chromatographic system (Technologies), equipped with a Semiprep VP NUCLEODUR Gravity SB column (250 × 10 mm ID, 5 µM, Macherey-Nagel) coupled with a UNIVERSAL RP guard column (4 × 3 mm ID, Macherey-Nagel). The column was pre-equilibrated with 10 mM AmAc pH 4.5 (solution A1) and acetonitrile (solution B1) 98:2 (v/v) for analysis of aqueous samples or with ddH_2O + 0.1 % FA (solution A2) and acetonitrile + 0.1 % FA (solution B2) 98:2 (v/v) for extracted compounds and samples were analyzed at a flow rate of 3-3.5 mL min^{-1} using the following gradients: 2 % to 60 % B1 (0-10 min), 60 % B1 (10-12 min), 60 % to 2 % B1 (12-13 min), 2 % B1 (13-18 min) or 2 % to 60 % B2 (0-15 min), 60 % B2 (15-16 min), 60 % to 2 % B2 (16-17 min), 2 % B2 (17-23 min). Considering the strong absorption of the CoA containing compounds at 260 nm and of the tropone derivatives between 300 and 350 nm, absorption changes at 260, 300, and 340 nm were selected to be monitored by the diode array detector connected to the chromatographic system.

Samples from cell lysate assays and metabolites extracted from of *P. inhibens* culture supernatants were analyzed with an analytical EC 150/3 NUCLEODUR Sphinx RP column (150 × 3 mm ID, 5 µM, Macherey-Nagel) coupled with a UNIVERSAL RP guard column (4 × 3 mm ID, Macherey-Nagel). The column was pre-equilibrated with ddH_2O + 0.1 % FA (solution A2) and acetonitrile + 0.1 % FA (solution B2) 98:2 (v/v) for 10 min before measurement at a flow rate of 1 mL min^{-1} using the following gradients: 2 % to 90 % B2 (0-22 min), 90 % to 2 % B2 (22-23 min), 2 % B2 (23-28 min).

LC-HRMS analysis

LC-HRMS analyses were performed using a Waters Acquity UPLC H class system coupled with a diode array detector (Waters). For all analyses, an analytical RP C18 column (Acquity UPLC HSS T3, 100Å, 1.8 μm, 2.1 x 100 mm; Waters) was used, pre-equilibrated with 10 mM AmAc pH 6.8 (solution A1) and acetonitrile (solution B1) 98:2 (v/v) for analysis of aqueous samples or with ddH_2O + 0.1 % FA (solution A2) and acetonitrile + 0.1 % FA (solution B2) 98:2 (v/v). Samples were analyzed at a flow rate of 0.2 mL min^{-1} using the following gradients: 2 % B1/B2 (0-1 min), 2 % to 60 % B1/B2 (1-11 min), 60 % B1/B2 (11-11.5 min), 60 % to 2 % B1/B2 (11.5-11.6 min), 2 % B1/B2 (11.6-14 min). Samples were analyzed in MS positive mode with a capillary voltage of 3.0 kV, 100 °C source temperature, 300 °C desolvation gas temperature and 600 L h^{-1} N_2 desolvation gas flow and a capillary voltage of 1.5 kV, 120 °C source temperature, 300 °C desolvation gas temperature and 500 L h^{-1} N_2 desolvation gas flow for MS negative mode.

General methods for chemical synthesis

Chemicals were purchased from commercial suppliers and used without purification. Solvents for reactions were dried by a solvent purification system (MBraun Inertgassysteme GmbH, Garching, Germany). Solvents for extractions and compound purification were distilled before use. Silica gel chromatography was performed on silica gel (0.04 – 0.06 nm, Acros Organics, Geel, Belgium). Thin layer chromatography (TLC) was performed on POLYGRAM® SIL G/UV254 plates (Macherey-Nagel, Düren, Germany). The staining solution for TLC was molybdophosphoric acid in EtOH (10 g/100 mL).

GC/MS and GC/MS-QTOF analyses

GC/MS analyses were performed on a 7890B GC – 5977A MD system (Agilent, Santa Clara, CA, USA). The GC was fitted with a HP5-MS UI fused silica capillary column (30 m, 0.25 mm i. d., 0.50 μm film). GC parameters were: 1) inlet temperature 250 °C, He flow at 23.3 mL min^{-1}; 2) injection volume 1 – 2 μL; 3) temperature program: starting from 50 °C and hold for 5 min, then increasing with 10 °C/min to 320 °C; 4) 60 s valve time and 5) carrier gas He at 1.2 mL min^{-1}. MS parameters were: 1) source temperature 230 °C; 2) transfer line temperature 250 °C; 3) quadrupole temperature 150 °C and 4) ionization energy 70 eV. Retention indices (*I*) were calculated from retention times of analytes in comparison to the retention times of a homologous series of *n*-alkanes (C_7 – C_{40}).

Chiral GC/MS analyses were performed by using an Agilent Cyclosil-B capillary column (30 m, 0.25 mm i. d., 0.25 μm film). GC program was: starting from 110 °C, increasing with 0.5 °C min^{-1} to 130 °C, followed by an increase with 20 °C min^{-1} to 245 °C, then hold for 5 min. Other settings were the same as mentioned above.

High resolution mass spectroscopy was conducted on a 7890B GC 7200 accurate mass Q-ToF detector system (Agilent). The GC was equipped with a HP5-MS fused silica capillary column (30 m, 0.25 mm i. d., 0.50 μm film). GC parameters were 1) injection volume: 1 μL; 2) split ratio was 5:1 to 100:1, 60 s valve time; 3) carrier gas was He at 1 mL min^{-1}; 4) temperature program was: starting from 50 °C and hold for 5 min, increasing at 10 °C/min to 320 °C. MS parameters were: 1) inlet temperature 250 °C, He flow at 24.6 mL min^{-1}; 2) transfer line temperature 250 °C; 3) ionization energy 70 eV.

NMR spectroscopy

NMR spectra were recorded at 298 K on a Bruker Avance I 400, Avance I 500, Avance III HD 500 or Avance III HD 700 spectrometer (Bruker, Billerica, MA, USA). Spectra were referenced against residual proton signals of deuterated solvents ($CDCl_3$: δ_H = 7.26 ppm, δ_C = 77.16 ppm; C_6D_6: δ_H = 7.16 ppm, δ_H = 128.06 ppm; CD_3CN: δ_H = 1.94 ppm, δ_C = 118.26, 1.32 ppm; D_2O: δ_H = 4.79 ppm)[10]. Signals are given as s = singlet, d = doublet, t = triplet, q = quartet, m = multiplet and br = broad.

Infrared spectroscopy

IR spectra were recorded on a Bruker α infrared spectrometer with a diamond ATR probe head. Peak intensities are given as s (strong), m (medium), w (weak) and br (broad).

Synthetic route to ($^{13}C_8$)-2-phenylacetic acid (($^{13}C_8$)-2)

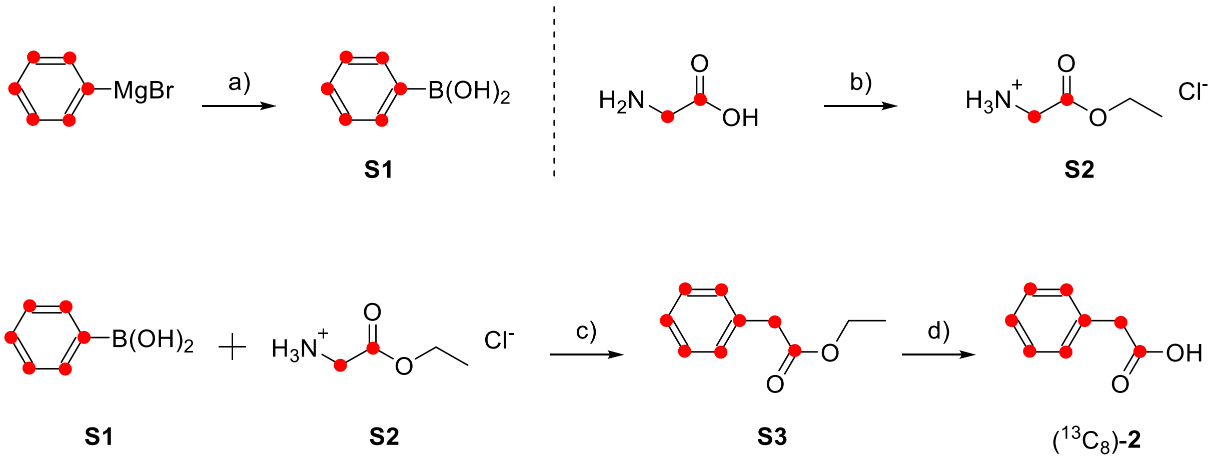

Scheme S1. **Synthesis of ($^{13}C_8$)-2.** Reaction conditions: a) trimethyl borate, THF, –78 °C to room temperature, overnight, 64 %; b) $SOCl_2$, ethanol, reflux, 8 h, 89 %; c) sodium nitrite, NH_4Cl, toluene, H_2O, reflux, 24 h, 71 %; d) NaOH, methanol, room temperature, 2 h, 92 %.

Synthesis of ($^{13}C_6$)phenylboronic acid (S1)

Magnesium turnings (85 mg, 3.52 mmol, 1.1 eq), a crystal of I_2 and a small portion of ($^{13}C_6$)phenyl bromide were added into THF (2 mL). The mixture was heated to initiate the reaction. After that, ($^{13}C_6$)phenyl bromide solution (0.5 g, 3.1 mmol, in 0.5 mL THF) was added dropwise, and the mixture was kept under reflux for 1 h. The reaction mixture was cooled to room temperature and diluted with THF (5 mL). The solution of the resulting Grignard reagent was used for the next step directly.

To a THF (3 mL, –78 °C) solution of trimethyl borate (0.40 g, 3.84 mmol, 1.2 eq) the freshly prepared ($^{13}C_6$)phenylmagnesium bromide solution was added dropwise. The mixture was stirred overnight without further cooling. The reaction was quenched by the addition of aq. HCl (1 M, 5 mL) and stirred at room temperature for 30 min. The product was extracted with Et_2O (3 x 50 mL). The combined extracts were dried with $MgSO_4$ and concentrated under reduced pressure. The residue was washed with hexane to afford **S1** (0.26 g, 0.20 mmol, 64 %) as a colourless powder. EI-MS (70 eV): ($^{13}C_{18}$)phenylboronic acid anhydride, *m/z* (%) = 330 (100), 285 (2), 247 (6), 220 (3), 192 (3), 175 (26), 165 (8), 147 (3), 137 (19), 110 (69), 93(8), 83 (19), 55 (16), 42 (4). GC (HP5-MS): *I* = 2491. ^{1}H NMR (700 MHz, CD_3Cl): δ = 8.23 (dq, $^{1}J_{H,C}$ = 159.0 Hz, $^{3}J_{H,H}$ = 6.5 Hz, 2H), 7.61 (dt, $^{1}J_{H,C}$ = 157.8 Hz, $^{3}J_{H,H}$ = 7.0 Hz, 1H), 7.50 (t, $^{1}J_{H,C}$ = 158.4 Hz, $^{3}J_{H,H}$ = 6.3 Hz, 2H), 1.54 (s, 2H) ppm. ^{13}C NMR (176 MHz, CD_3Cl): δ = 135.77 (t, $^{1}J_{C,C}$ = 52.5 Hz, 2 x ^{13}CH), 132.84 (tdt, $^{1}J_{C,C}$ = 56.9 Hz, $^{2}J_{C,C}$ = 9.0 Hz, $^{3}J_{C,C}$ = 2.5 Hz, ^{13}CH), 128.15 (td, $^{1}J_{C,C}$ = 54.6, $^{2}J_{C,C}$ =10.1 Hz, 2 x ^{13}CH) ppm. ^{11}B NMR (160 MHz, CD_3Cl): δ = 29.54 (d, $^{1}J_{B,C}$ = 88.7 Hz) ppm.

Synthesis of (1,2-$^{13}C_2$)glycine ethyl ester hydrochloride (S2)

($^{13}C_2$)Glycine (0.50 g, 6.50 mmol) was suspended in ethanol (6 mL) and cooled to 0 °C, followed by the dropwise addition of $SOCl_2$ (1.16 g, 0.75 mmol, 1.5 eq). The mixture was heated to reflux and kept under reflux for 8 h. The reaction was cooled to room temperature and the solvent was removed under reduced pressure. The residue was suspended in Et_2O and filtrated and the solid was washed with Et_2O. The solvent was removed under reduced pressure to afford **S2** (0.82 g, 5.80 mmol, 89 %) as a white powder. ^{1}H NMR (500 MHz, D_2O): δ = 4.33 (qd, $^{3}J_{H,H}$ = 7.2 Hz, $^{3}J_{H,C}$ = 3.1 Hz, 2H), 3.94 (dd, $^{1}J_{H,C}$ = 145.9 Hz, $^{2}J_{H,C}$ = 6.4 Hz, 2H), 1.32 (t, $^{3}J_{H,H}$ = 7.2 Hz, 3H) ppm. ^{13}C NMR (126 MHz, D_2O): δ = 168.18

(d, $^1J_{C,C}$ = 62.2 Hz, C_q), 63.32 (dd, $^2J_{C,C}$ = 2.4 Hz, $^3J_{C,C}$ = 1.4 Hz, CH_2), 40.21 (d, $^1J_{C,C}$ = 62.4 Hz, CH_2), 13.18 (d, $^3J_{C,C}$ = 2.2 Hz, CH_3) ppm.

Synthesis of ethyl ($^{13}C_8$)-2-phenylacetate (S3**)**

The reaction was performed according to a published procedure[11]. **S1** (240 mg, 1.88 mmol), **S2** (399 mg, 2.82 mmol, 1.5 eq), sodium nitrite (246 mg, 3.57 mmol, 1.9 eq) and NH_4Cl (402 mg, 7.52 mmol, 4.0 eq) were placed in a flask, toluene (4.5 mL) and H_2O (226 µL) were added. The mixture was heated under reflux for 24 h. The solvents were removed under reduced pressure, and the residue was purified via silica gel chromatography (cyclohexane/ethyl acetate, 20:1, R_f = 0.23) to afford **S3** (230 mg, 1.34 mmol, 71 %) as a colourless oil. EI-MS (70 eV): *m/z* (%) = 172 (15), 127 (2), 112 (1), 98 (100), 83 (2), 70 (16), 55 (3), 42 (7). GC (HP5-MS): *I* = 1246. HRMS (APCI): *m/z* = 173.1177 (calc. for $[^{13}C_8C_2H_{12}O_2+H]^+$: 173.1178). ^1H NMR (500 MHz, C_6D_6): δ = 7.34 – 7.16 (m, 3H), 7.09 – 6.83 (m, 2H), 3.87 (qd, $^3J_{H,H}$ = 7.1 Hz, $^3J_{H,C}$ = 3.2 Hz, 2H), 3.52 – 3.16 (dm, $^1J_{H,C}$ = 129.4 Hz, 2H), 0.88 (t, $^3J_{H,H}$ = 7.1 Hz, 3H) ppm. ^{13}C NMR (126 MHz, C_6D_6): δ = 170.85 (dd, $^1J_{C,C}$ = 57.7 Hz, $^2J_{C,C}$ = 2.2 Hz, $^{13}C_q$), 135.67 – 133.99 (m, $^{13}C_q$), 130.11 – 128.52 (m, 2 x ^{13}CH), 127.68 – 126.56 (m, ^{13}CH), 60.55 (d, $^2J_{C,C}$ = 2.6 Hz, CH_2), 41.57 (ddt, $^1J_{C,C}$ = 57.7 Hz, $^1J_{C,C}$ = 43.6 Hz, $^2J_{C,C}$ = 3.3 Hz, $^{13}CH_2$), 14.15 (d, $^3J_{C,C}$ = 2.1 Hz, CH_3) ppm.

Synthesis of ($^{13}C_8$)-2-phenylacetic acid (($^{13}C_8$)-2**)**

To a methanol (10 mL) solution of **S3** (230 mg, 1.34 mmol) aq. NaOH (2.5 mL, 2 M) was added. The mixture was stirred at room temperature for 2 h. After that, the solution was poured onto aq. HCl (100 mL, 1 M), followed by extraction with Et_2O (3 x 80 mL). The combined extracts were washed with brine and dried with $MgSO_4$. After removal of the solvent under reduced pressure the product ($^{13}C_8$)-**2** (178 mg, 1.23 mmol, 92 %) was obtained as a light yellow powder. EI-MS (70 eV): *m/z* (%) = 144 (27), 126 (1), 98 (100), 83 (3), 70 (22), 55 (7), 46 (20). GC (HP5-MS): *I* = 1250. HRMS (APCI): *m/z* = 145.0862 (calc. for $[^{13}C_8H_9O_2]^+$: 145.0865). ^1H NMR (700 MHz, CD_3Cl): δ = 7.49 – 7.27 (m, 3H), 7.25 – 7.15 (m, 2H), 3.65 (dm, $^1J_{H,C}$ = 129.4 Hz, 2H) ppm. ^{13}C NMR (176 MHz, $CDCl_3$): δ = 177.03 (dd, $^1J_{C,C}$ = 55.7 Hz, $^2J_{C,C}$ = 2.3 Hz, C_q), 134.27 – 132.41 (m, C_q), 129.93 – 128.33 (m, 2 x CH), 127.88 – 127.02 (m, CH), 41.03 (ddtt, $^1J_{C,C}$ = 54.2 Hz, $^1J_{C,C}$ = 43.9 Hz, $^2J_{C,C}$ = 3.4 Hz, $^3J_{C,C}$ = 3.4 Hz) ppm.

Synthetic route to methyl (2*S*,3*S*)-2,3-epoxycycloheptanon-2-carboxylate (2*S*,3*S*)-24

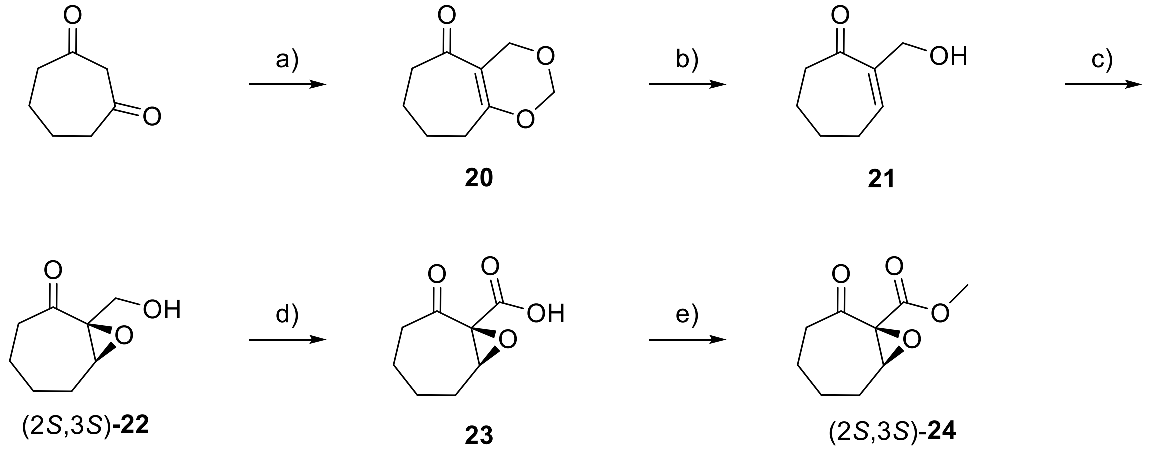

Scheme S2. Synthesis of (2*S*,3*S*)-24. Reaction conditions: a) paraformaldehyde, $BF_3\cdot OEt_2$, CH_2Cl_2, room temperature, 3 h, 35 %; b) DIBAL-H, THF, –78 °C, 2 h, 78 %; c) L-(+)-diisopropyl tartrate, Ti(O*i*Pr)$_4$, 4 Å molecular sieves, *t*-BuOOH, CH_2Cl_2, –17 °C, 20 h, 47 %; d) Jones reagent, acetone, room temperature, 3 h; e) trimethylsilyldiazomethane, Et_2O, 0 °C, 40 min, 24 % over two steps.

Synthesis of 6,7,8,9-tetrahydrocyclohepta[*d*][1,3]dioxin-5(4*H*)-one (20**)**

The reaction was performed as described previously[12]. To a CH_2Cl_2 (35 mL, 0 °C) solution of 1,3-cycloheptanedione (0.63 g, 5.00 mmol) and paraformaldehyde (0.91 g, 30.02 mmol, 6.0 eq) boron trifluoride diethyl etherate (1.85 mL, 15.09 mmol, 3.0 eq) was added dropwise. The mixture was stirred at room temperature for 3 h, and quenched by pouring onto aq. $NaHCO_3$ (50 mL sat. $NaHCO_3$ with 50 mL ice-water). The organic phase was separated, and the aqueous layer was extracted with CH_2Cl_2 (2 x 60 mL). The organic phases were combined, washed with brine and dried with $MgSO_4$. The solvent was removed under vacuum. The product **20** (290 mg, 1.73 mmol, 35 %) was purified via silica gel chromatography (cyclohexane/ethyl acetate, 5:1, R_f = 0.22). EI-MS (70 eV): *m*/*z* (%) = 168 (59), 153 (7), 139 (27), 125 (9), 110 (45), 95 (21), 82 (92), 67 (87), 55 (100), 39 (41). GC (HP5-MS): *I* = 1491. ^{1}H NMR (700 MHz, C_6D_6): δ = 4.54 (s, 2H), 4.52 (t, $^5J_{H,H}$= 1.4 Hz, 2H), 2.31 – 2.24 (m, 2H), 2.08 – 2.01 (m, 2H), 1.27 – 1.24 (m, 4H) ppm. ^{13}C NMR (176 MHz, C_6D_6): δ = 197.49 (C_q), 169.29 (C_q), 114.47 (C_q), 90.98 (CH_2), 64.48 (CH_2), 41.60 (CH_2), 32.11 (CH_2), 23.54 (CH_2), 21.17 (CH_2) ppm.

Synthesis of 2-(hydroxymethyl)cyclohept-2-en-1-one (21**)**

Compound **20** (290 mg, 1.73 mmol) was dissolved in THF (15 mL) and cooled to –78 °C, followed by the dropwise addition of DIBAL-H (2.08 mL, 2.08 mmol, 1.2 eq). The solution was stirred at –78 °C for 2 h, and quenched by the addition of methanol (200 µL). Then sat. Na-K-tartrate solution (3.5 mL) was added to the mixture, and the reaction was stirred at room temperature for 1.5 h. The mixture was extracted with Et_2O (3 x 30 mL). The combined extracts were concentrated under reduced pressure. THF (10 mL) was added to the residue and mixed with aq. HCl (200 µL, 5 % in H_2O). The mixture was stirred for 5 min, followed by the addition of K_2CO_3 (200 mg) and Et_2O (60 mL). The mixture was dried with $MgSO_4$ and concentrated under vacuum. The product **21** (190 mg, 1.36 mmol, 78 %) was obtained via flash chromatography (Et_2O, 100%, R_f = 0.39) as a colourless oil. EI-MS (70 eV): *m*/*z* (%) = 140 (13), 125 (53), 111 (46), 97 (37), 94 (38), 83 (80), 79 (85), 67 (49), 55 (92), 39 (100). GC (HP5-MS): *I* = 1336. ^{1}H NMR (400 MHz, C_6D_6): δ = 6.15 (tt, $^3J_{H,H}$ = 6.2 Hz, $^4J_{H,H}$ =1.2 Hz, 1H), 4.20 (dq, $^3J_{H,H}$ = 6.0 Hz, $^4J_{H,H}$ =1.1 Hz, 2H), 2.29 – 2.23 (m, 2H), 1.75 (qt, $^3J_{H,H}$ = 5.9 Hz, $^5J_{H,H}$ =1.1 Hz, 2H), 1.35 – 1.16 (m, 4H) ppm. ^{13}C NMR (101 MHz, C_6D_6): δ = 204.08 (C_q), 142.54 (CH), 142.39 (C_q), 64.78 (CH_2), 42.96 (CH_2), 27.61 (CH_2), 25.20 (CH_2), 21.37 (CH_2) ppm.

Synthesis of (2*S*,3*S*)-2,3-epoxy-2-(hydroxymethyl)-cycloheptanone ((2*S*,3*S*)-22**)**

L-(+)-Diisopropyl tartrate (478 mg, 2.04 mmol, 1.5 eq), Ti(O*i*Pr)$_4$ (566 mg, 1.99 mmol, 1.46 eq) and 4 Å molecular sieves (570 mg) were added into CH_2Cl_2 (8 mL) and cooled to –20 °C. Compound **21** (190 mg, 1.36 mmol) was added to the reaction mixture dropwise and stirring was continued at –20 °C for 0.5 h. The mixture was cooled to –30 °C, followed by the dropwise addition of *t*-BuOOH (0.75 mL, 5.5 M in decane, 4.15 mmol, 3.05 eq). The mixture was slowly warmed to –17 °C and stirred at this temperature for 20 h. The reaction was quenched by the addition of $FeSO_4 \cdot 7\,H_2O$ (1.15 g) and aq. tartaric acid (8 mL, 10 % in H_2O) at –17 °C, followed by the addition of H_2O (60 mL). The product was extracted with Et_2O (3 x 80 mL). The combined extracts were dried with $MgSO_4$ and concentrated under reduced pressure. Compound **22** (100 mg, 0.64 mmol, 47 %) was obtained as a colourless oil by purification via silica gel chromatography (pentane/Et_2O, 1:5, R_f = 0.45). Optical rotation: $[\alpha]_D^{20}$ = –21.2 (*c* 0.26, CH_2Cl_2). Enantiomeric excess (chiral GC): 94 % *ee*. EI-MS (70 eV): *m*/*z* (%) = 138 (9), 127 (19), 100 (20), 97 (43), 81 (25), 69 (70), 55 (100), 41 (95). GC (HP5-MS): *I* = 1318. HRMS (ESI): *m*/*z* = 157.0861 (calc. for $[C_8H_{12}O_3+H]^+$: 157.0859). IR (diamond ATR): $\tilde{\nu}$ = 3451 (w), 2931 (m), 2860 (w), 1694 (s), 1450 (w), 1423 (w), 1360 (w), 1261 (w), 1224 (w), 1202 (w), 1168 (w), 1095 (w), 1081 (w), 1058 (m), 1012 (m), 941 (w), 905 (w), 887 (w), 865 (w), 802 (w), 750 (w), 646 (w), 587 (w), 564 (w), 548 (w), 496 (w), 451 (w), 412 (w). ^{1}H NMR (500 MHz, C_6D_6): δ = 3.78 (d, $^3J_{H,H}$ = 6.8 Hz, 2H), 2.87 (d, $^3J_{H,H}$ = 5.8 Hz, 1H), 2.47 (ddd, $^3J_{H,H}$ = 13.4 Hz, $^2J_{H,H}$ = 11.0 Hz, $^3J_{H,H}$ = 3.8 Hz, 1H), 2.08 (dddd, $^2J_{H,H}$ = 11.0 Hz, $^3J_{H,H}$ = 5.7 Hz, $^3J_{H,H}$ =2.7 Hz, $^5J_{H,H}$ =1.3 Hz, 1H), 1.76 – 1.68 (m, 2H), 1.42 – 1.31 (m, 1H), 1.30 –

1.21 (m, 1H), 1.12 – 1.04 (m, 2H), 1.01 – 0.85 (m, 1H), 0.65 (tdt, $^3J_{H,H}$ = 13.6, $^2J_{H,H}$ =12.7, $^3J_{H,H}$ = 2.4 Hz, 1H) ppm. ^{13}C NMR (126 MHz, C_6D_6): δ = 211.01 (C_q), 66.58 (C_q), 62.82 (CH_2), 58.14 (CH), 40.84 (CH_2), 26.90 (CH_2), 23.96 (CH_2), 23.09 (CH_2) ppm.

Synthesis of methyl (2*S*,3*S*)-2,3-epoxycycloheptanon-2-carboxylate ((2*S*,3*S*)-24)

Epoxy-alcohol **22** (32 mg, 0.20 mmol) was dissolved in acetone (3 mL) and the solution was cooled to 0 °C, followed by the addition of Jones reagent (240 μL, 2.5 M in aq. H_2SO_4, 0.60 mmol, 3.0 eq). The mixture was stirred at room temperature for 3 h. Then *i*PrOH (0.5 mL) was added. The mixture was stirred for 15 min and concentrated under vacuum, followed by the addition of H_2O (20 mL). Et_2O (3 x 20 mL) was used to extract the product. The combined extractions were dried with $MgSO_4$ and concentrated under reduced pressure to afford the crude product **23**, which was used for the next step directly.

The crude **23** was dissolved in Et_2O (1 mL) and the resulting solution was cooled to 0 °C. Trimethylsilyldiazomethane (1 mL, 0.6 M in hexane, 0.6 mmol) was added dropwise to the mixture and the reaction was stirred at 0 °C for 40 min. Acetic acid (40 μL) was added to the mixture and stirring was continued for 5 min. The solvent was removed under reduced pressure. Purification via silica gel chromatography (pentane/Et_2O, 1:1, R_f = 0.43) provided compound (2*S*,3*S*)-**24** (9 mg, 0.05 mmol, 24 %) as a colourless oil. Optical rotation: $[\alpha]_D^{20}$ = –71.3 (*c* 0.30, CH_2Cl_2). Enantiomeric excess (chiral GC): 95 % *ee*. EI-MS (70 eV): *m*/*z* (%) = 184 (1), 153 (1), 125 (53), 111(2), 97 (29), 79 (23), 69 (87), 55 (100), 41 (80). GC (HP5-MS): *I* = 1435. HRMS (ESI): *m*/*z* = 185.0809 (calc. for $[C_9H_{12}O_4+H]^+$: 185.0809). IR (diamond ATR): $\tilde{\nu}$ = 2936 (w), 2861 (w), 1748 (s), 1703 (s), 1438 (m), 1354 (w), 1334 (w), 1296 (m), 1257 (m), 1224 (w), 1195 (w), 1175 (w), 1135 (w), 1102 (w), 1069 (m), 1041 (w), 1014 (m), 938 (w), 903 (w), 883 (w), 842 (w), 822 (w), 782 (w), 767 (w), 727 (w), 703 (w), 604 (w), 569 (w), 463 (w), 424 (w). ^{1}H NMR (500 MHz, C_6D_6): δ = 3.45 (dd, $^3J_{H,H}$ = 6.0 Hz, $^3J_{H,H}$ =1.1 Hz, 1H), 3.30 (s, 3H), 2.41 (ddd, $^2J_{H,H}$ = 12.4 Hz, $^3J_{H,H}$ = 11.2 Hz, $^3J_{H,H}$ = 3.8 Hz, 1H), 2.14 (dddd, $^2J_{H,H}$ = 11.2 Hz, $^3J_{H,H}$ = 6.3 Hz, $^3J_{H,H}$ = 3.0 Hz, $^5J_{H,H}$ = 1.2 Hz, 1H), 1.59 – 1.48 (m, 1H), 1.28 – 1.06 (m, 3H), 1.01 – 0.91 (m, 1H), 0.76 – 0.62 (m, 1H) ppm. ^{13}C NMR (126 MHz, C_6D_6): δ = 204.83 (C_q), 167.09 (C_q), 65.27 (C_q), 59.36 (CH), 52.30 (CH_3), 40.56 (CH_2), 26.85 (CH_2), 24.27 (CH_2), 22.46 (CH_2) ppm.

Synthetic route to methyl 2,3-epoxycycloheptanon-2-carboxylate ((*rac*)-24)

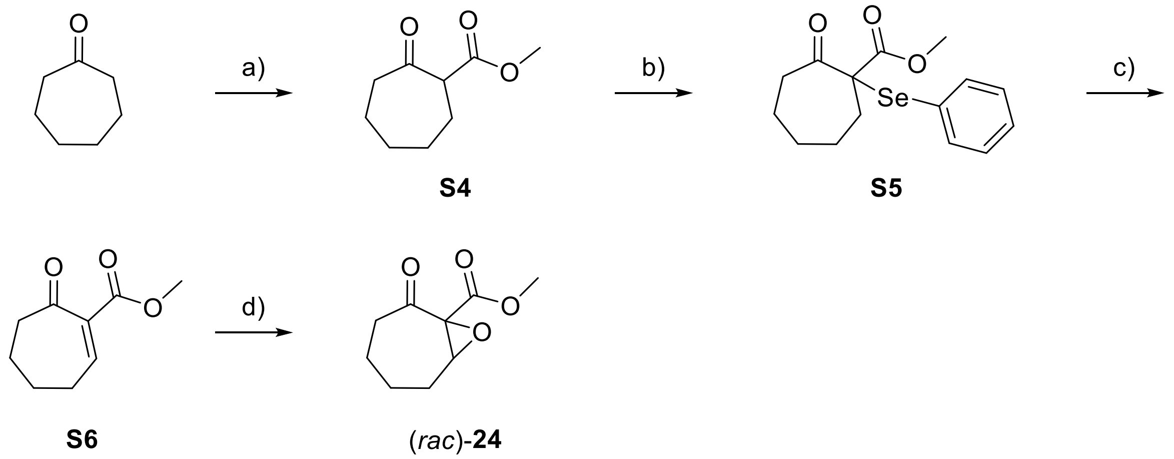

Scheme S3. Synthesis of (*rac*)-24. Reaction conditions: a) NaH, dimethyl carbonate, toluene, reflux, 6 h, 83 %; b) NaH, PhSeBr, THF, 0 °C to room temperature, 50 min, 95 %; c) H_2O_2, CH_2Cl_2, 25 °C, 1 h, 83 %; d) *m*CPBA, KOH, CH_2Cl_2, room temperature, 50 min, 79 %.

Synthesis of methyl 2-oxocycloheptane-1-carboxylate (S4**)**

Cycloheptanone (2.24 g, 20 mmol), NaH (3.20 g, 60 % in mineral oil, 80 mmol, 4.0 eq) and dimethyl carbonate were added into toluene (250 mL). The mixture was heated to reflux and kept under reflux for 6 h. The mixture was cooled to room temperature, followed by acidification with HCl (300 mL, 0.5 M in H_2O). The water phase was separated and extracted with Et_2O (2 x 100 mL). The organic layers were combined, washed with brine, dried with $MgSO_4$ and concentrated under vacuum. The product **S4** (2.83 g, 16.60 mmol, 83 %) was purified via flash chromatography (cyclohexane/ethyl acetate, 5:1, R_f = 0.33) and obtained as colourless oil. EI-MS (70 eV): *m/z* (%) = 170 (19), 155 (1), 142 (28), 138 (41), 127 (8), 113 (30), 110 (33), 101 (6), 97 (9), 87 (30), 82 (40), 74 (24), 67 (22), 55 (100), 41 (43). GC (HP5-MS): *I* = 1353. NMR spectra showed the keto and enol form of **24** (keto:enol = 5:1) in C_6D_6 [13]. ^{1}H NMR (500 MHz, C_6D_6): keto form: δ = 3.36 (s, 3H), 3.31 (dd, $^3J_{H,H}$ = 10.2 Hz, $^3J_{H,H}$ = 4.1 Hz, 1H), 2.32 – 2.20 (m, 2H), 1.79 – 1.60 (m, 2H), 1.51 – 1.24 (m, 4H), 1.01 – 0.87 (m, 2H) ppm. ^{13}C NMR (126 MHz, C_6D_6): keto form: δ = 207.12 (C_q), 170.89 (C_q), 58.87 (CH), 51.60 (CH_3), 43.04 (CH_2), 29.65 (CH_2), 28.10 (CH_2), 27.73 (CH_2), 24.34 (CH_2); enol form: δ = 180.41 (C_q), 173.67 (C_q), 101.59 (C_q), 51.20 (CH_3), 35.53 (CH_2), 32.15 (CH_2), 24.82 (CH_2), 24.68 (CH_2) ppm; one peak was overlapped with a peak originating from the keto from.

Synthesis of methyl 2-oxo-1-(phenylselanyl)cycloheptane-1-carboxylate (S5**)**

NaH (423 mg, 60 % in mineral oil, 10.6 mmol, 1.2 eq) was suspended in THF (25 mL) and the suspension was cooled to 0 °C, followed by the dropwise addition of **S4** (1.50 g, 8.81 mmol). The mixture was stirred at 0 °C for 0.5 h, then phenylselenyl bromide (2.49 g, 10.57 mmol, 1.2 eq, in 5 mL THF) was added dropwise to the reaction mixture. The mixture was stirred at 0 °C for 20 min and at room temperature for 0.5 h. The reaction was quenched by pouring onto aq. $NaHCO_3$ (50 mL sat. $NaHCO_3$ with 100 mL ice-water). The product was extracted with Et_2O (3 x 80 mL). The organic layers were combined, washed with brine, dried with $MgSO_4$ and concentrated under vacuum. The product **S5** (2.73 g, 8.39 mmol, 95 %) was obtained as a colourless oil by purification via flash chromatography (cyclohexane/ethyl acetate, 5:1, R_f = 0.38). EI-MS (70 eV): *m/z* (%) = 326 (51), 314 (8), 294 (5), 266 (7), 250 (3), 237 (11), 211 (5), 195 (6), 183 (16), 169 (6), 157 (80), 141 (40), 129 (4), 109 (53), 91 (13), 81 (100), 67 (16), 55 (34), 41 (25). GC (HP5-MS): *I* = 2248. HRMS (ESI): *m/z* = 327.0495 (calc. for $[C_{15}H_{18}O_3Se+H]^+$: 327.0494). ^{1}H NMR (500 MHz, C_6D_6): δ = 7.64 – 7.57 (m, 2H), 7.07 – 6.97 (m, 3H), 3.28 (s, 3H), 2.43 – 2.26 (m, 2H), 2.06 – 1.84 (m, 2H), 1.52 – 1.40 (m, 2H), 1.38 – 1.27 (m, 2H), 1.18 – 1.08 (m, 2H) ppm. ^{13}C NMR (126 MHz, C_6D_6): δ = 204.62 (C_q), 170.63 (C_q), 138.90 (2 x CH), 129.55 (CH), 128.96 (2 x CH), 66.58 (C_q), 52.09 (CH_3), 41.32 (CH_2), 33.15 (CH_2), 29.57 (CH_2), 26.02 (CH_2), 25.13 (CH_2) ppm.

Synthesis of methyl 7-oxocyclohept-1-ene-1-carboxylate (S6**)**

To a CH_2Cl_2 (35 mL, 0 °C) solution of **S5** (2.73 g, 8.39 mmol) H_2O_2 (3.6 mL 35 % aq. H_2O_2 with 4.7 mL H_2O) was slowly added dropwise. The mixture was stirred at 25 °C for 1 h, then quenched by pouring onto aq. $NaHCO_3$ (50 mL sat. $NaHCO_3$ with 100 mL ice-water). The mixture was extracted with CH_2Cl_2 (3 x 50 mL). The extracts were combined and washed with brine. The solvent was dried with $MgSO_4$ and removed under reduced pressure. Purification via silica gel chromatography (cyclohexane/ethyl acetate, 2:1, R_f = 0.35) provided compound **S6** (1.17 g, 6.96 mmol, 83 %) as a colourless oil. EI-MS (70 eV): *m/z* (%) = 168 (4), 154 (3), 140 (75), 125 (7), 108 (100), 98 (12), 80 (59), 68 (39), 59 (38), 53 (80), 39 (77). GC (HP5-MS): *I* = 1445. ^{1}H NMR (500 MHz, C_6D_6): δ = 7.00 (t, $^3J_{H,H}$ = 5.8 Hz, 1H), 3.41 (s, 3H), 2.47 – 2.24 (m, 2H), 1.71 – 1.62 (m, 2H), 1.31 – 1.18 (m, 2H), 1.13 – 1.04 (m, 2H) ppm. ^{13}C NMR (126 MHz, C_6D_6): δ = 200.73 (C_q), 165.88 (C_q), 147.05 (CH), 137.60 (C_q), 51.67 (CH_3), 43.74 (CH_2), 28.65 (CH_2), 24.37 (CH_2), 22.67 (CH_2) ppm.

Synthesis of methyl 2,3-epoxycycloheptanon-2-carboxylate ((*rac*)-24)

*m*CPBA (1.60 g, 7.12 mmol, 2.0 eq) was dissolved in CH_2Cl_2 (80 mL) and the solution was cooled to 0 °C, followed by the addition of KOH (0.60 g, 10.68 mmol, 3.0 eq)[14]. After stirring at room temperature for 1 h, **S6** (0.60 g, 3.56 mmol, in 5 mL CH_2Cl_2) was added to the mixture dropwise. The reaction mixture was then stirred for another 50 min. The mixture was filtrated, and the filtrate was concentrated under vacuum. The residue was purified via silica gel chromatography to provide (*rac*)-**24** (0.52 g, 2.82 mmol, 79 %) as a colourless oil. Spectroscopic data were identical to those of (2*S*,3*S*)-**24**.

Derivatization of enzymatically generated 18 for verification of its structure and determination of its absolute configuration

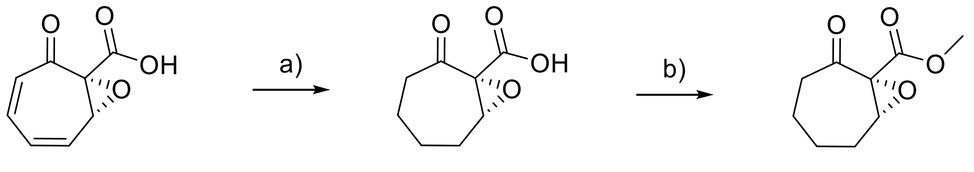

18 **25** (2*R*,3*R*)-**24**

Scheme S4. Derivatization of enzymatically obtained **18**. Reaction conditions: a) Pd/C, methanol, room temperature, 30 min; b) trimethylsilyldiazomethane, benzene, room temperature, 30 min.

The enzymatic reaction mixture (18 mL) obtained with TdaE^{Pi} was adjusted to pH 3.0 by addition of aq. HCl (1M), then the product was extracted with ethyl acetate (2 x 40 mL). The extracts were combined, washed with brine and carefully concentrated under reduced pressure (25 °C, 80 mbar) to a volume of 100 μL. After transfer to a reaction vessel, methanol (1 mL) and Pd/C (4.0 mg, 10 % Pd) were added. The flask was vacuumed shortly and flushed with H_2 using a balloon of hydrogen (pressure slightly higher than 1 bar). This process was repeated three times to exchange the atmosphere to H_2 totally. The mixture was stirred at room temperature for 30 min, the catalyst was filtered off and washed with methanol (2 x 1 mL). The filtrate was concentrated carefully to 300 μL.

The mixture was diluted with benzene (1.2 mL), followed by the addition of trimethylsilyldiazomethane (100 μL, 0.6 M in hexane). The mixture was stirred at room temperature for 30 min, and then quenched by the addition of formic acid (600 μL, 1 % in H_2O). The mixture was centrifuged to separate the organic and the aqueous layers. The organic layer was recovered, washed with brine (400 μL) and concentrated to 100 μL for GC/MS analysis.

Supporting Figures and Tables

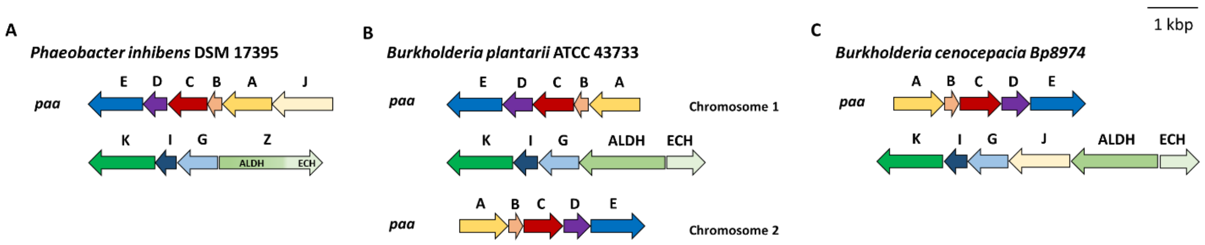

Figure S1: Graphical representation of the *paa* catabolic gene clusters identified in *Phaeobacter inhibens* DSM 17395, in *Burkholderia plantarii* ATCC 43733, as well as in *Burkholderia cenocepacia* Bp8974.

Table S1: Summary of the gene cluster analysis (*paa* and *tda*) of *Phaeobacter inhibens* DSM17395. Using blastp and by subsequently analyzing the gene environment of the corresponding nucleotide sequences, one *tda* gene cluster and two gene clusters related to *paa* catabolism could be found. Encoded proteins are listed below (annotation taken from NCBI directly or modified based on available experimental data).

**TDA gene cluster**		**Annotation**	**AA**
**PaaZ-2**	WP_014881711.1	phenylacetic acid degradation bifunctional protein PaaZ	710
**TdaF**	WP_014881712.1	flavoprotein	189
**TdaE**	WP_014881725.1	acyl-CoA/acyl-ACP dehydrogenase	397
**TdaD**	WP_014881726.1	acyl-CoA thioesterase	138
**TdaC**	WP_014881727.1	hypothetical protein (prephenate dehydratase)	200
**TdaB**	WP_014881728.1	glutathione S-transferase family protein	232
**TdaA**	WP_014881729.1	LysR family transcriptional regulator	296
**Paa gene cluster 1**			
**PaaK**	WP_014880890.1	phenylacetate-CoA ligase	435
**PaaI**	WP_014880891.1	hydroxyphenylacetyl-CoA thioesterase PaaI	141
**PaaG**	WP_014880892.1	2-(1,2-epoxy-1,2-dihydrophenyl)acetyl-CoA isomerase	261
**PaaZ**	WP_014880893.1	phenylacetic acid degradation bifunctional protein PaaZ	676
**Paa gene cluster 2**			
**PaaE**	WP_014879081.1	1,2-phenylacetyl-CoA epoxidase subunit	357
**PaaD**	WP_014879082.1	maturation (?) of 1,2-phenylacetyl-CoA epoxidase	157
**PaaC**	WP_014879083.1	1,2-phenylacetyl-CoA epoxidase subunit	257
**PaaB**	WP_014879084.1	1,2-phenylacetyl-CoA epoxidase subunit	94
**PaaA**	WP_014879085.1	1,2-phenylacetyl-CoA epoxidase subunit	325
**PaaJ**	WP_014879086.1	3-oxoadipyl-CoA thiolase	400
**PatB**	WP_014878874.1	pyridoxal phosphate-dependent aminotransferase	390

Table S2: Summary of the gene cluster analysis (*paa* and *tda*) of *Burkholderia plantarii* ATCC 43733. Using blastp and by subsequently analyzing the gene environment of the corresponding nucleotide sequences, one "*tda* gene cluster" and three gene clusters related to *paa* catabolism (two one chromosome 1 and one on chromosome 2) could be found. Encoded proteins are listed below (annotation taken from NCBI directly or modified based on available experimental data).

**TDA gene cluster**			AA
**DPHS**	WP_052498254.1	3-deoxy-7-phosphoheptulonate synthase	360
**TdaD**	WP_042624078.1	acyl-CoA thioesterase	136
**TdaE**	WP_042624079.1	acyl-CoA/acyl-ACP dehydrogenase	387
**TdaC**	WP_063891179.1	prephenate dehydratase	385
**?**	WP_042624080.1	hypothetical protein	280
**SK**	WP_052498359.1	shikimate kinase	172
**ACT**	WP_042624082.1	acetyltransferase	238
**O**	WP_042624083.1	LLM class flavin-dependent oxidoreductase	457
**TR**	WP_042624084.1	MHS family MFS transporter	449
**DC**	WP_052498255.1	carboxymuconolactone decarboxylase family protein	202
**Paa gene clusters chromosome 1**			
**PaaK**	WP_042623757.1	phenylacetate--CoA ligase	431
**PaaI**	WP_042623758.1	hydroxyphenylacetyl-CoA thioesterase PaaI	158
**PaaG**	WP_042623759.1	2-(1,2-epoxy-1,2-dihydrophenyl)acetyl-CoA isomerase	263
**ALDH**	WP_042623760.1	aldehyde dehydrogenase	564
**ECH**	WP_042623761.1	enoyl-CoA hydratase	258
**PaaE**	WP_042623615.1	1,2-phenylacetyl-CoA epoxidase subunit	362
**PaaD**	WP_042623616.1	maturation (?) of 1,2-phenylacetyl-CoA epoxidase	195
**PaaC**	WP_042623617.1	1,2-phenylacetyl-CoA epoxidase subunit	268
**PaaB**	WP_042623618.1	1,2-phenylacetyl-CoA epoxidase subunit	94
**PaaA**	WP_042626363.1	1,2-phenylacetyl-CoA epoxidase subunit	333
**Paa gene cluster chromosome 2**			
**DC**	WP_042627921.1	carboxymuconolactone decarboxylase family protein	144
**CoA L**	WP_042627922.1	phenylacetate-CoA ligase	581
**PaaA**	WP_042627923.1	1,2-phenylacetyl-CoA epoxidase subunit	318
**PaaB**	WP_042627924.1	1,2-phenylacetyl-CoA epoxidase subunit	93
**PaaC**	WP_080937406.1	1,2-phenylacetyl-CoA epoxidase subunit	256
**PaaD**	WP_042627926.1	maturation (?) of 1,2-phenylacetyl-CoA epoxidase	171
**PaaE**	WP_042627927.1	1,2-phenylacetyl-CoA epoxidase subunit	353

Table S3: Summary of the gene cluster analysis (*paa* and *tda*) of *Burkholderia cenocepacia* Bp8974. Using blastp and by subsequently analyzing the gene environment of the corresponding nucleotide sequences, one "*tda* gene cluster" and two gene clusters related to *paa* catabolism could be found. Encoded proteins are listed below (annotation taken from NCBI directly or modified based on available experimental data).

**TDA gene cluster**			AA
**?**	WP_089488454.1	hypothetical protein	337
**TdaE**	WP_124630373.1	acyl-CoA dehydrogenase family protein	380
**CoA L**	WP_050012680.1	CoA ester lyase	300
**PLP-synthase**	WP_050012679.1	pyridoxal 5'-phosphate synthase	217
**PatB?**	WP_080334194.1	cystathionine beta-lyase	412
**TdaB?**	WP_050012677.1	glutathione S-transferase	214
**GAT**	WP_124630374.1	glutamine amidotransferase	260
**AICP**	WP_185641082.1	autoinducer binding domain-containing protein	223
**?**	WP_050012700.1	hypothetical protein	143
**EP 1**	WP_089488453.1	DHA2 family efflux MFS transporter permease subunit	512
**EP 2**	WP_124630375.1	efflux RND transporter periplasmic adaptor subunit	454
**EP 3**	WP_124630376.1	efflux transporter outer membrane subunit	499
**HTH R**	WP_050012673.1	helix-turn-helix transcriptional regulator	278
**GCL**	WP_089488653.1	glutamate--cysteine ligase	455
**DHPS**	WP_124556479.1	3-deoxy-7-phosphoheptulonate synthase	370
**TdaD**	WP_050012672.1	acyl-CoA thioesterase	136
**Paa gene clusters**			
**PaaA**	WP_050018275.1	1,2-phenylacetyl-CoA epoxidase subunit	332
**PaaB**	WP_006482881.1	1,2-phenylacetyl-CoA epoxidase subunit	94
**PaaC**	WP_050018276.1	phenylacetate-CoA oxygenase subunit	267
**PaaD**	WP_050018277.1	phenylacetate-CoA oxygenase subunit	184
**PaaE**	WP_124631740.1	phenylacetate-CoA oxygenase/reductase subunit	362
**PaaK**	WP_124631223.1	phenylacetate-CoA ligase	432
**PaaI**	WP_089488134.1	hydroxyphenylacetyl-CoA thioesterase PaaI	150
**PaaG**	WP_124631224.1	2-(1,2-epoxy-1,2-dihydrophenyl)acetyl-CoA isomerase	263
**PaaJ**	WP_050013043.1	3-oxoadipyl-CoA thiolase	400
**ALDH**	WP_124631225.1	aldehyde dehydrogenase	568
**ECH**	WP_050013041.1	enoyl-CoA hydratase	258

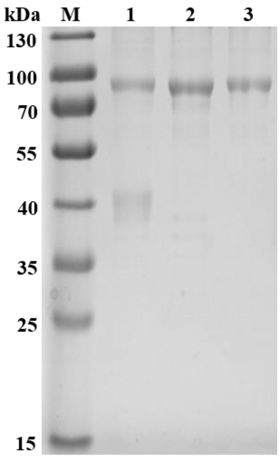

Figure S2: SDS-PAGE analysis of the different fractions collected during affinity purification or size exclusion chromatography of MBP-tagged TdaE^{Pi} (86 kDa). M, PageRuler Prestained protein ladder (Thermo Scientific). **1**, elution fraction from affinity chromatography; **2**, major peak fraction and **3**, "shoulder" fraction of the analytical gel filtration.

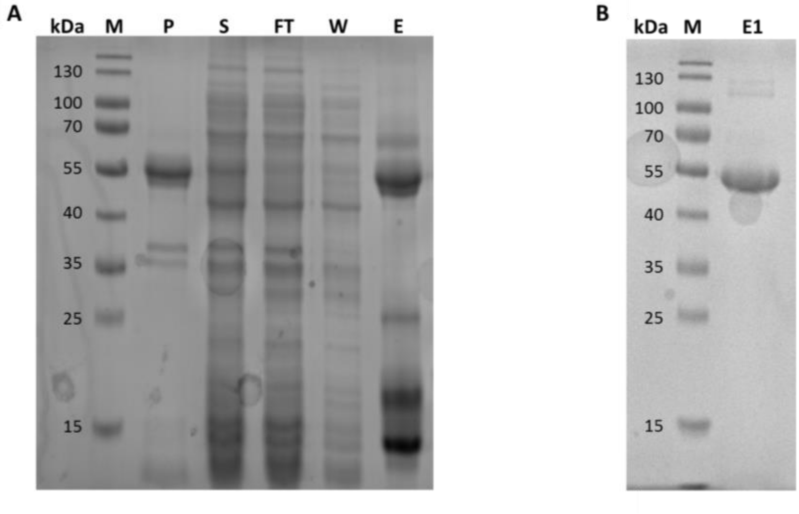

Figure S3: SDS-PAGE analysis of the different fractions collected in the course of the affinity purification (A**) and after a second purification step via size exclusion chromatography (**B**) of GB1-tagged TdaE*^{Bp}* (52 kDa)**. **A**, In lane 1 the marker (**M**; PageRuler Prestained protein ladder, Thermo Scientific), in lane 2 the cell pellet after lysis (**P**), in lane 3 the cleared cell lysate (**S**), in lane 4 the column flow through (**FT**), in lane 5 the wash fraction (**W**) and in lane 6 the elution fraction (**E**) are shown. **B**, In lane 1 the marker (**M**; PageRuler Prestained protein ladder, Thermo Scientific) and in lane 2 the major peak fraction (**E1**) are shown.

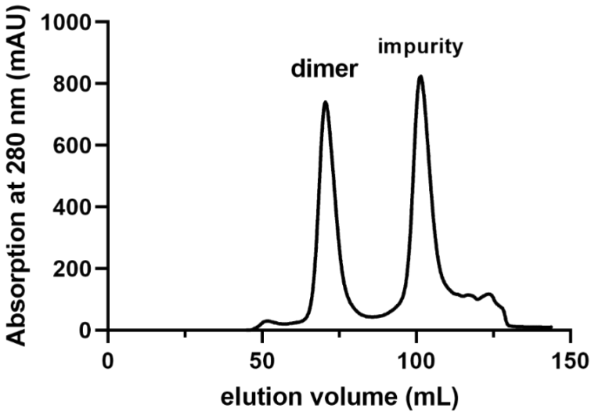

Figure S4: Gelfiltration profile of GB1-tagged TdaE^{Bp}. The major peak at an elution volume of about 70 mL indicates that TdaE^{Bp} forms a stable dimer in solution (monomer: ~52 kDa). The second peak around 100 mL corresponds to the impurities co-purified upon affinity chromatography.

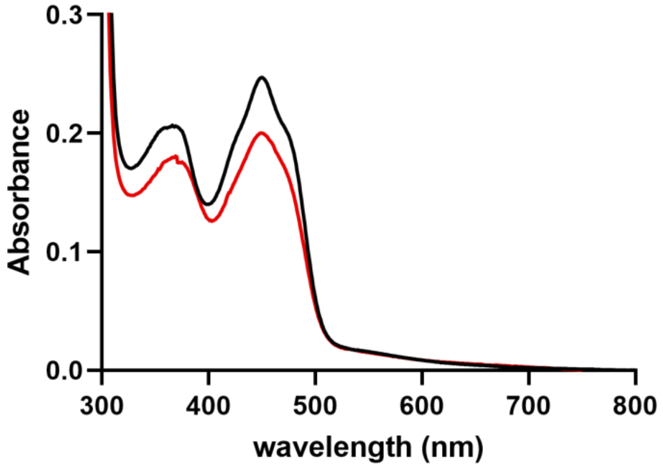

Figure S5: UV-visible absorption spectra of native (*black*) and denatured (*red*) TdaE^{Bp}. The spectrum of native TdaE^{Bp} was recorded in 50 mM Tris, 300 mM NaCl, 30 mM imidazole, pH 7.4 between 300 and 800 nm, just prior the addition of 0.5 % SDS to obtain the absorption spectrum of the cofactor released from TdaE^{Bp}.

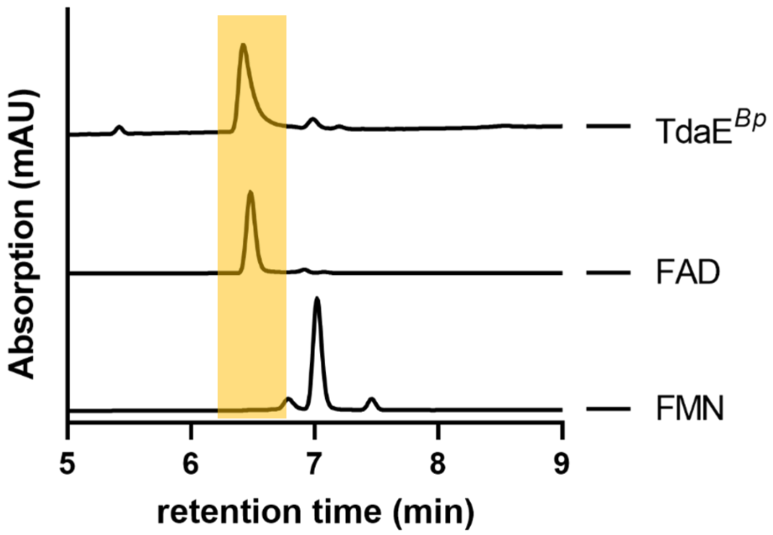

Figure S6: RP-HPLC analysis (350 nm) of the flavin cofactor isolated from TdaE^{Bp}. Based on the retention time of the flavin species (compared to an FMN and an FAD-standard, *lower two lanes*) released from TdaE^{Bp} upon denaturation with EtOAc:FA (9:1), its cofactor was identified as FAD.

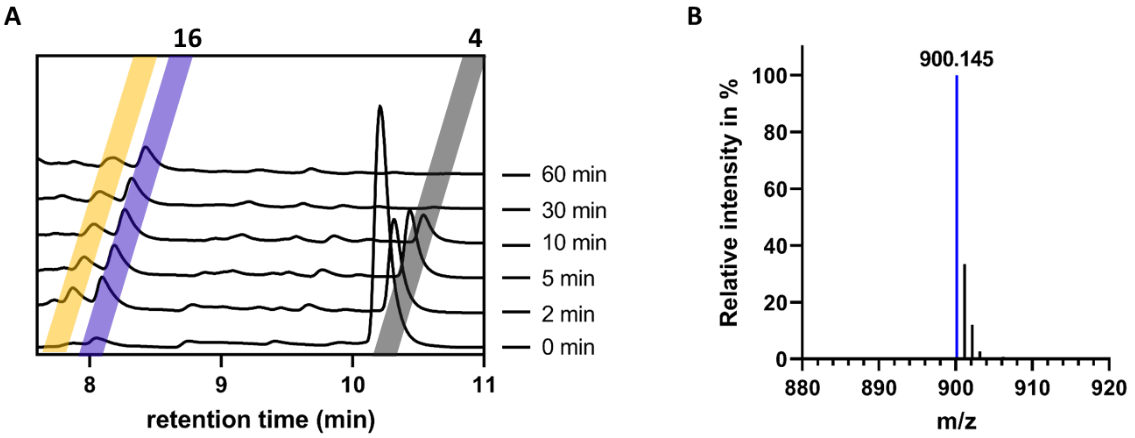

Figure S7: TdaE^{Pi}-catalyzed turnover of compound 4 **into compound** 16**. A,** RP-HPLC analysis (chromatogram at 260 nm) of samples withdrawn from enzyme assays with TdaE^{Pi} after 0, 2, 5, 10, 30 and 60 min. Peaks corresponding to the substrate **4** and the reaction intermediate **16** are indicated by *grey* and *blue* lines, respectively. The *yellow* line highlights the peaks corresponding to the FAD in the assay. **B,** LC-HRMS analysis of compound **16** in positive ion mode (calculated MH^+, 900.144; measured MH^+, 900.145)**.**

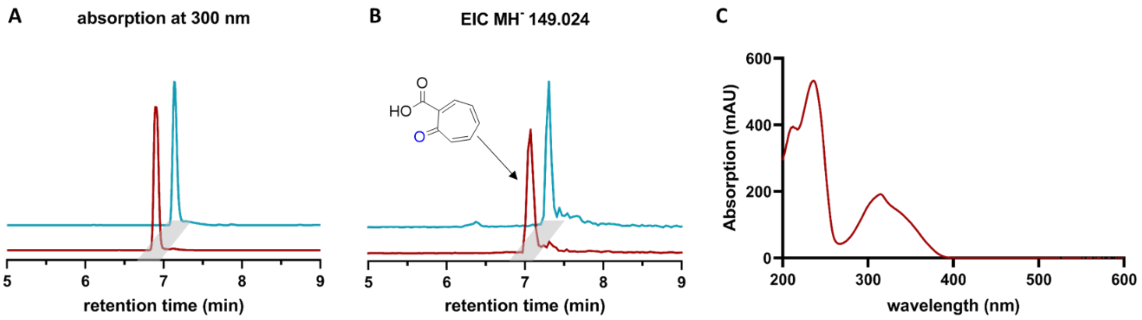

Figure S8: Identification of the hydrolysis product of compound 16 **using thioesterase PaaY.** Traces with the same color derive from the same assay. Standard of tropone-2-carboxylic acid (**17**) was used as control (*red* line), compound **16** was incubated with thioesterase PaaY (*cyan* line). **A,** RP-HPLC analysis at 300 nm showing the identical retention time of the PaaY hydrolysis product of compound **16** and tropone-2-carboxylic acid standard. **B,** Shown are the extracted ion chromatograms (EICs) for MH^- 149.024 of tropone-2-carboxylic acid standard (**17**) and the PaaY hydrolysis product of compound **16**. **C,** UV-visible absorption spectrum of compound **17**. It shows the maximum absorption at around 236 nm and 314 nm.

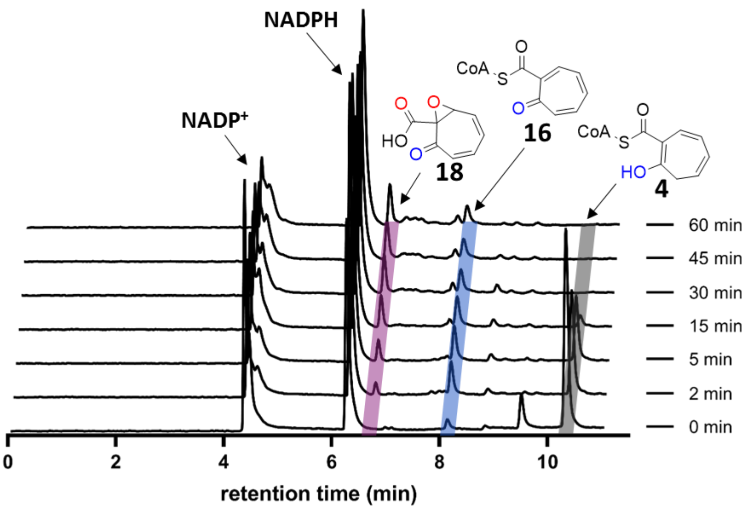

Figure S9: Uncropped RP-HPLC chromatograms (at 300 nm) corresponding to the time course graphs shown in the Figure 3B. The “additional” peaks at around 4.3 min and 6.3 min correspond to $NADP^+$ and NADPH, respectively.

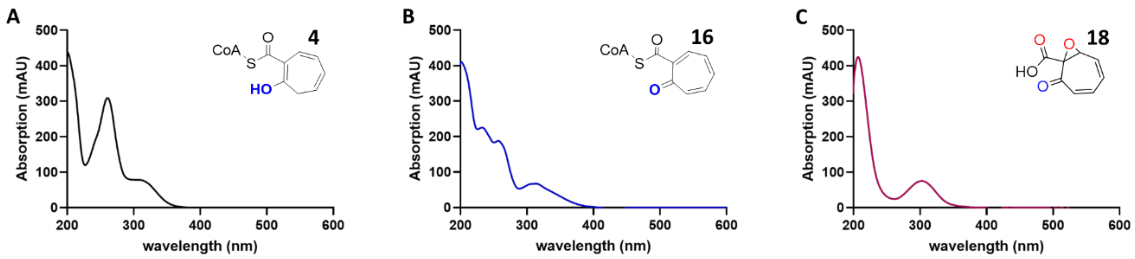

Figure S10: UV-visible absorption spectra of compounds 4 **(*black*),** 16 **(*blue*) and** 18 **(*purple*) recorded upon HPLC-analysis of the enzyme assays with TdaE.** The absorption maximum at 260 nm in the UV-vis spectrum of compound **4** is caused by the CoA-moiety.

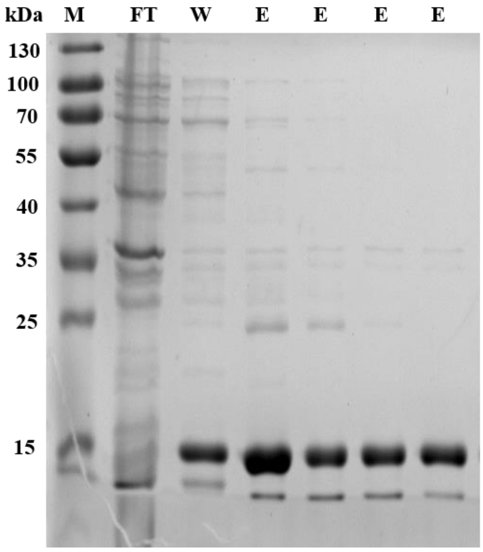

Figure S11: SDS-PAGE analysis of the different fractions in the course of the affinity purification of His$_6$-tagged TdaD^{Pi} (18 kDa). M, PageRuler Prestained protein ladder (Thermo Scientific); **FT**, the column flow through; **W**, the wash fraction; **E**, the elution fractions.

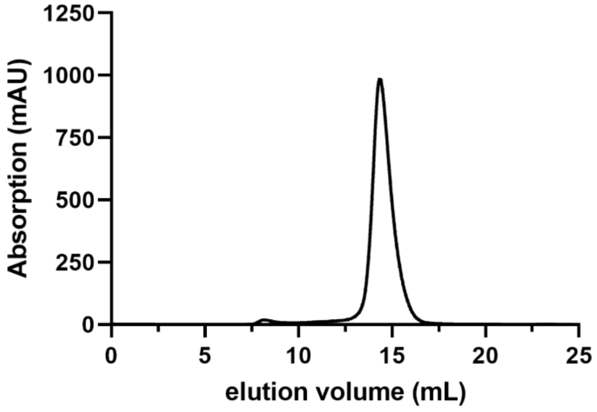

Figure S12: Analytical size-exclusion chromatography profile of polyhistidine-tagged TdaD^{Pi}. The major peak at an elution volume of about 14.5 mL indicates that TdaD^{Pi} forms a stable tetramer (monomer: ~18 kDa) in solution.

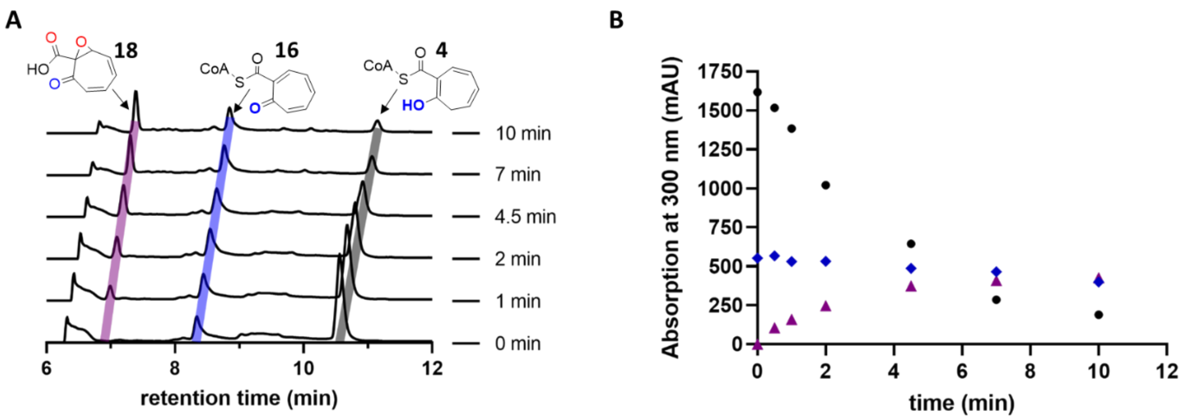

Figure S13: TdaE^{Bp}-catalyzed turnover of compound 4 **into compound** 18**. A,** HPLC-chromatograms of samples withdrawn from enzyme assays with TdaE^{Bp} after 0, 1, 2, 4.5, 7, and 10 min. Peaks corresponding to the substrate **4**, the reaction intermediate **16** and the final product **18** are indicated by *black*, *blue* and *purple* lines, respectively. **B**, Time-dependent changes in the peak areas corresponding to substrate **4** (*black dots*), the reaction intermediate **16** (*blue diamonds*) and the final product **18** (*purple triangles*).

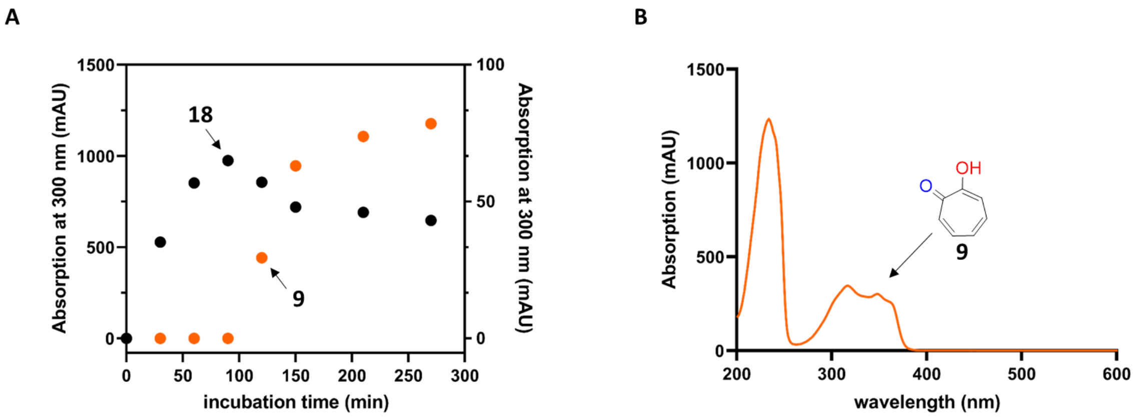

Figure S14: Time course of spontaneous conversion of compound 18 **into compound** 9 **in the enzyme reaction. A,** Assays containing **18** in 50 mM Tris-HCl were incubated at 30 °C and 900 rpm. Samples taken at different time points (0, 30, 60, 90, 120, 150, 210 and 270 min) were quenched with EtOAc + 1% FA and the organic phase was analyzed by RP-HPLC. The *black* dots present the time course for the degradation of compound **18** (left Y axis), and the *orange* dots indicate the time course for compound **9** (right Y axis) formation. **B,** Characteristic UV-visible absorption spectrum of compound **9** with a maximum absorption at around 234 nm, 317 nm and 348 nm.

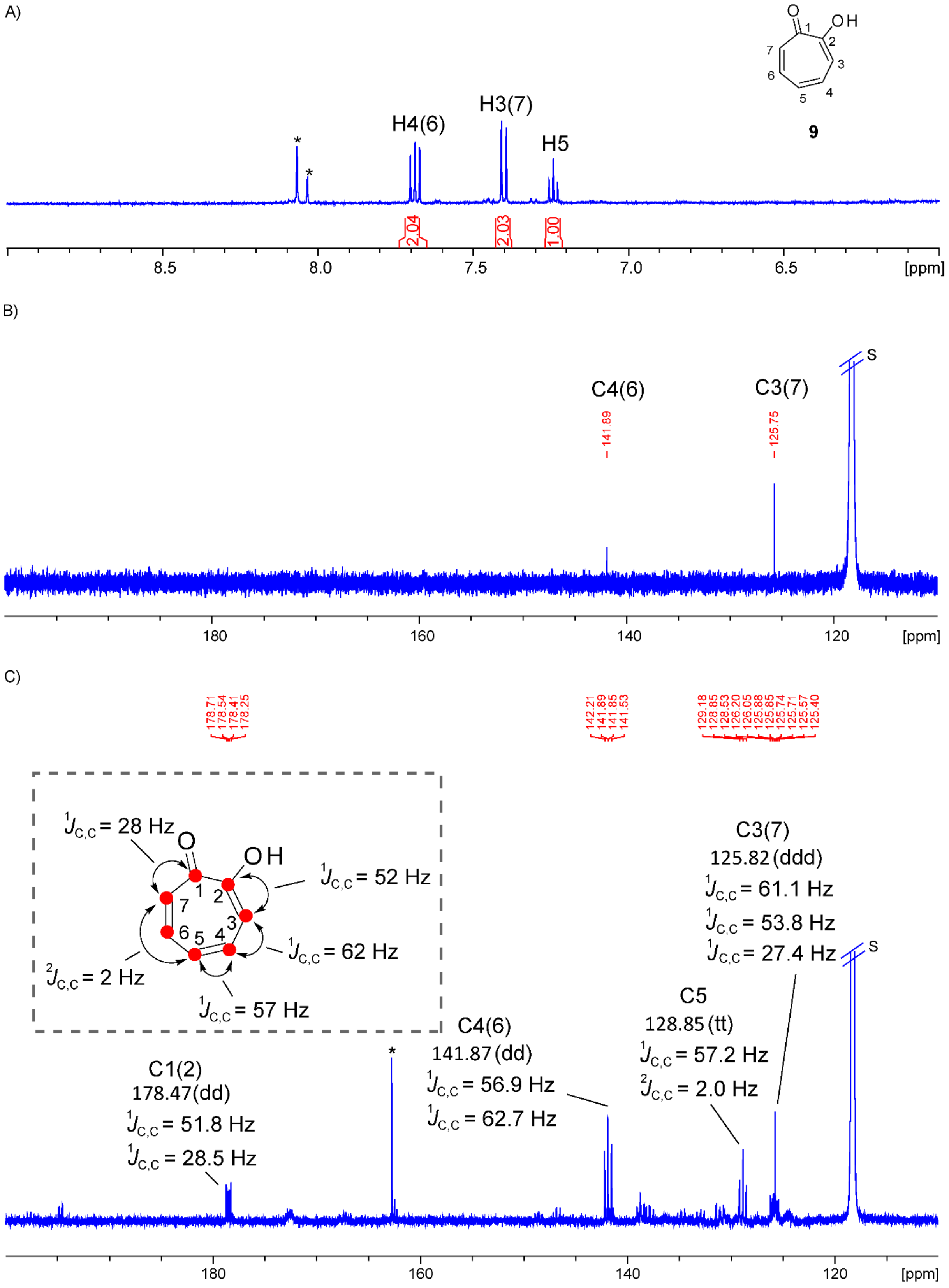

Figure S15: A, ^{1}H-NMR spectrum (700 MHz, CD_3CN) and **B**, ^{13}C-NMR spectrum (176 MHz, CD_3CN) of the enzymatically prepared unlabeled **9**. Because of the low concentration of the sample, the signals for C1(2) and C5 were missing in the ^{13}C-NMR spectrum. **C**, ^{13}C-NMR spectrum and the carbon coupling constants of ($^{13}C_7$)-**9** obtained enzymatically from ($^{13}C_8$)-**2**. Asterisks indicate the peaks of impurities obtained from the enzymatic reaction.

A)

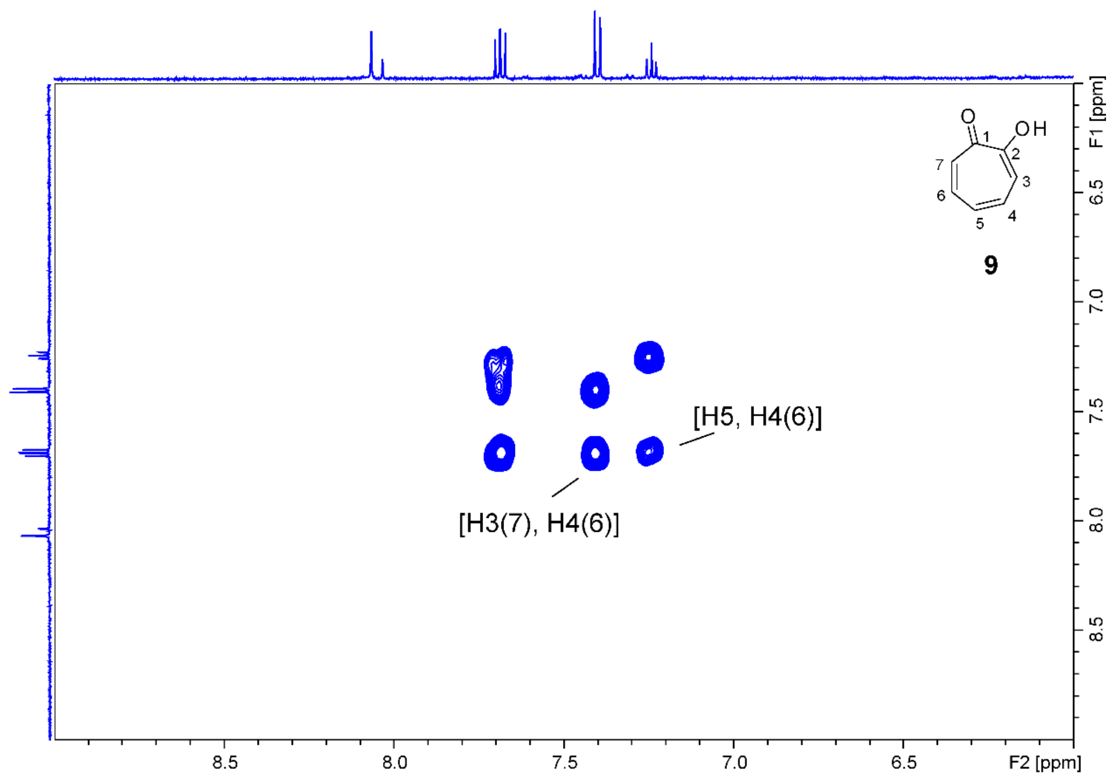

B)

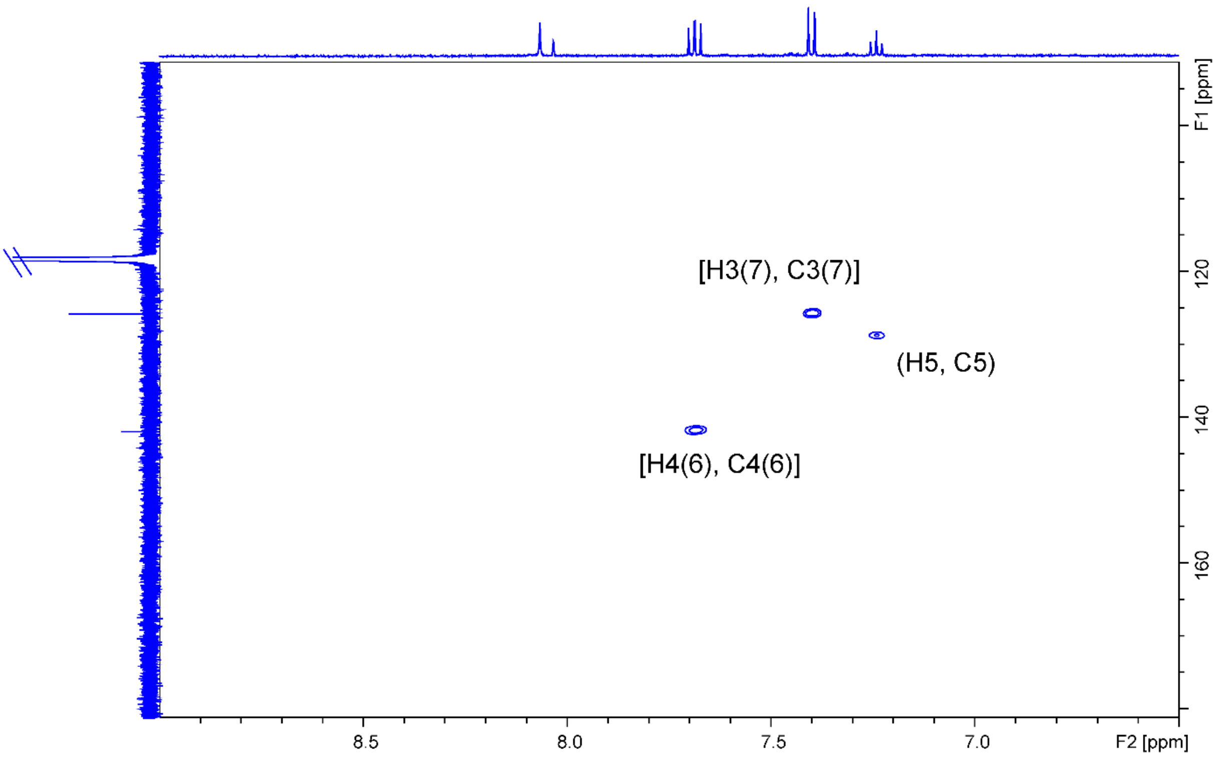

Figure S16: A, ^{1}H–^{1}H COSY spectrum and **B**, HSQC spectrum of unlabeled **9** from enzymatic reaction.

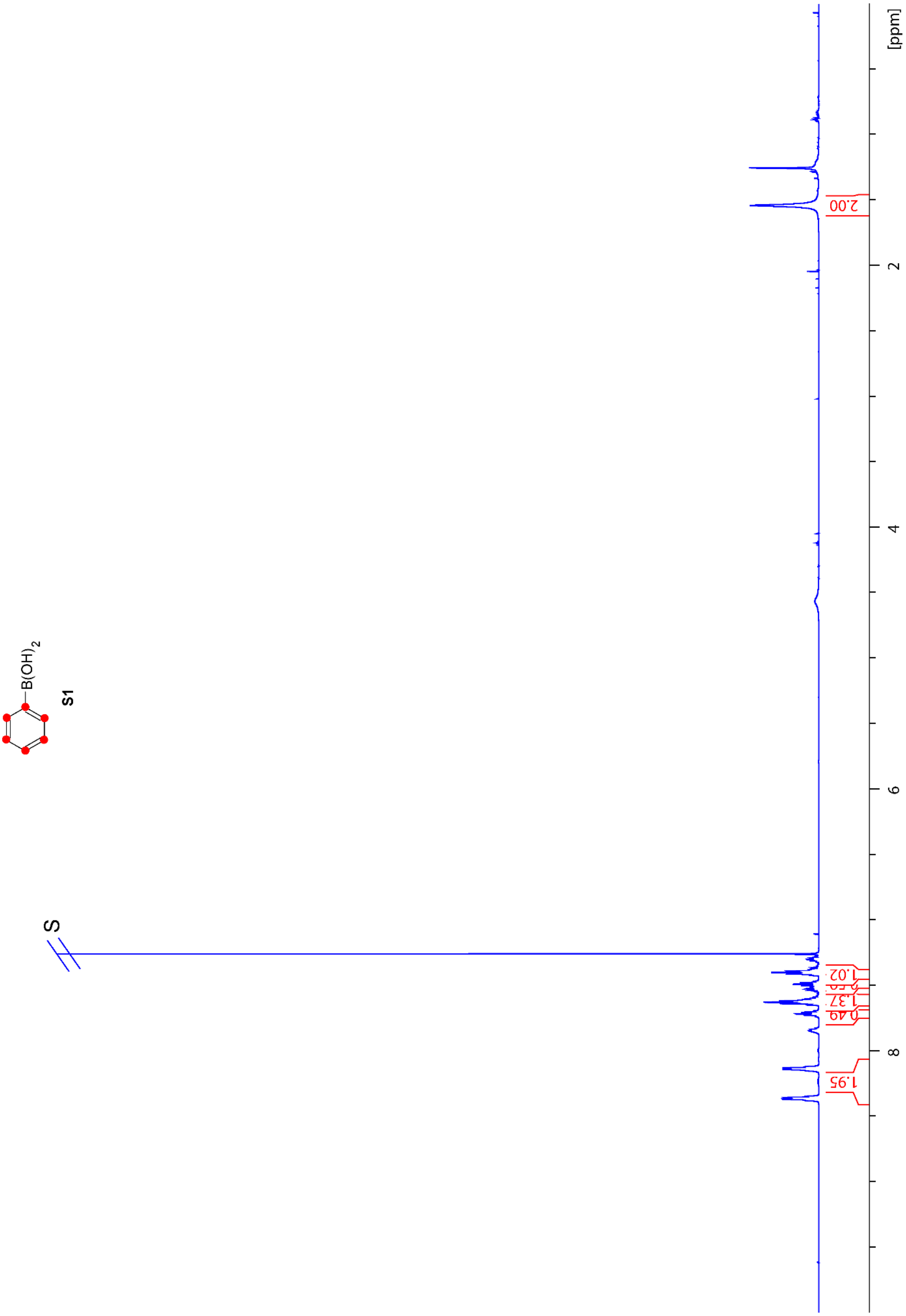

Figure S17: ^{1}H-NMR spectrum (700 MHz, CD_3Cl) of S1. S indicates solvent peak.

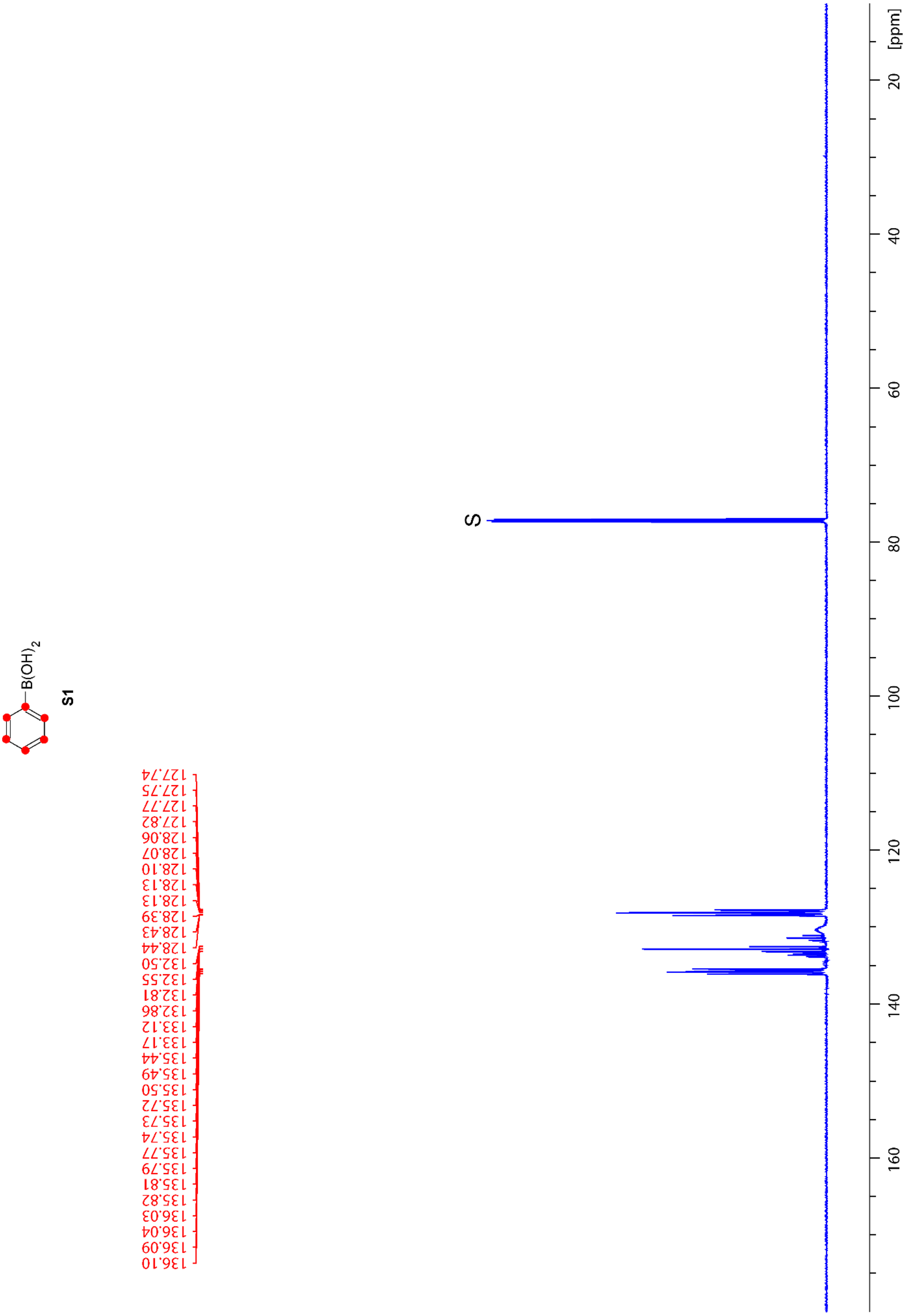

Figure S18: ^{13}C-NMR spectrum (176 MHz, CD_3Cl) of S1. S indicates solvent peak.

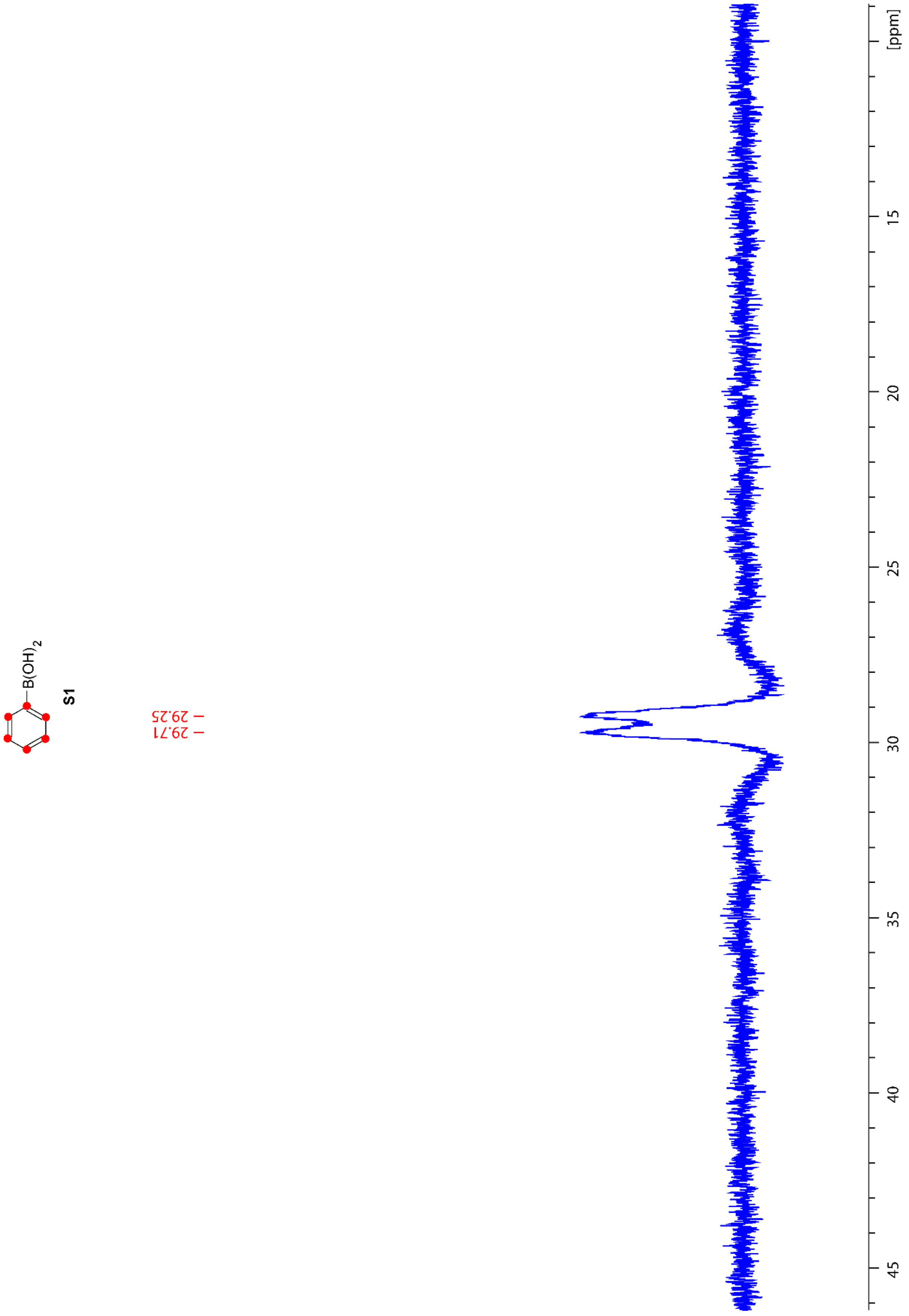

Figure S19: ^{11}B-NMR spectrum (160 MHz, CD_3Cl) of S1.

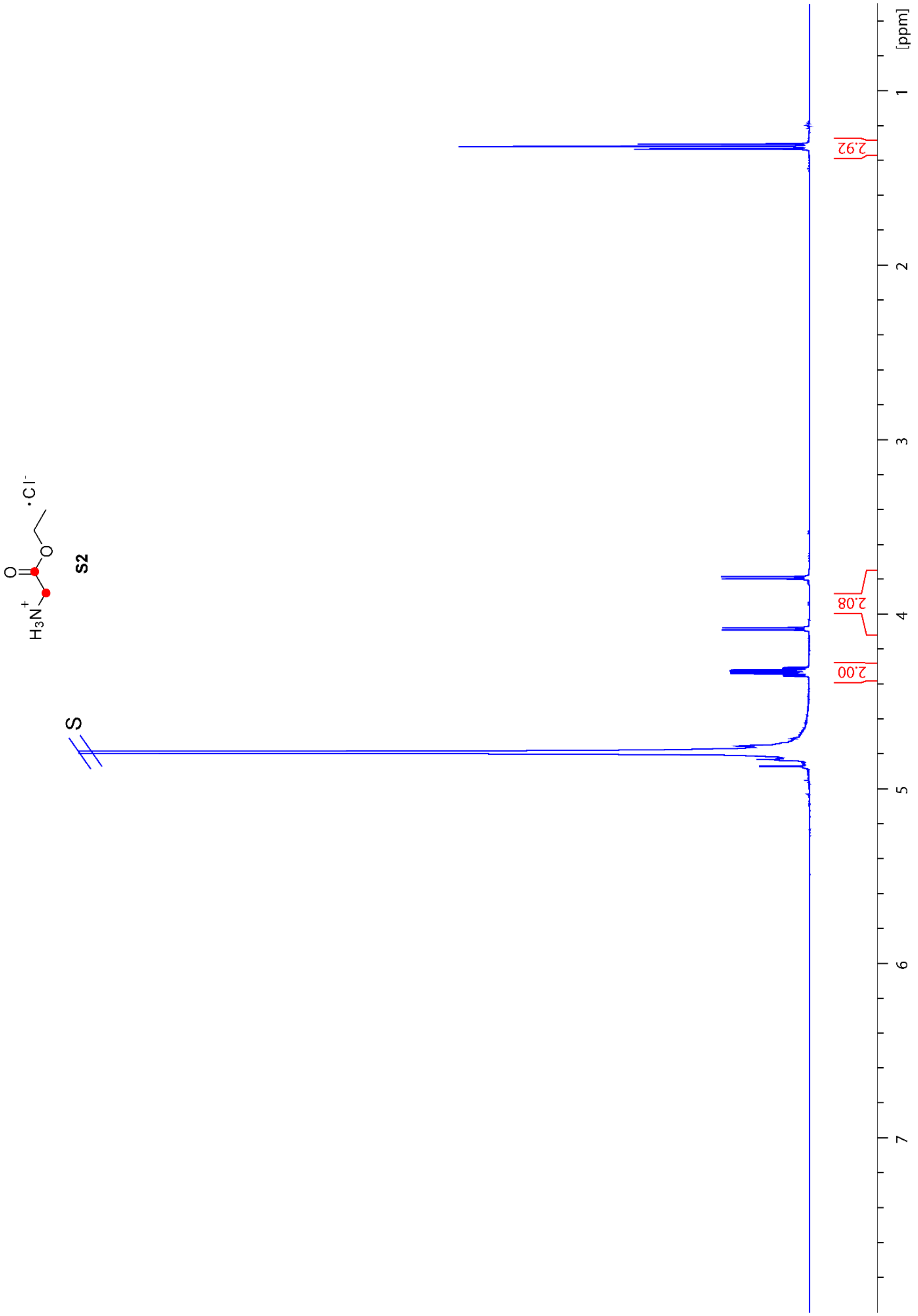

Figure S20: ^{1}H-NMR spectrum (500 MHz, D_2O) of S2. S indicates solvent peak.

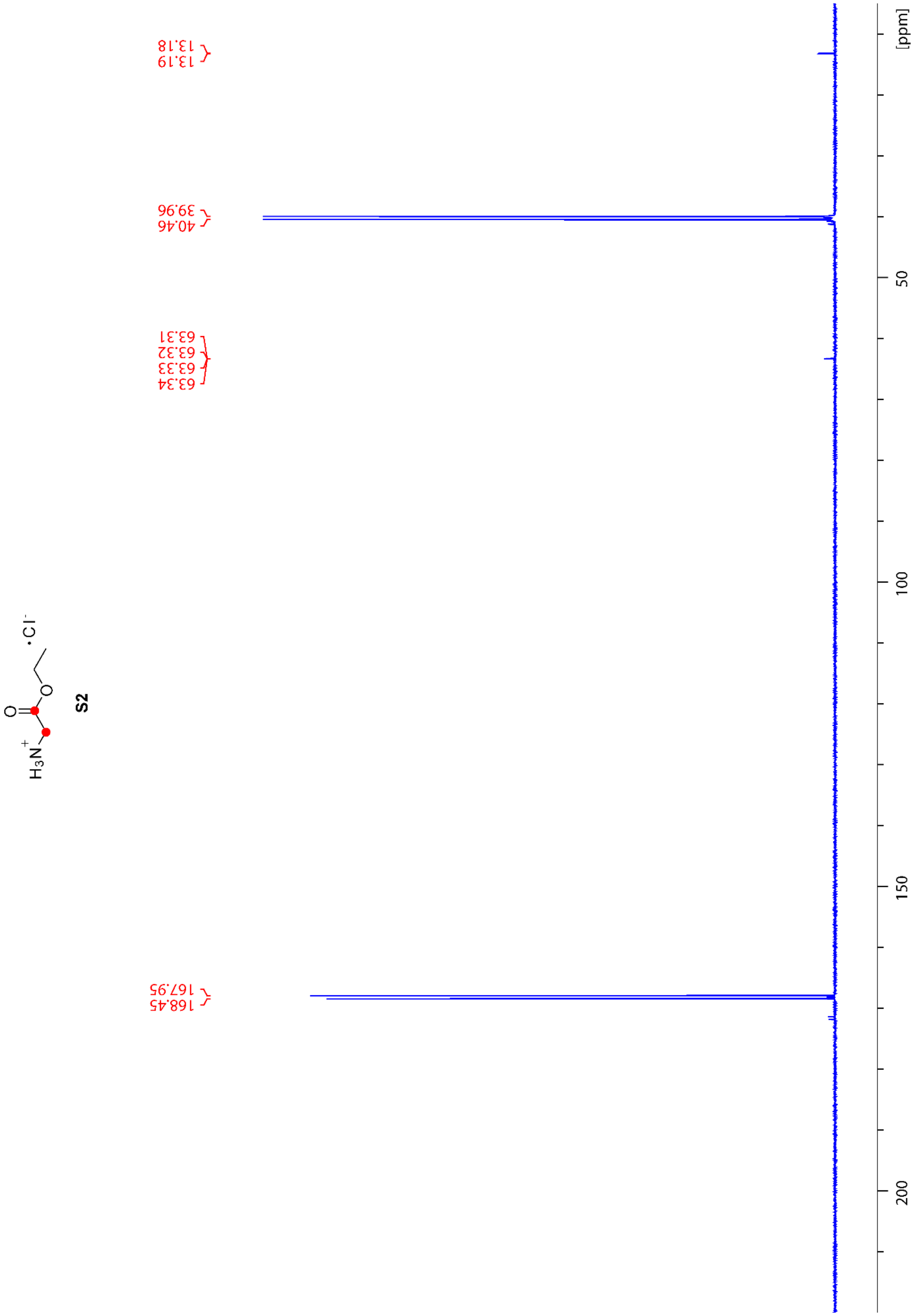

Figure S21: ^{13}C-NMR spectrum (126 MHz, D_2O) of S2.

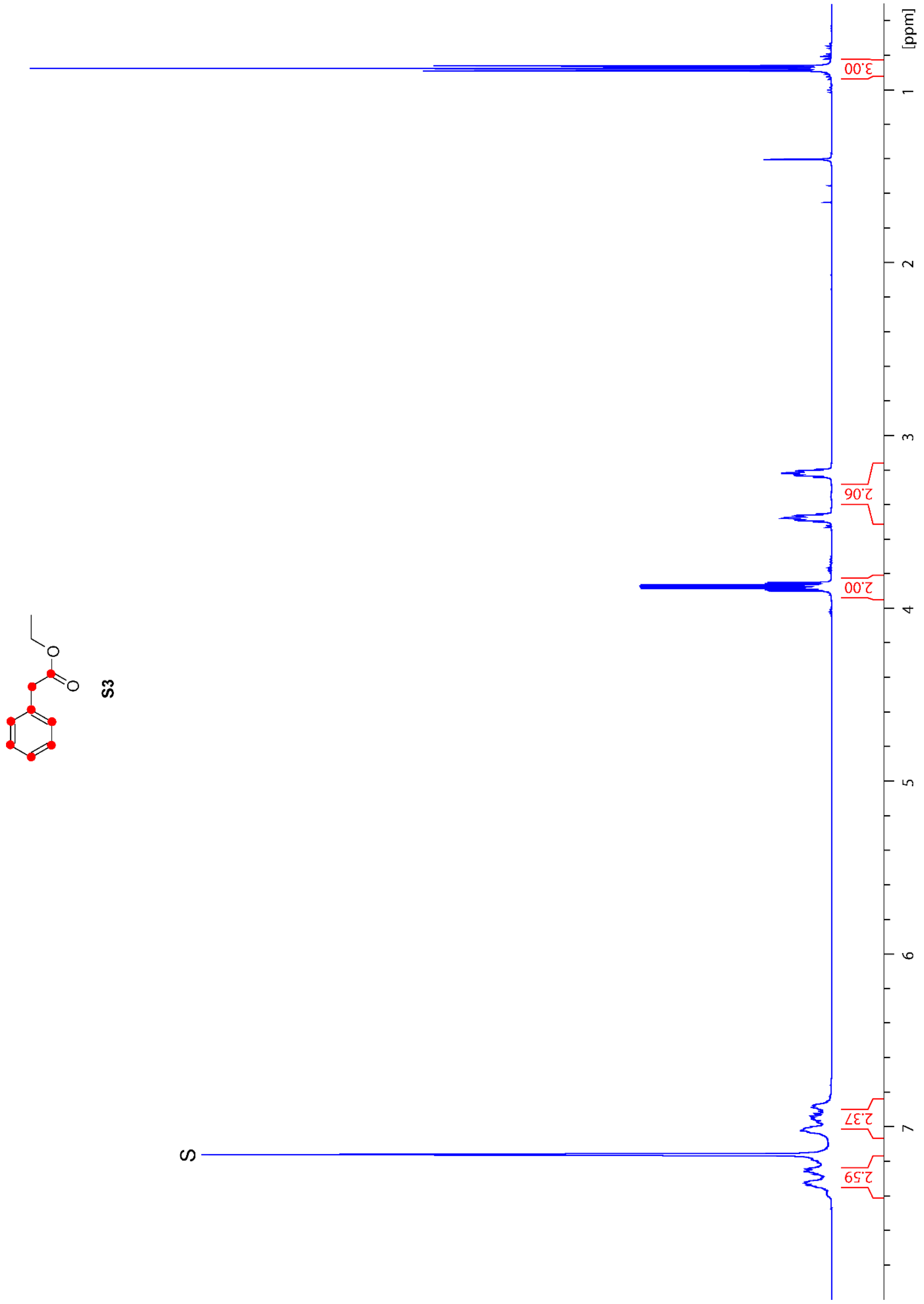

Figure S22: ^{1}H-NMR spectrum (500 MHz, C_6D_6) of S3. S indicates solvent peak.

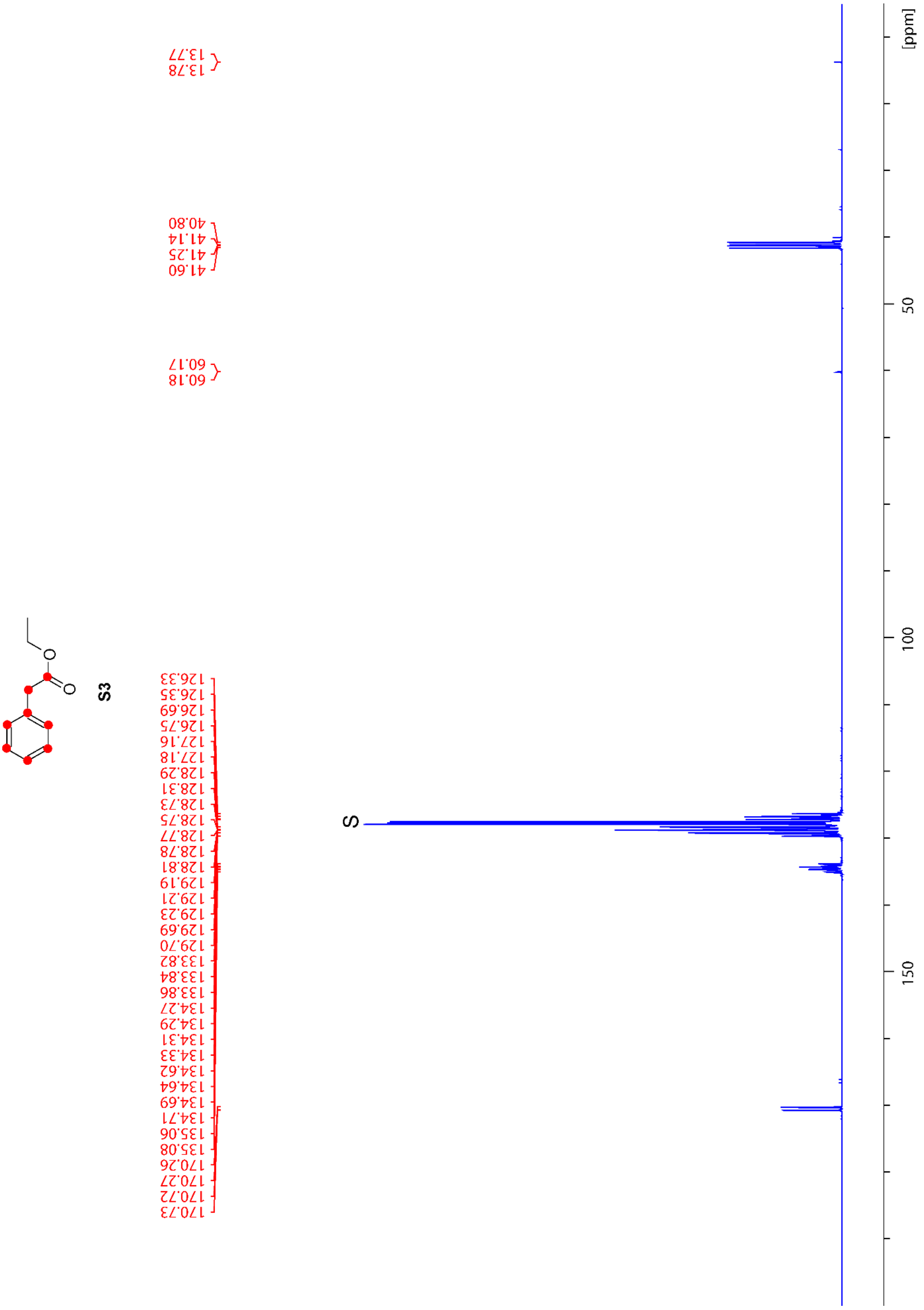

Figure S23: ^{13}C-NMR spectrum (126 MHz, C_6D_6) of S3. S indicates solvent peak.

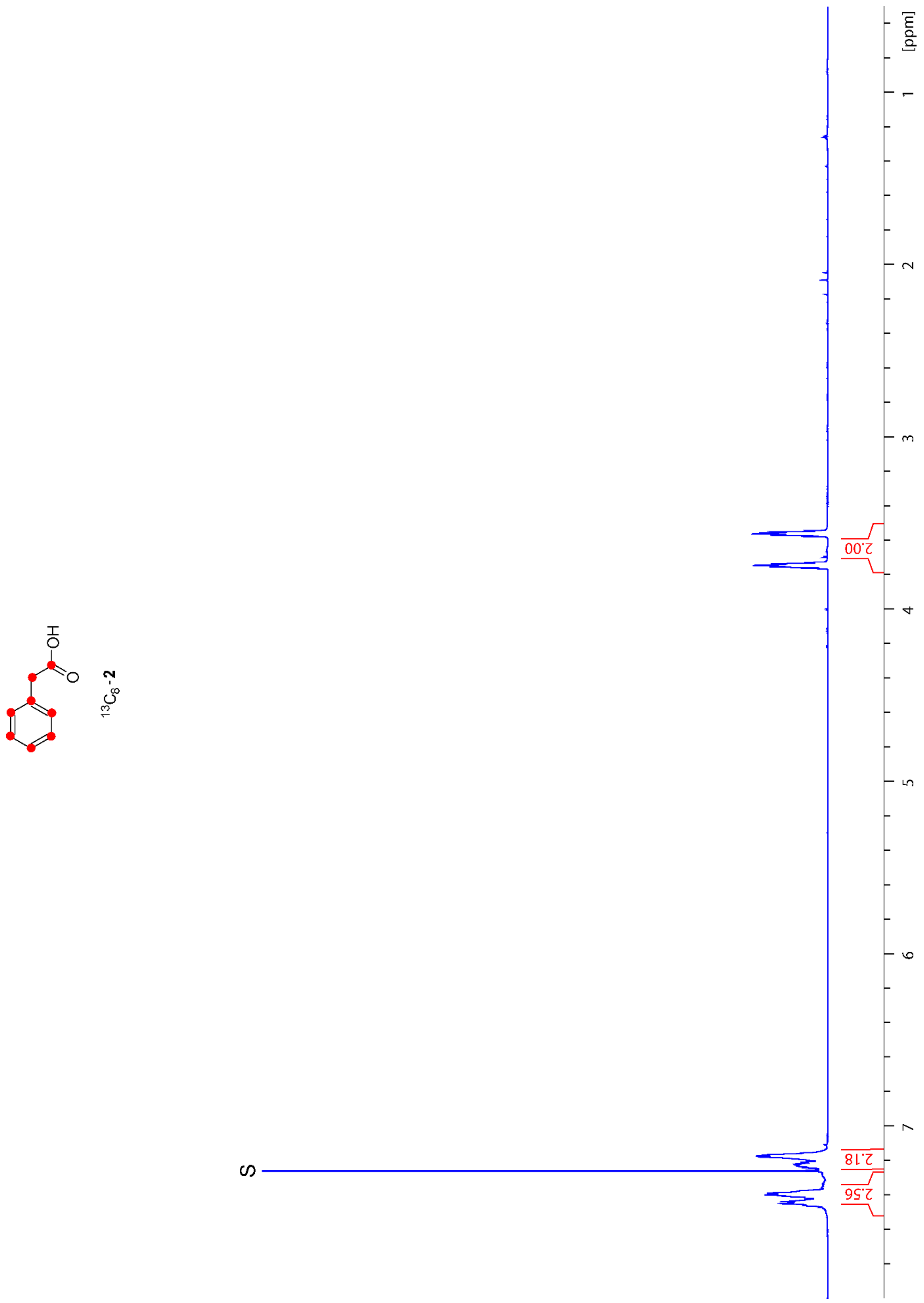

Figure S24: ^{1}H-NMR spectrum (700 MHz, CD_3Cl) of ($^{13}C_8$)-2. S indicates solvent peak.

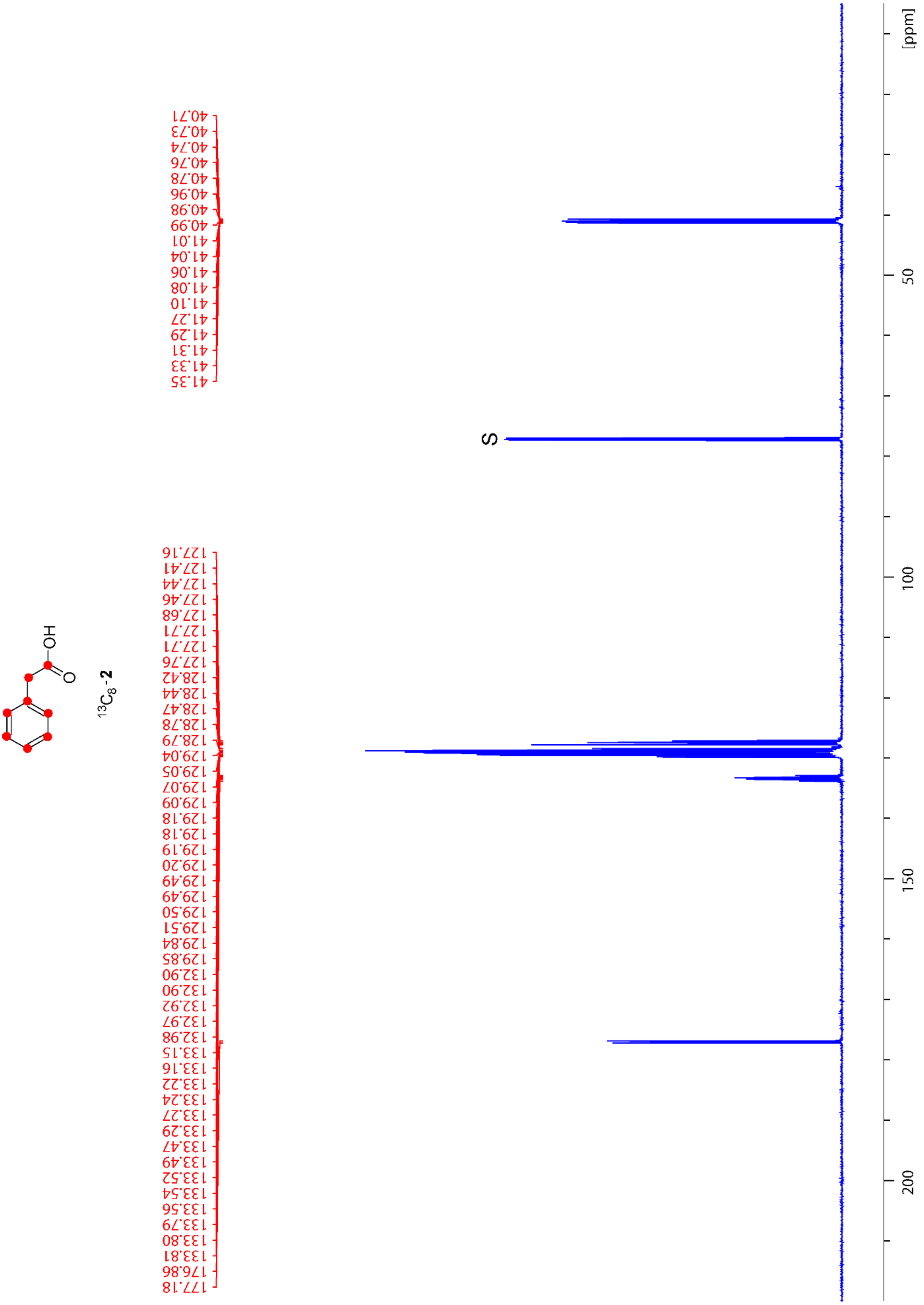

Figure S25: ^{13}C-NMR spectrum (176 MHz, CD_3Cl) of ($^{13}C_8$)-2. S indicates solvent peak.

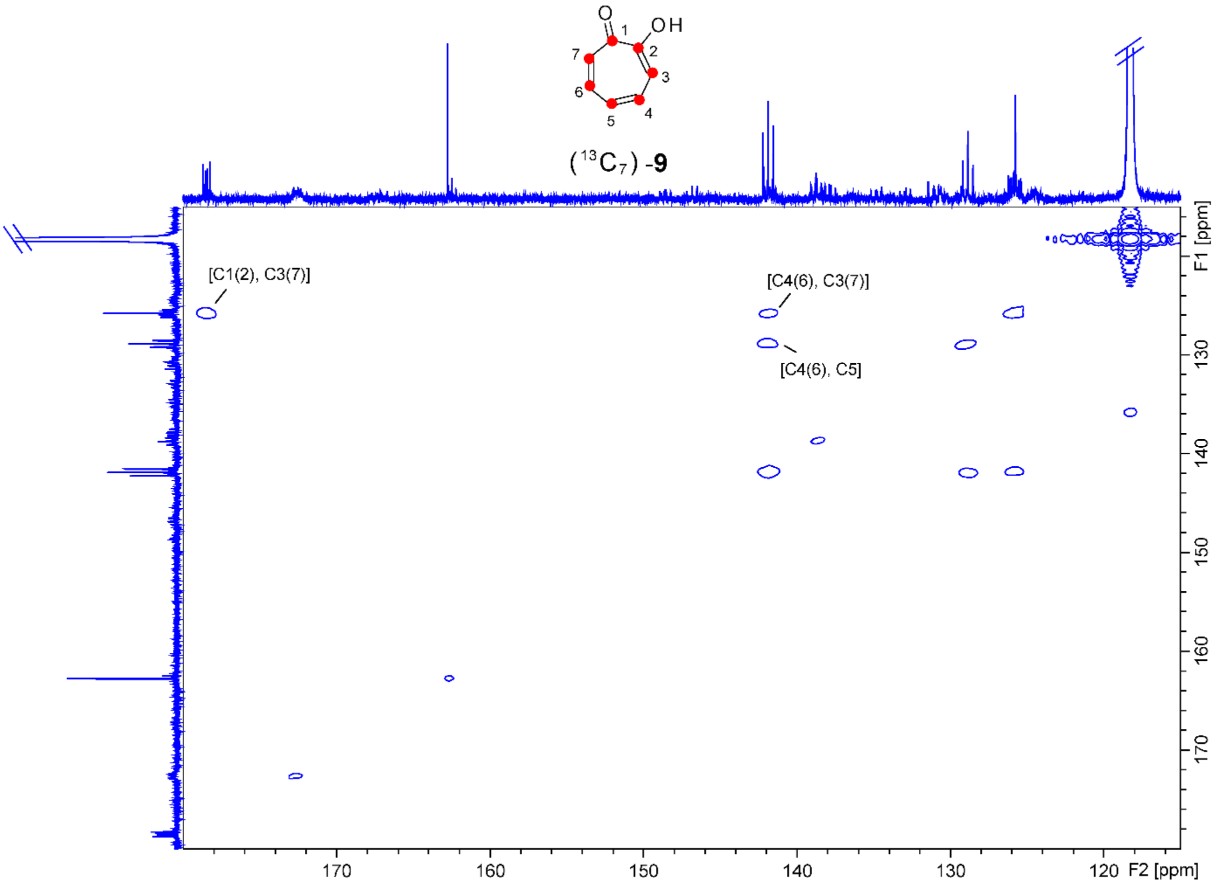

Figure S26: ^{13}C–^{13}C COSY spectrum of ($^{13}C_7$)-9 obtained enzymatically from ($^{13}C_8$)-2.

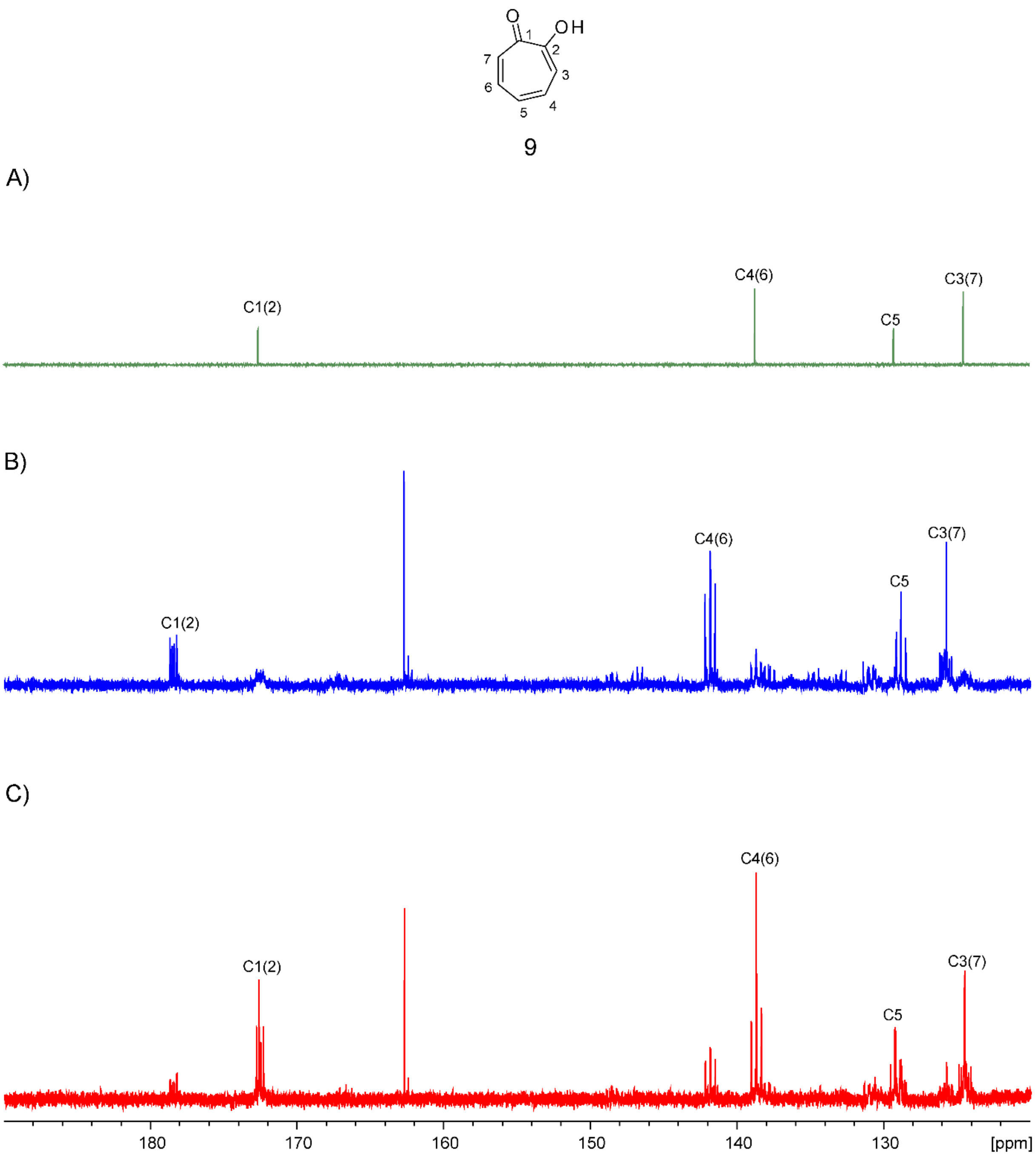

Figure S27: Comparison of ($^{13}C_7$)-9 to a commercial tropolone standard. A, ^{13}C-NMR spectrum of commercial tropolone (**9**); **B**, ^{13}C-NMR spectrum of enzymatically obtained ($^{13}C_7$)-**9**; **C**, ^{13}C-NMR spectrum of tropolone (**9**) mixed with ($^{13}C_7$)-**9**. The chemical shifts in the ^{13}C-NMR spectrum of **9** are known to depend on the presence of different metal cations and are also strongly dependent on pH[15,16], so the difference between the signals of **9** in **A** and ($^{13}C_7$)-**9** in **B** are likely influenced by the presence of different metal cations in different concentrations and/or by a different pH. Spiking of the sample from **B** with commercial tropolone confirms the identity of the enzymatically obtained ($^{13}C_7$)-**9** as shown in **C**.

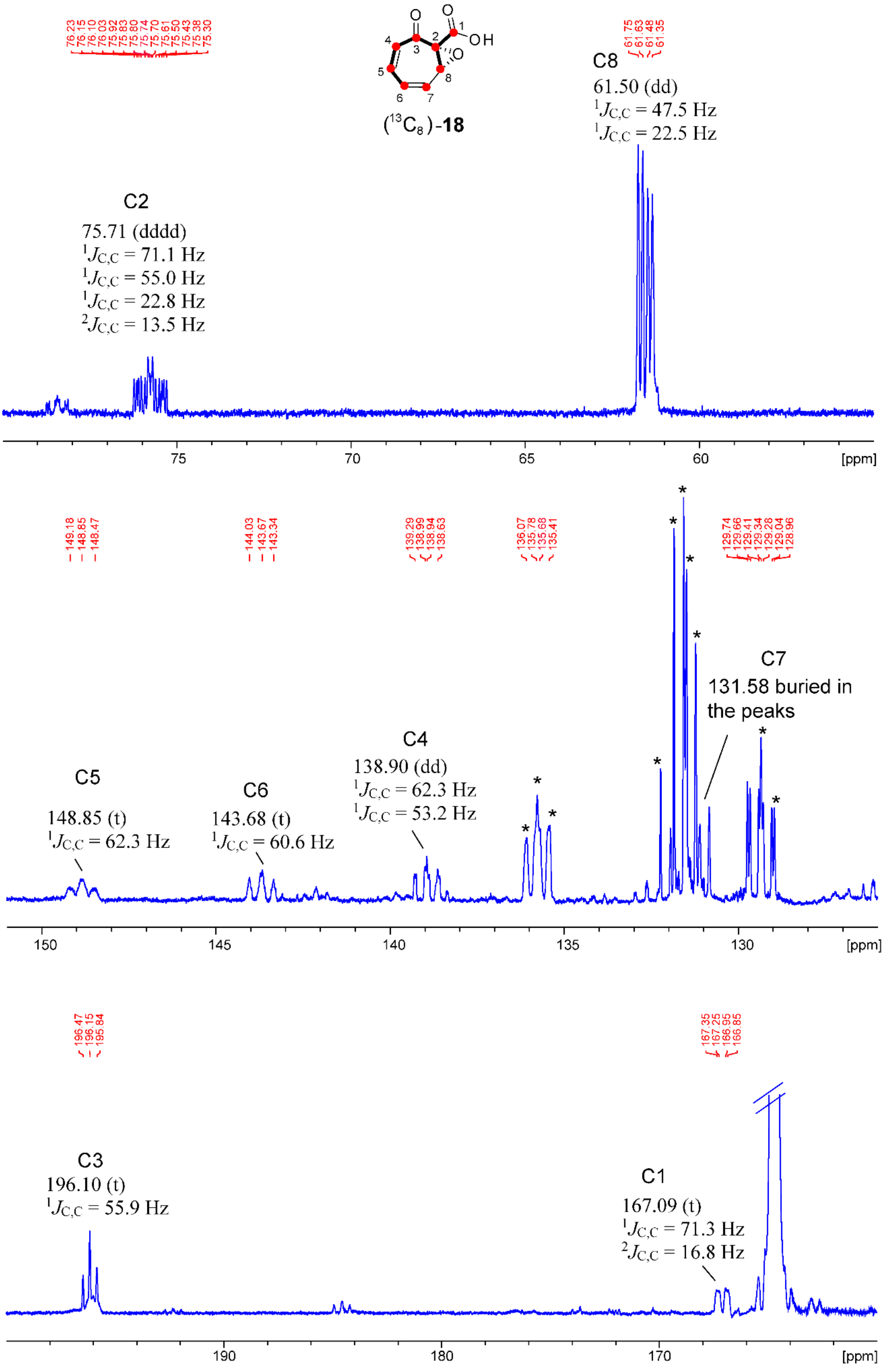

Figure S28: ^{13}C-NMR spectrum of ($^{13}C_8$)-18 (700 MHz, CD_3CN). Signals for eight carbons are observed, their connection can be concluded from the ^{13}C,^{13}C-coupling patterns as indicated by bold bonds in the structure. Asterisks indicate impurities arising from side reactions in the enzymatic reaction.

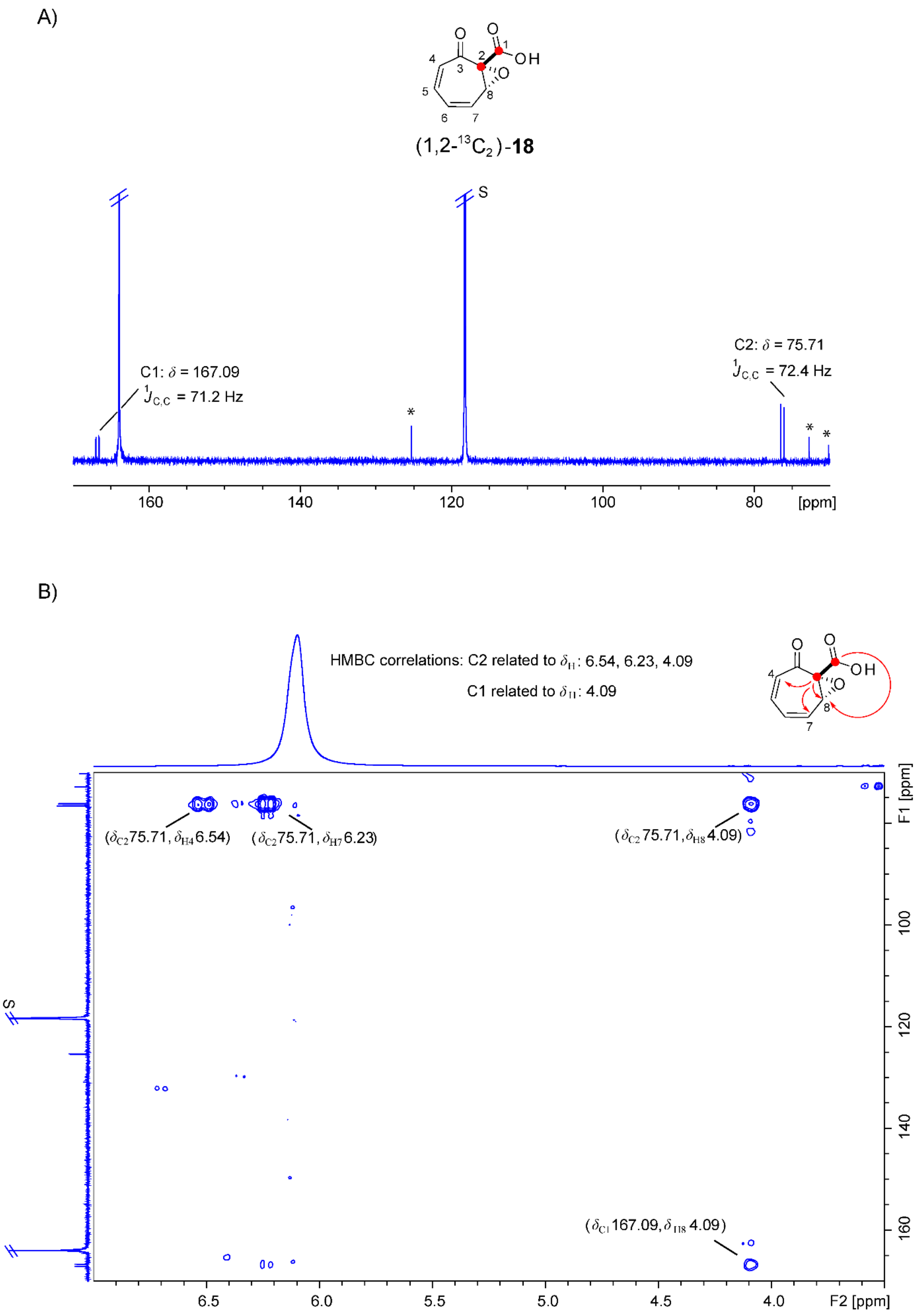

Figure S29: ^{13}C-NMR spectrum and HMBC spectrum of (1,2-^{13}C$_2$)-18. **A**, Signals for C1 and C2 are observed and the coupling constant $^1J_{C,C}$ indicates their direct connection (*bold* bond in the structure). **B**, HMBC correlations indicate the connection of the C1/C2 portion to C4, C7 and C8.

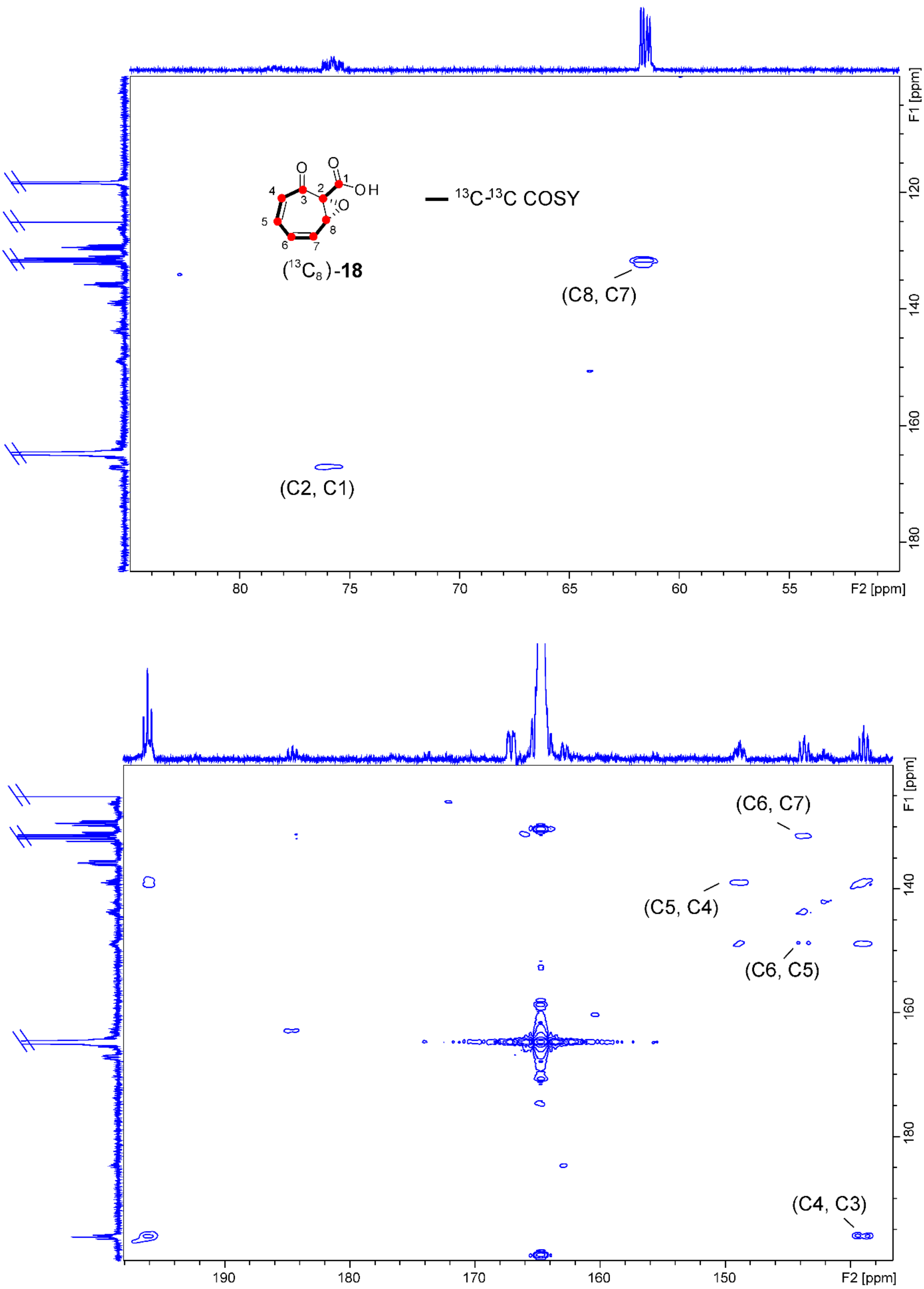

Figure S30: ^{13}C–^{13}C COSY spectrum of ($^{13}C_8$)-18. The observed crosspeaks show direct connections between the carbons as indicated by the bold lines in the structure of ($^{13}C_8$)-**18**.

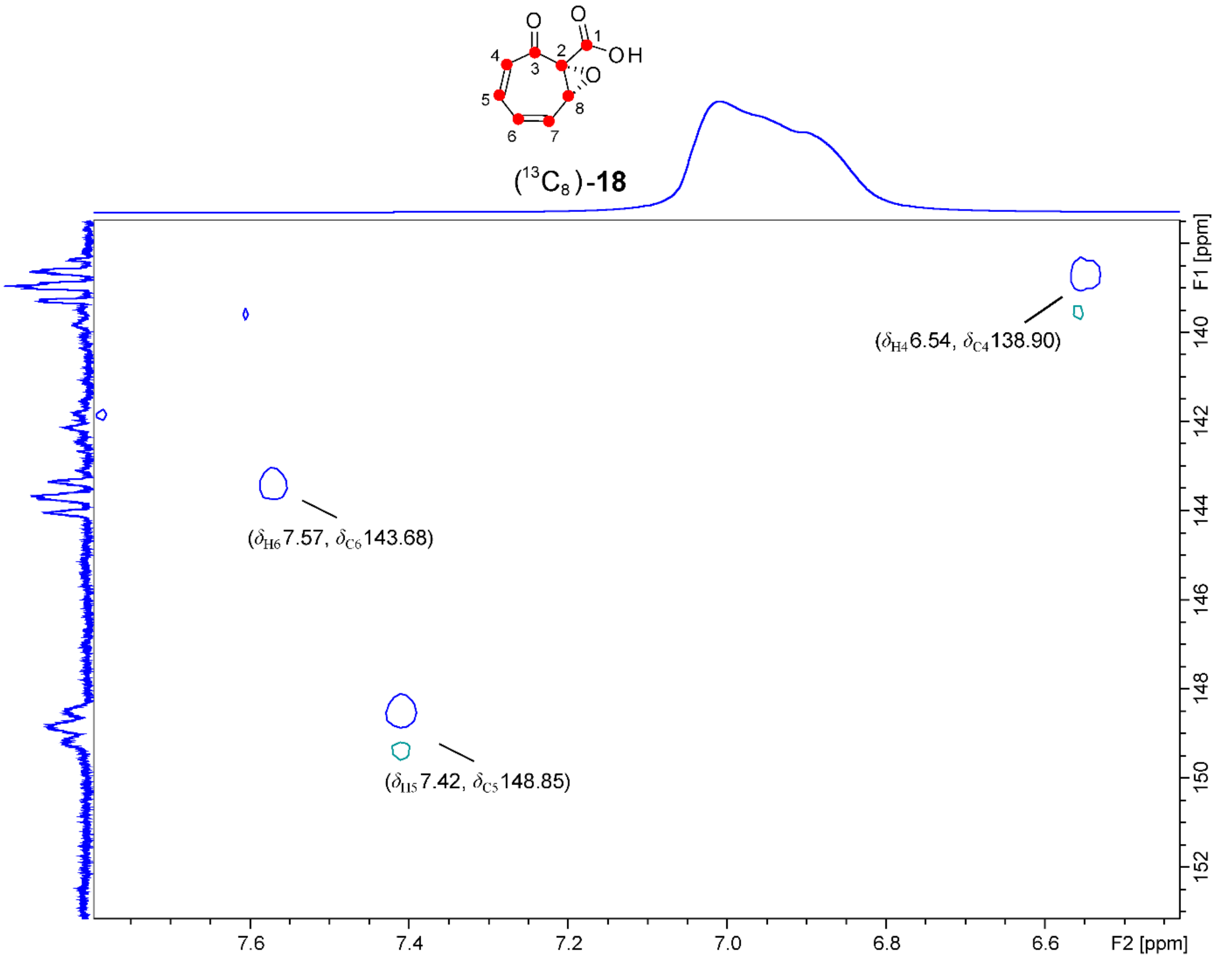

Figure S31: HSQC spectrum of ($^{13}C_8$)-18. The low concentration of the sample does not allow for the observation of ^1H signals in the ^1H-NMR spectrum (top 1D-spectrum), but the ^{13}C-labeling strongly enhances crosspeaks in the HSQC analysis, allowing for the detection of hydrogens through 2D-NMR.

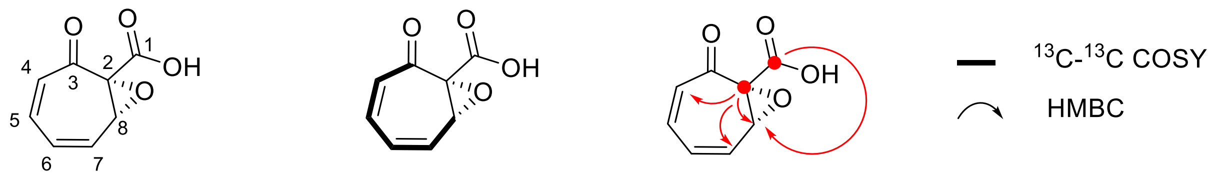

Table S4. Summary NMR data of 18 **in CD_3CN.**

C	^1H	^{13}C
1	–	167.09
2	–	75.71
3	–	196.10
4	6.54, 1H[a,b]	138.90
5	7.42, 1H[a]	148.85
6	7.57, 1H[a]	143.68
7	6.23, 1H[b]	131.58
8	4.09, 1H[b]	61.50

[a] Assigned from HSQC of ($^{13}C_8$)-**18**. [b] Assigned from HMBC of (1,2-$^{13}C_2$)-**18**.

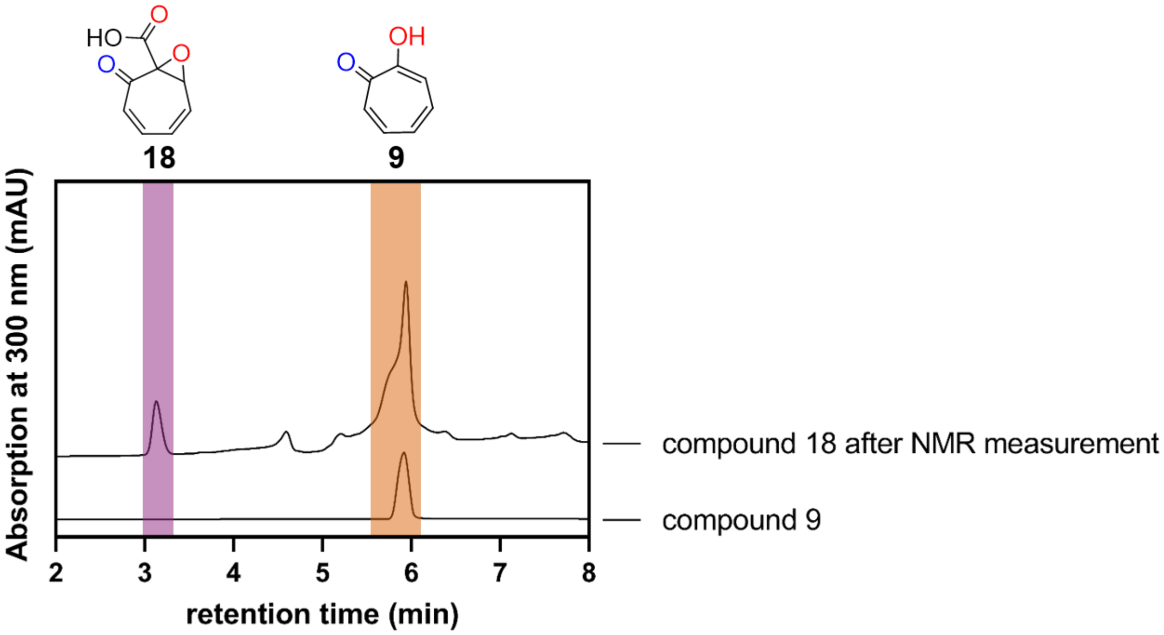

Figure S32: HPLC-DAD re-analysis of compound ($^{13}C_8$)-18**, which appeared to have degraded to ($^{13}C_7$)-**9 **during NMR measurement.** Compound **($^{13}C_8$)-18** was produced in large scale and purified by RP-HPLC for NMR. Since **9** was identified as the main metabolite in the NMR-sample, the latter was again analyzed by HPLC-DAD after NMR-measurement, revealing that more than 80% of compound **($^{13}C_8$)-18** was decomposed to **($^{13}C_7$)-9**.

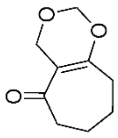

20

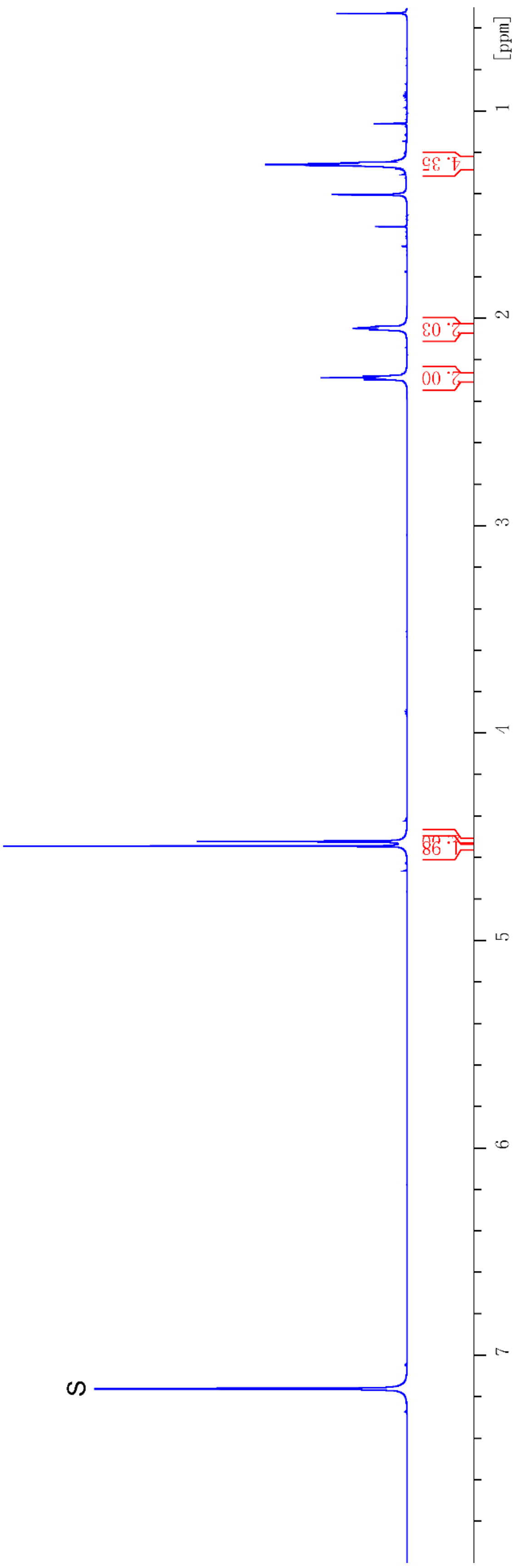

Figure S33: ^{1}H-NMR spectrum (700 MHz, C_6D_6) of 20. S indicates solvent peak.

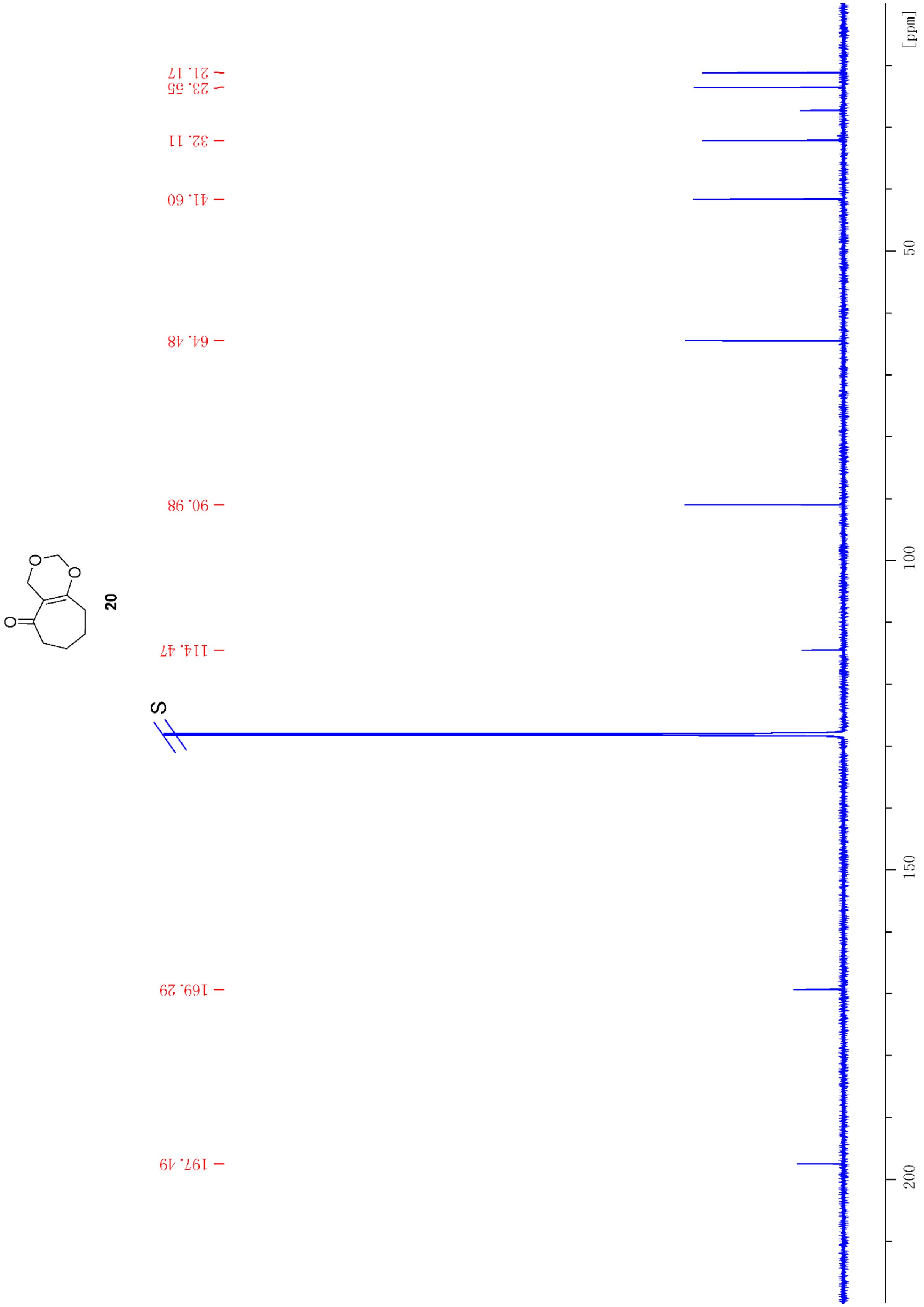

Figure S34: ^{13}C-NMR spectrum (176 MHz, C_6D_6) of 20. S indicates solvent peak.

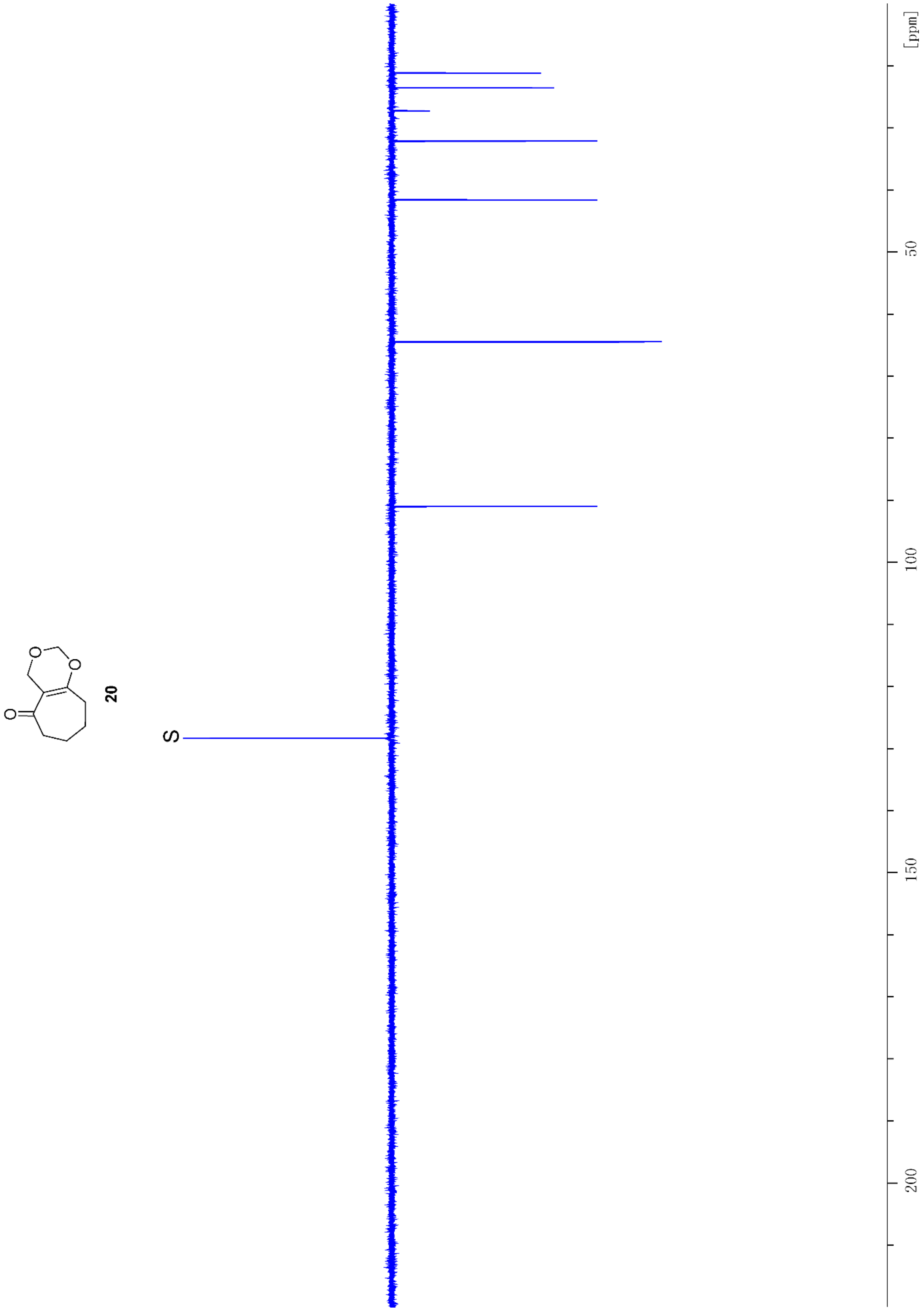

Figure S35: DEPT spectrum (176 MHz, C_6D_6) of 20. S indicates solvent peak.

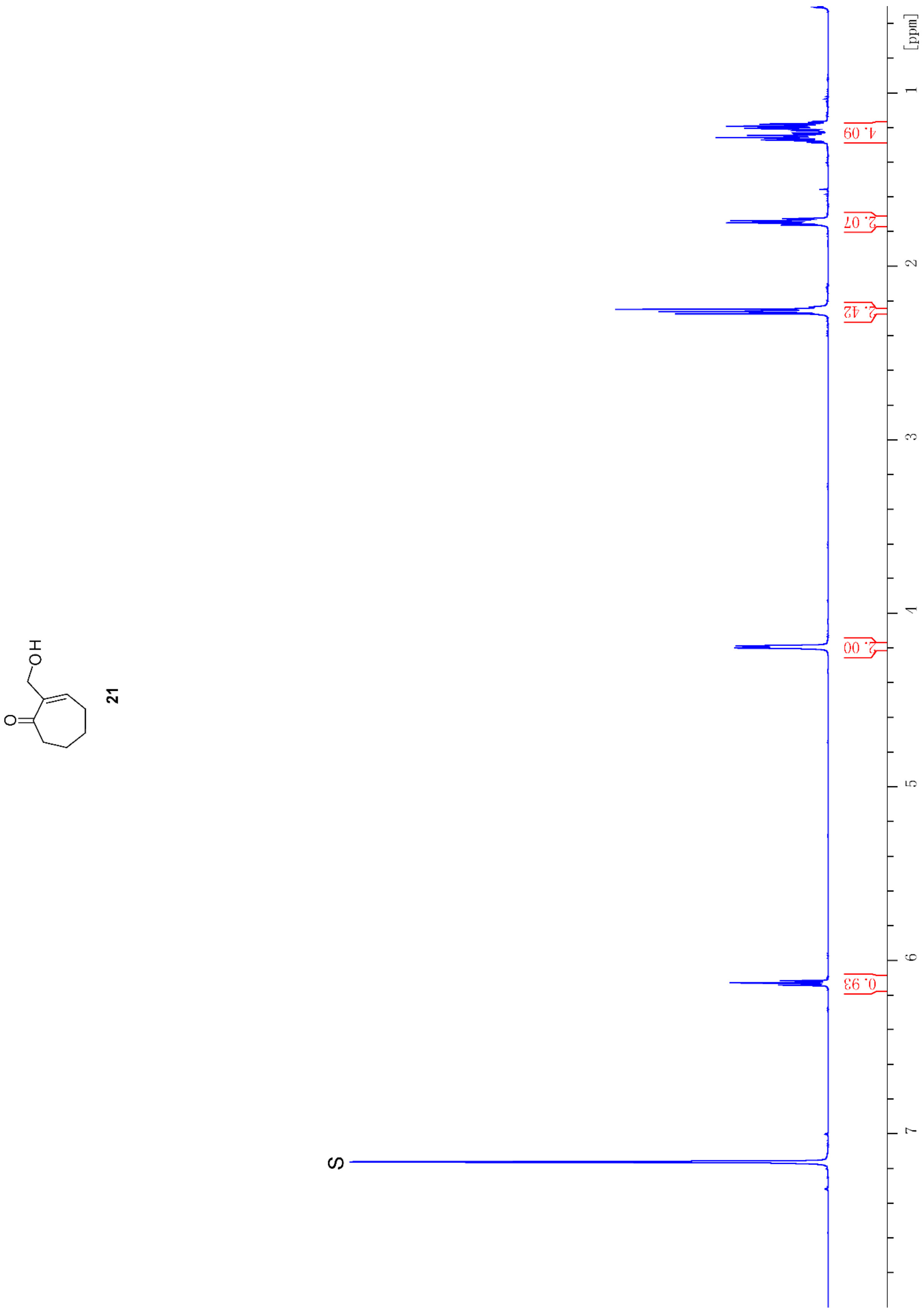

Figure S36: ^{1}H-NMR spectrum (400 MHz, C_6D_6) of 21. S indicates solvent peak.

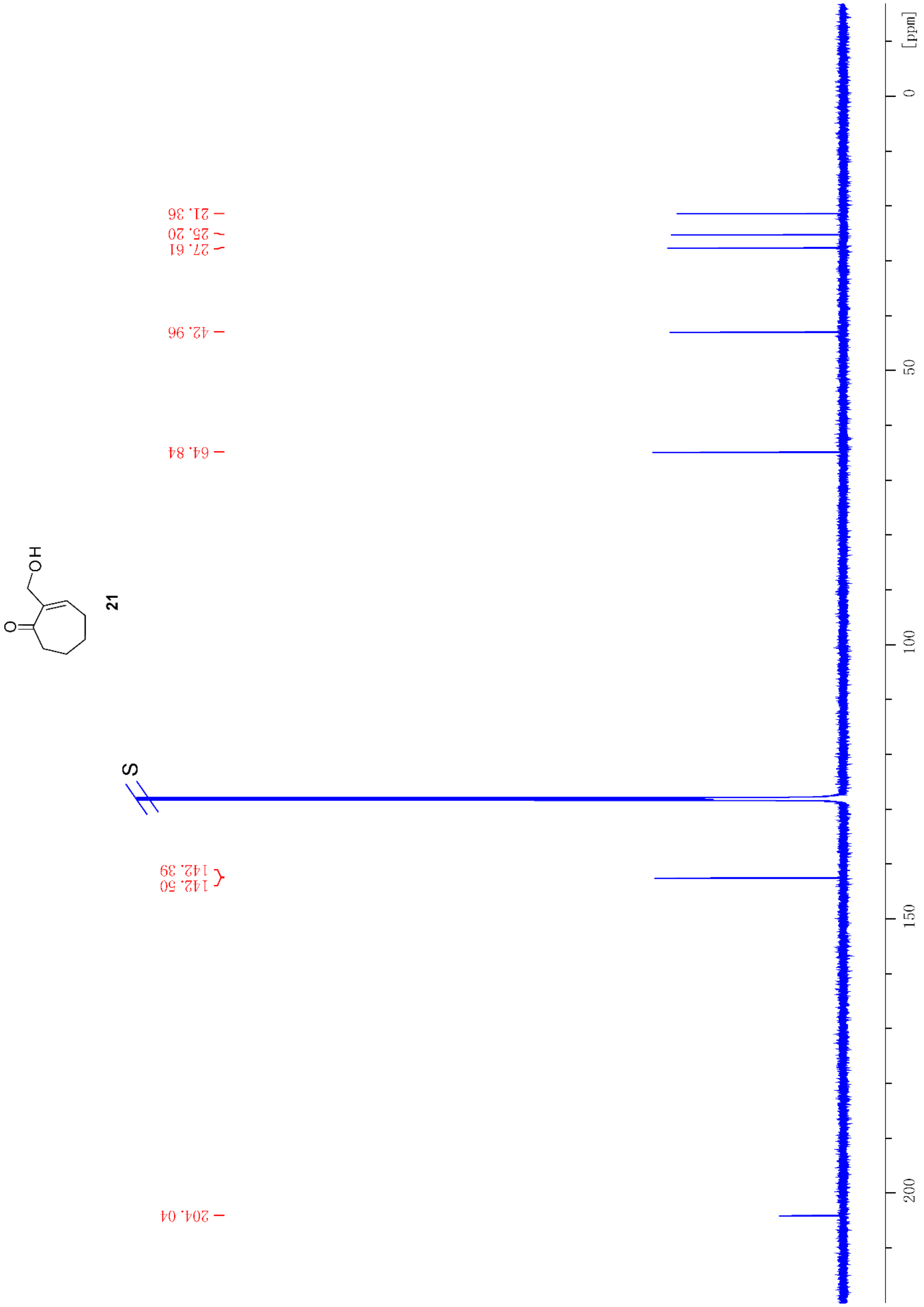

Figure S37: ^{13}C-NMR spectrum (101 MHz, C_6D_6) of 21. S indicates solvent peak.

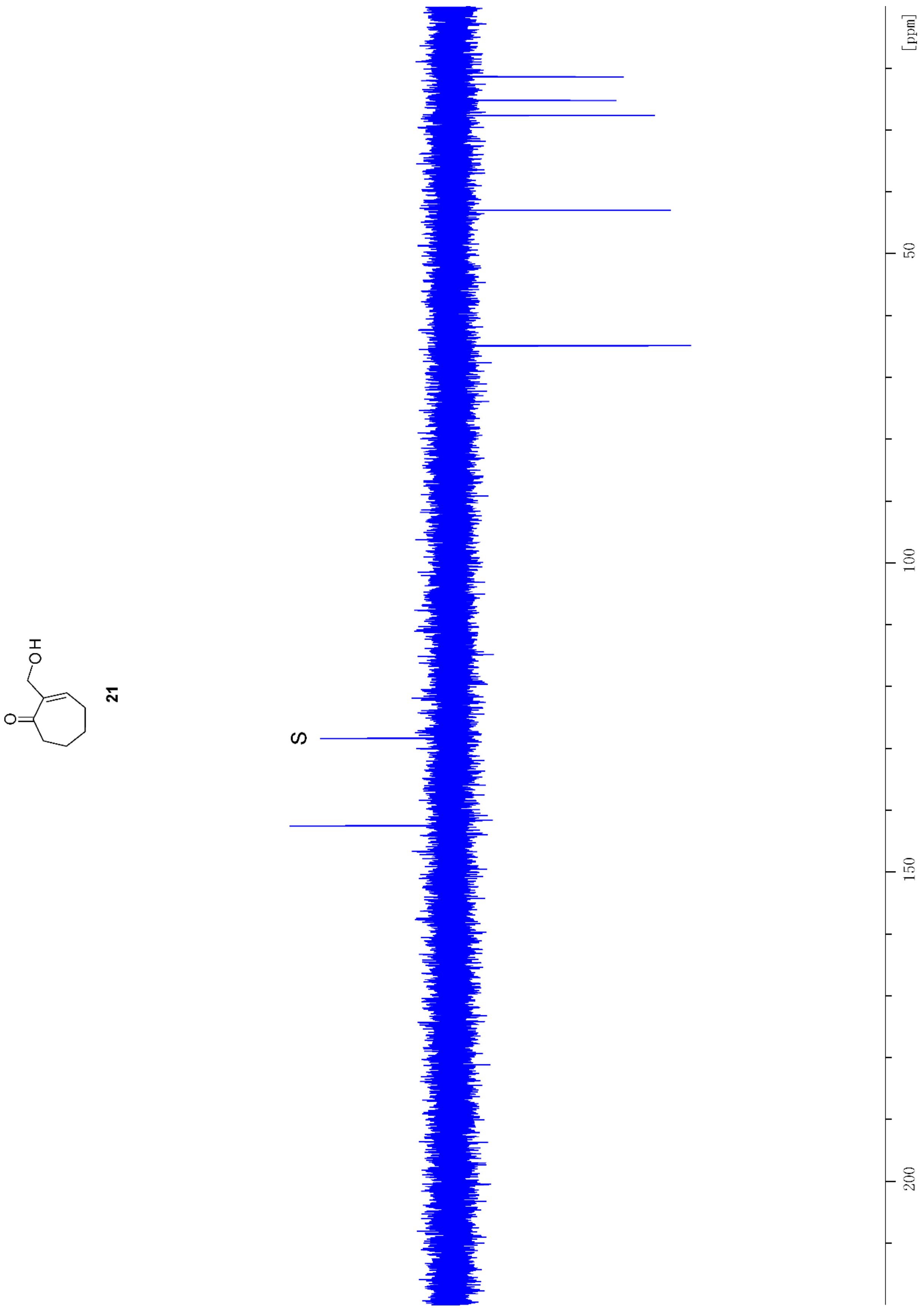

Figure S38: DEPT spectrum (101 MHz, C_6D_6) of 21. S indicates solvent peak.

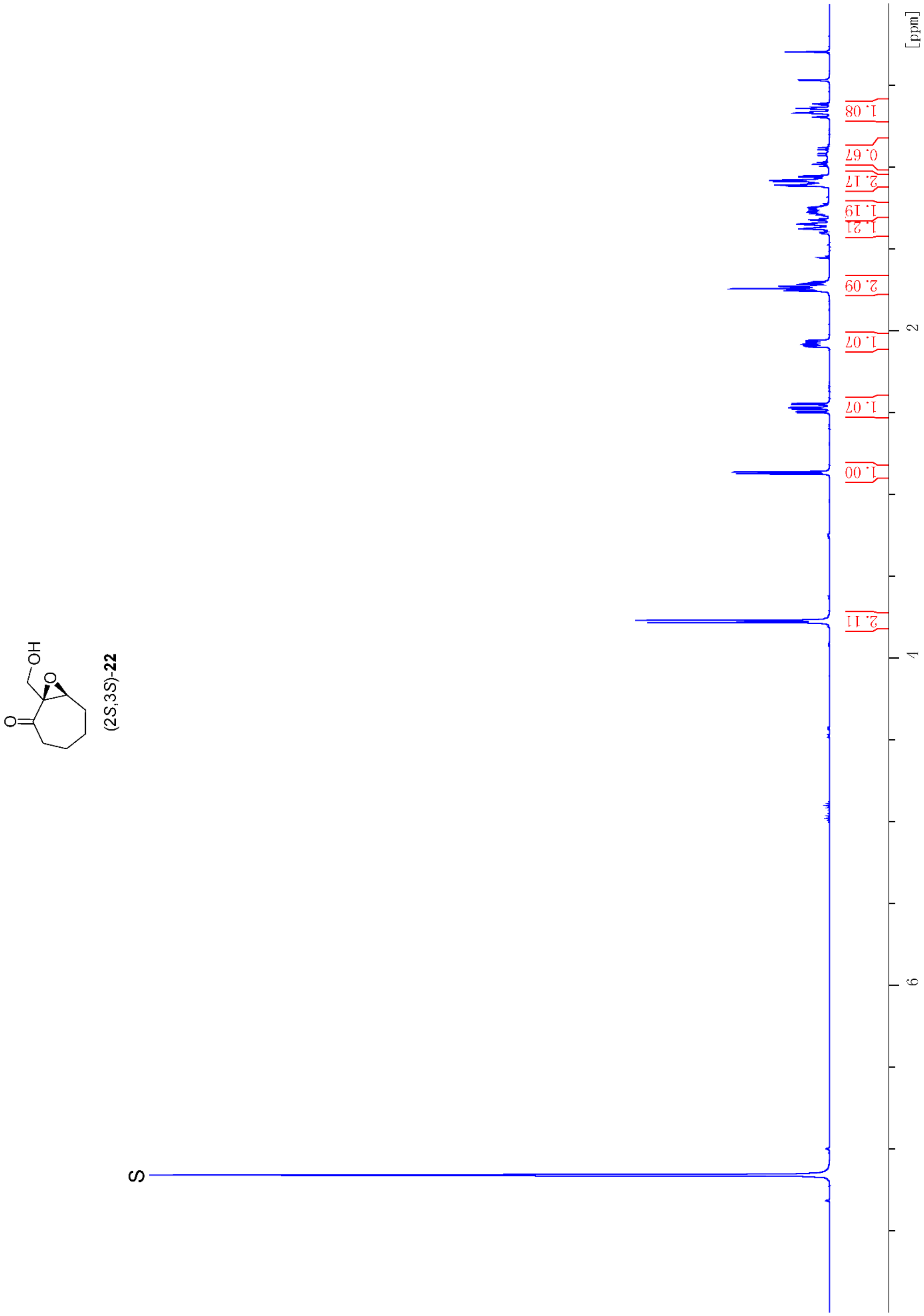

Figure S39: ^{1}H-NMR spectrum (500 MHz, C_6D_6) of (2*S*,3*S*)-22. S indicates solvent peak.

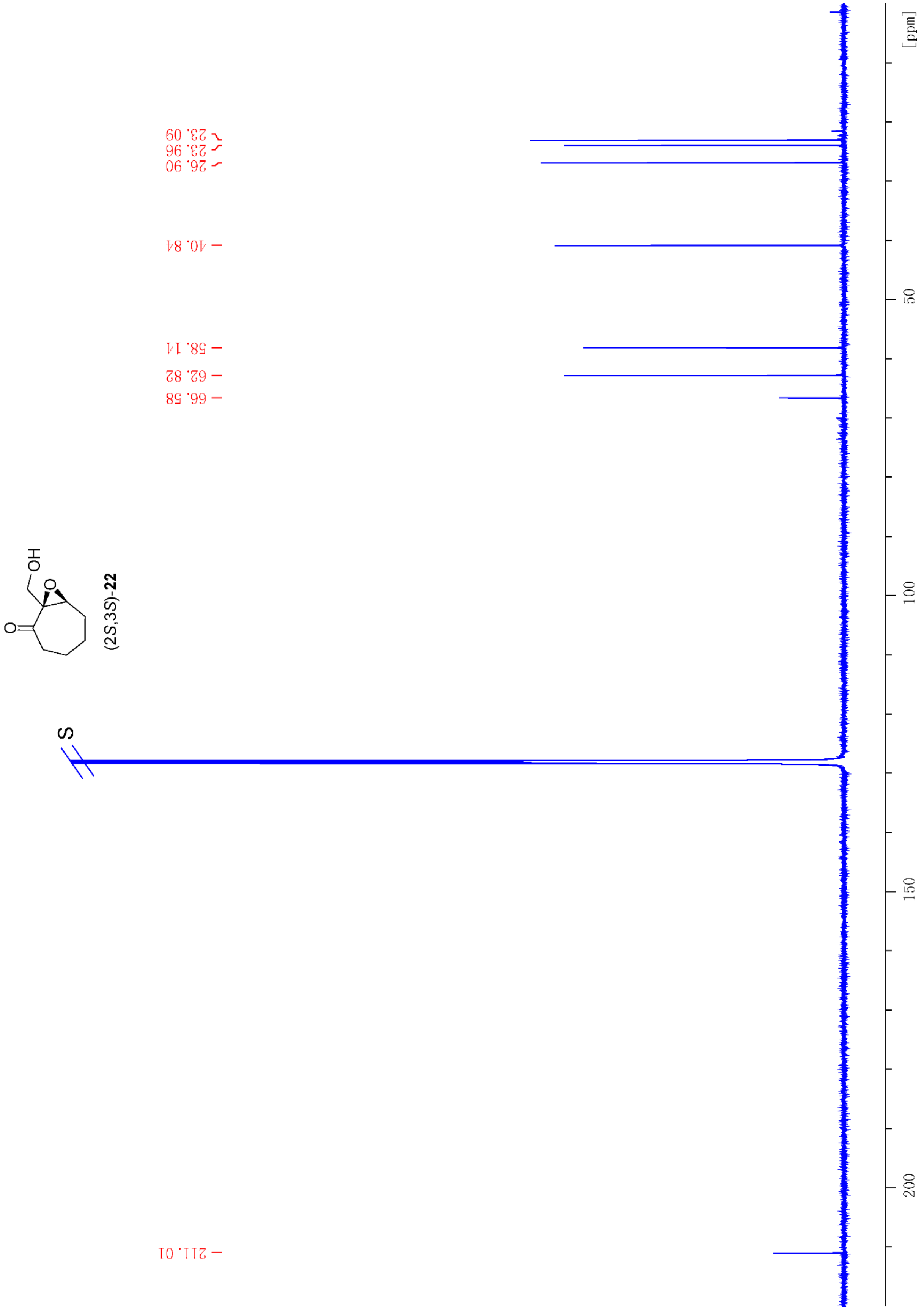

Figure S40: **^{13}C-NMR spectrum (126 MHz, C_6D_6) of (2*S*,3*S*)-**22. S indicates solvent peak.

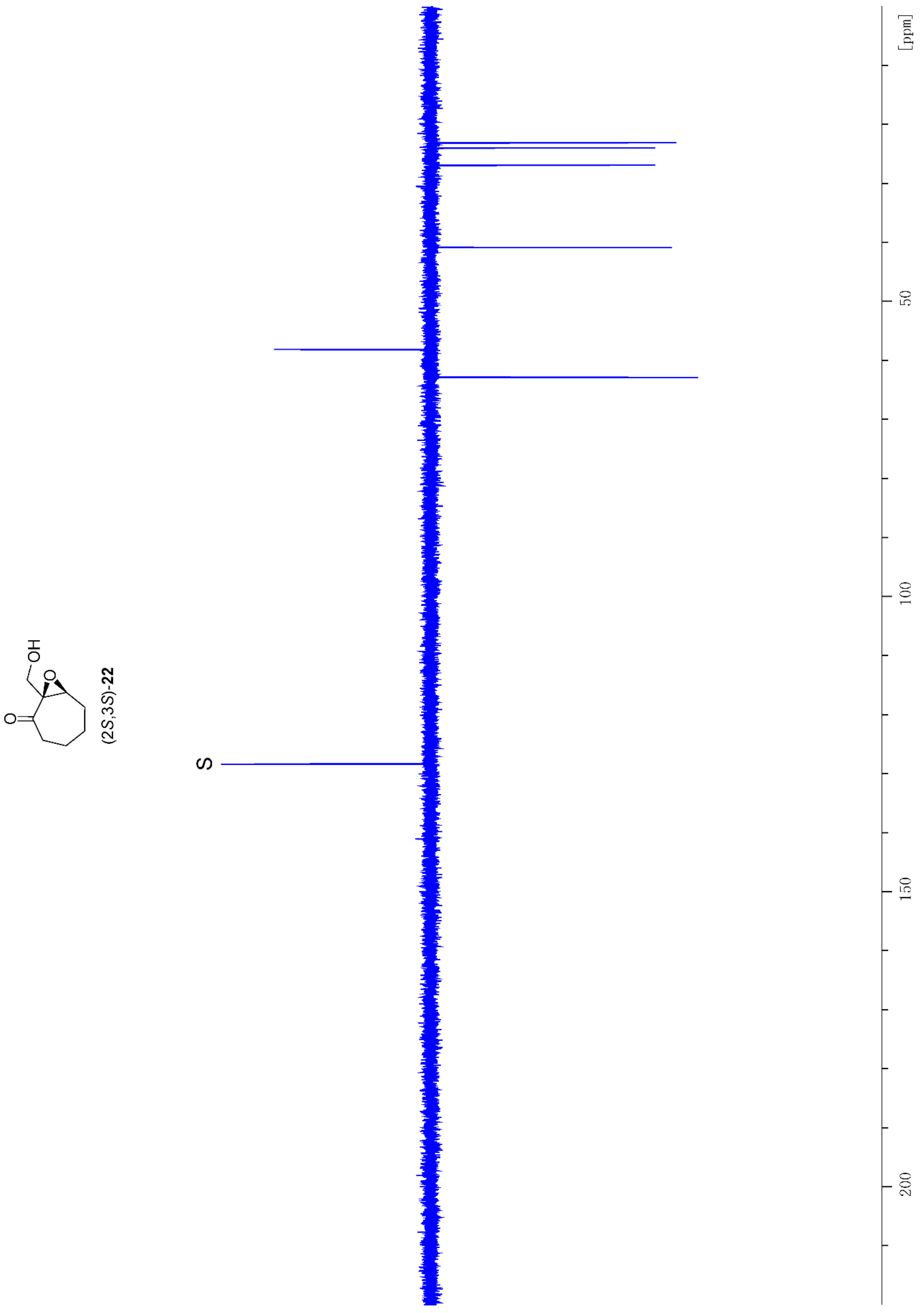

Figure S41: DEPT spectrum (126 MHz, C_6D_6) of (2*S*,3*S*)-22. S indicates solvent peak.

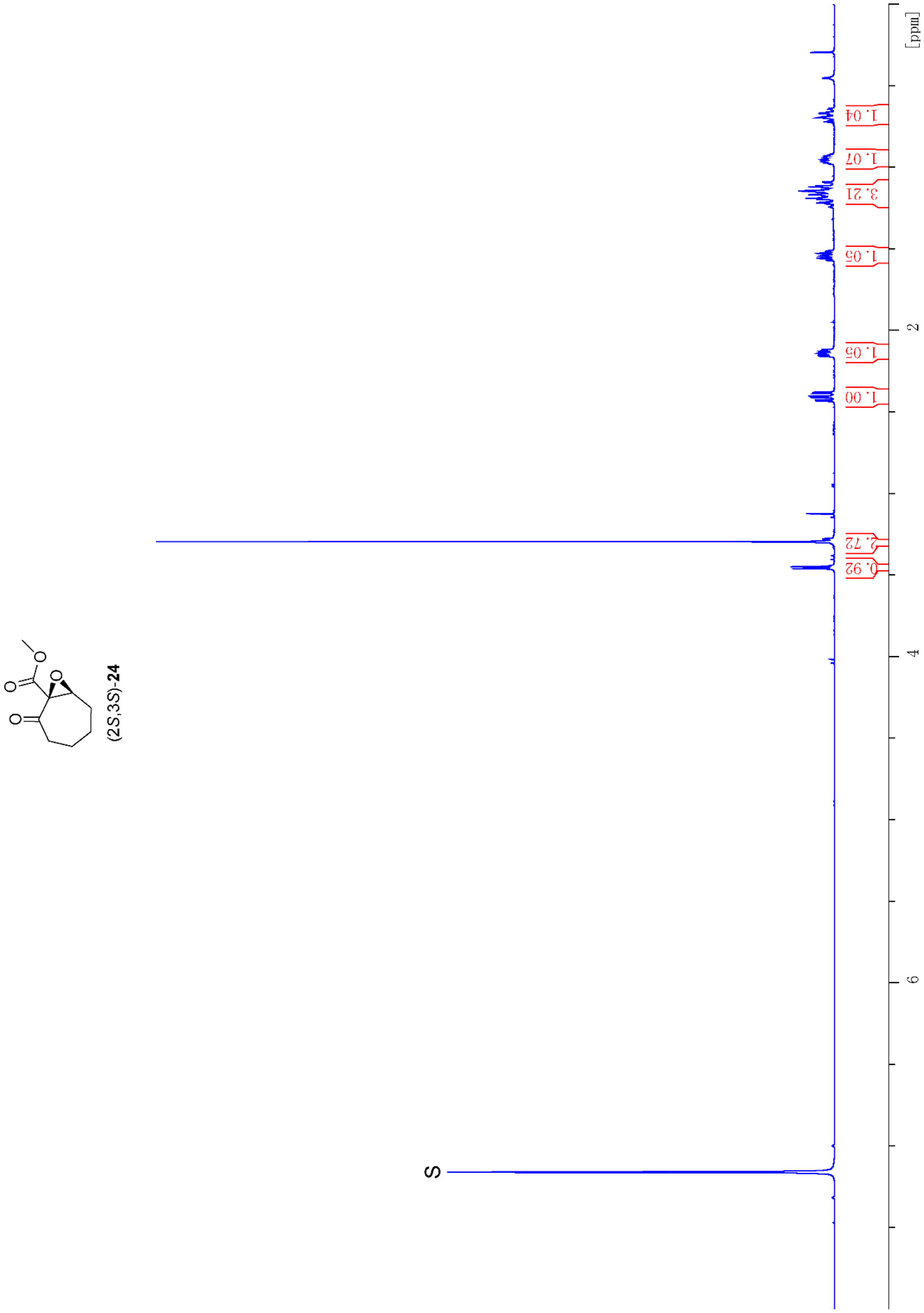

Figure S42: ^{1}H-NMR spectrum (500 MHz, C_6D_6) of (2*S*,3*S*)-24. S indicates solvent peak.

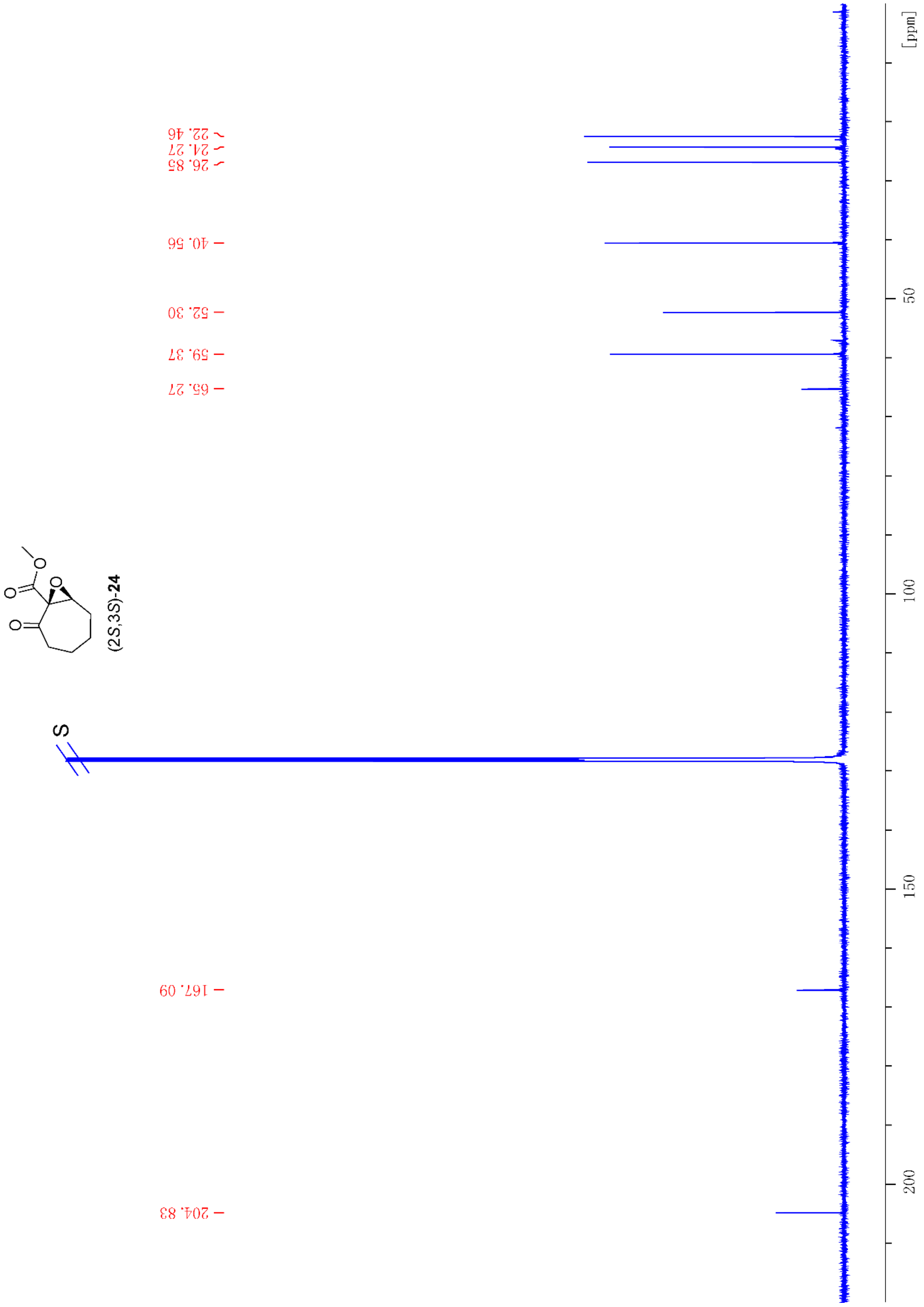

Figure S43: ^{13}C-NMR spectrum (126 MHz, C_6D_6) of (2*S*,3*S*)-24. S indicates solvent peak.

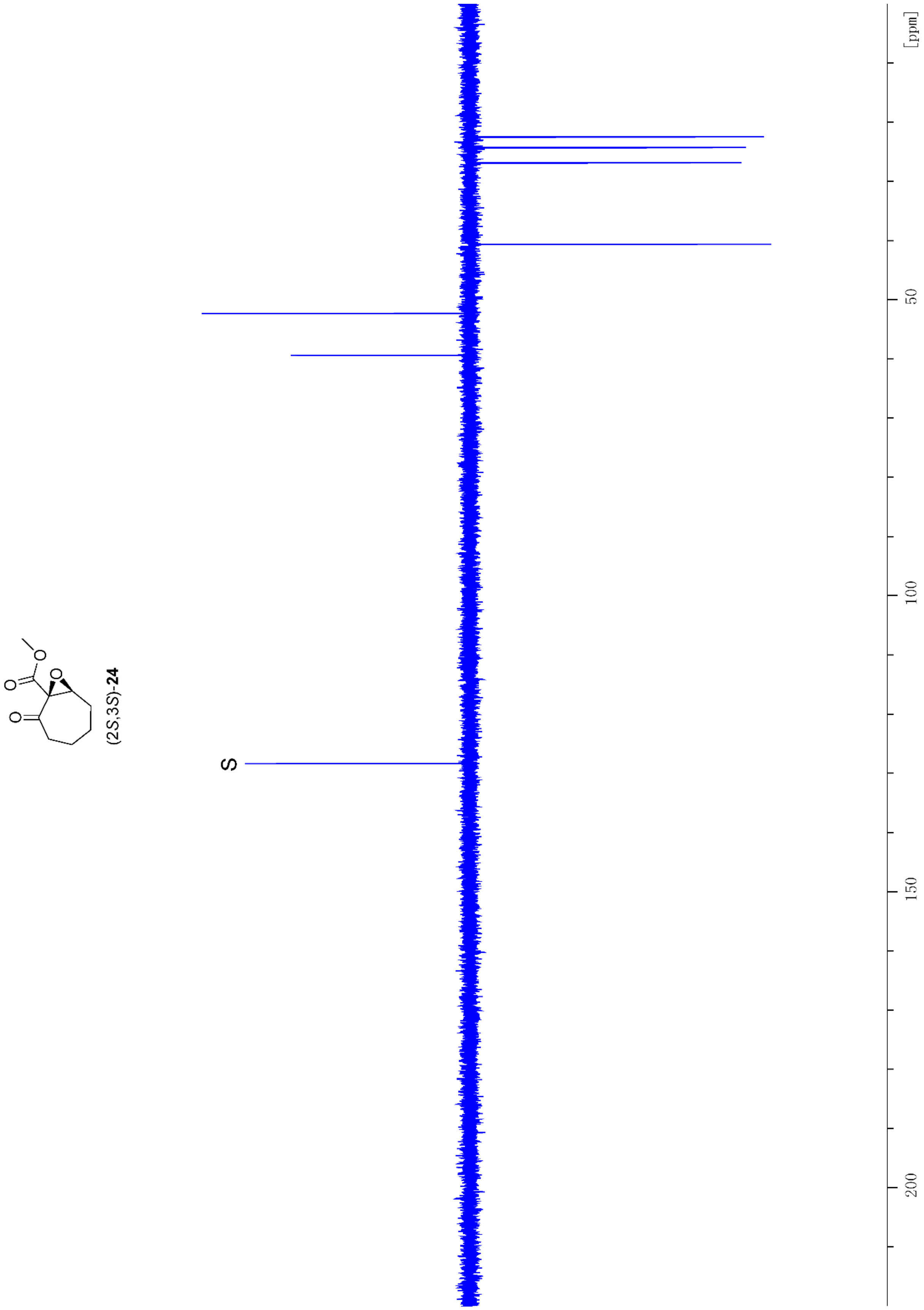

Figure S44: DEPT spectrum (126 MHz, C_6D_6) of (2*S*,3*S*)-24. S indicates solvent peak.

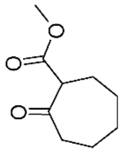

S4

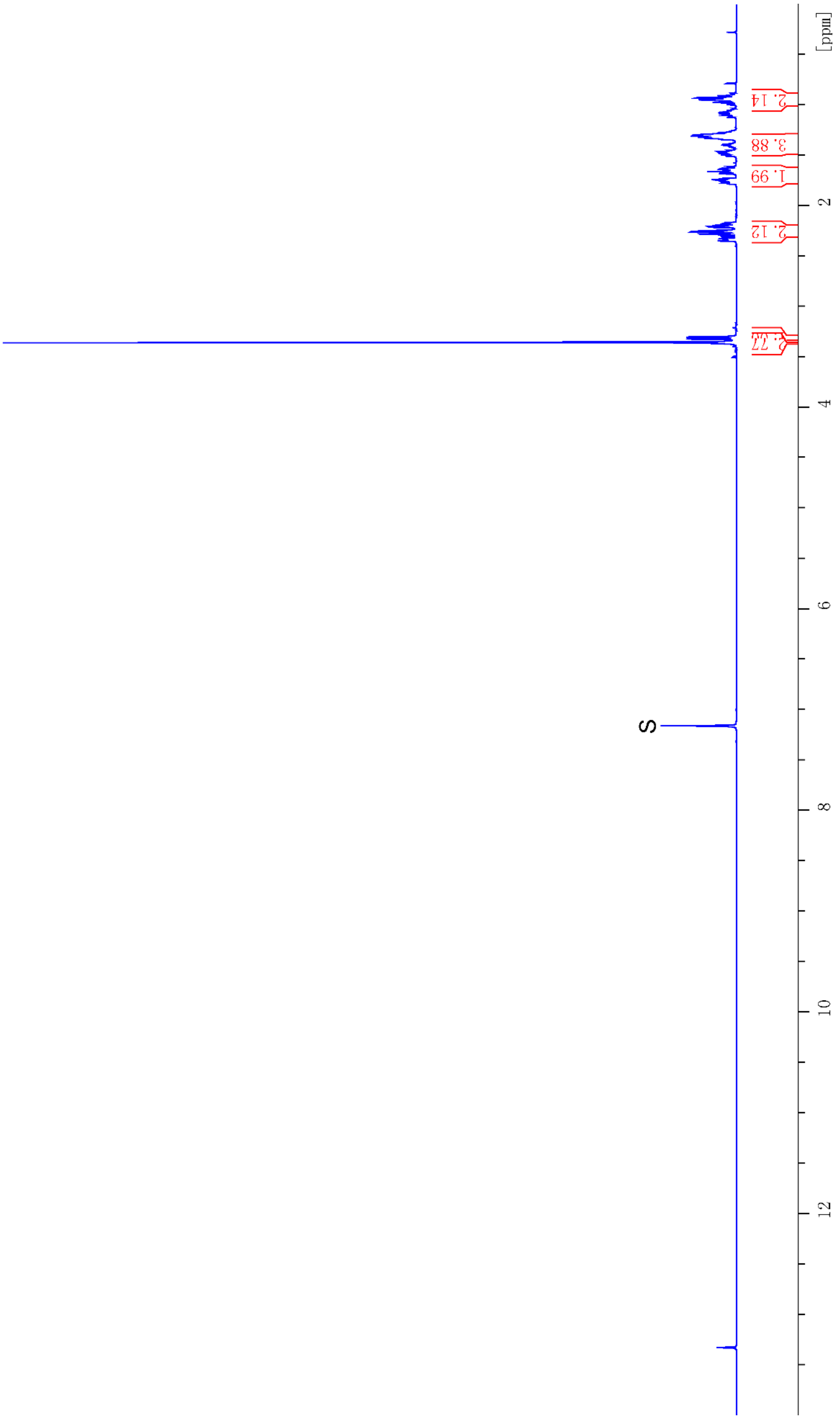

Figure S45: ^{1}H-NMR spectrum (500 MHz, C_6D_6) of S4. S indicates solvent peak.

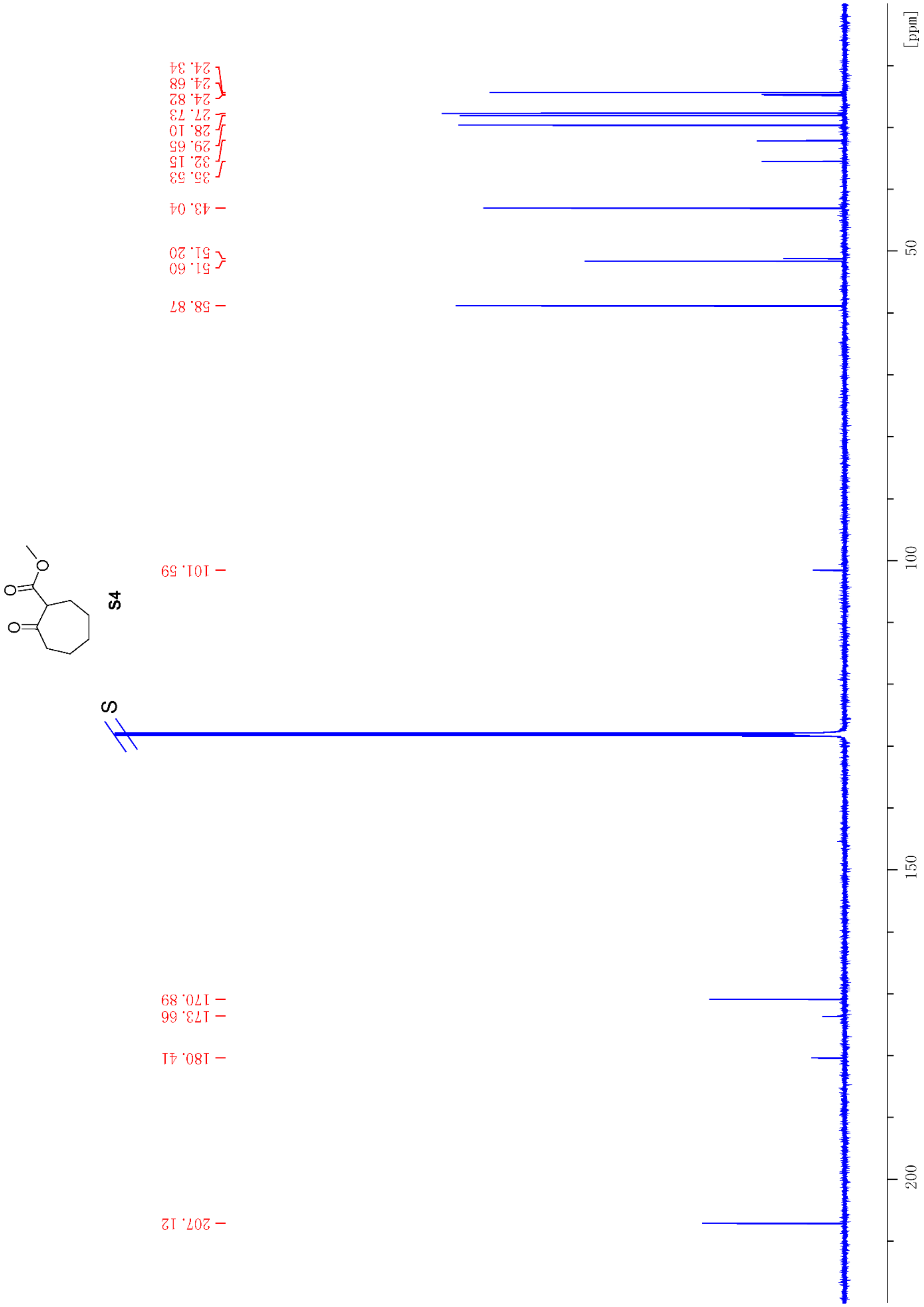

Figure S46: ^{13}C-NMR spectrum (126 MHz, C_6D_6) of S4. S indicates solvent peak.

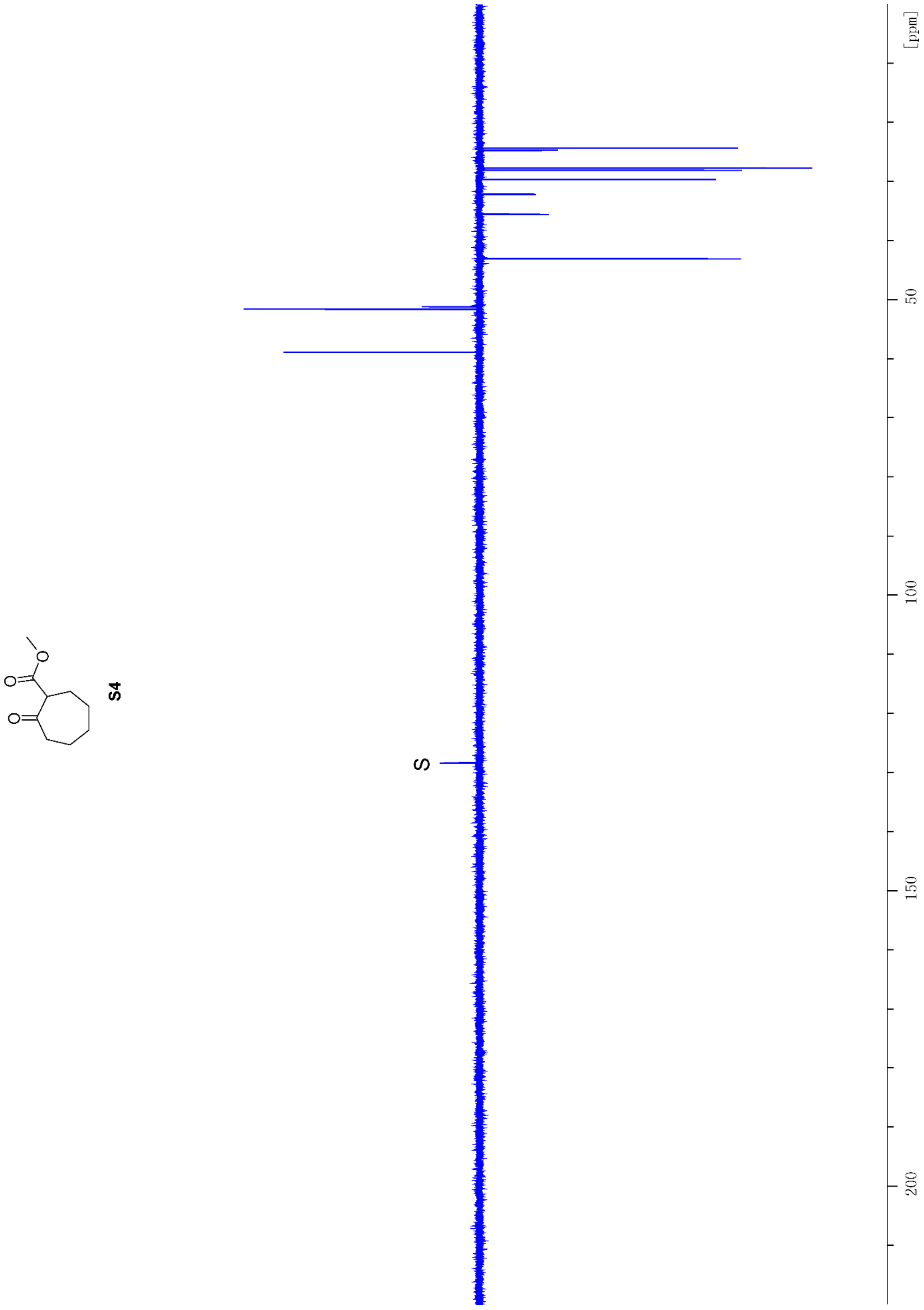

Figure S47: DEPT spectrum (126 MHz, C_6D_6) of S4. S indicates solvent peak.

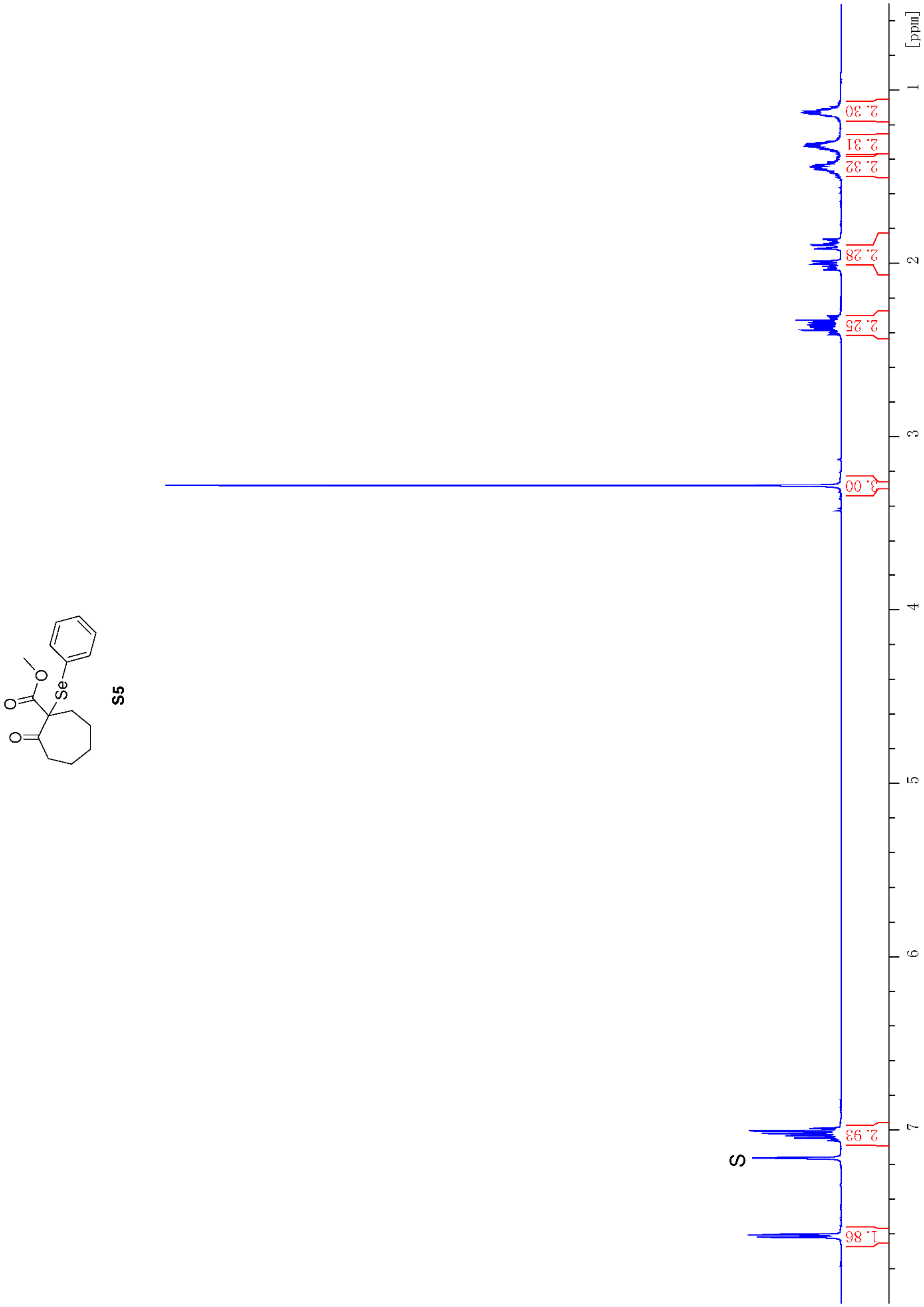

Figure S48: ^{1}H-NMR spectrum (500 MHz, C_6D_6) of S5. S indicates solvent peak.

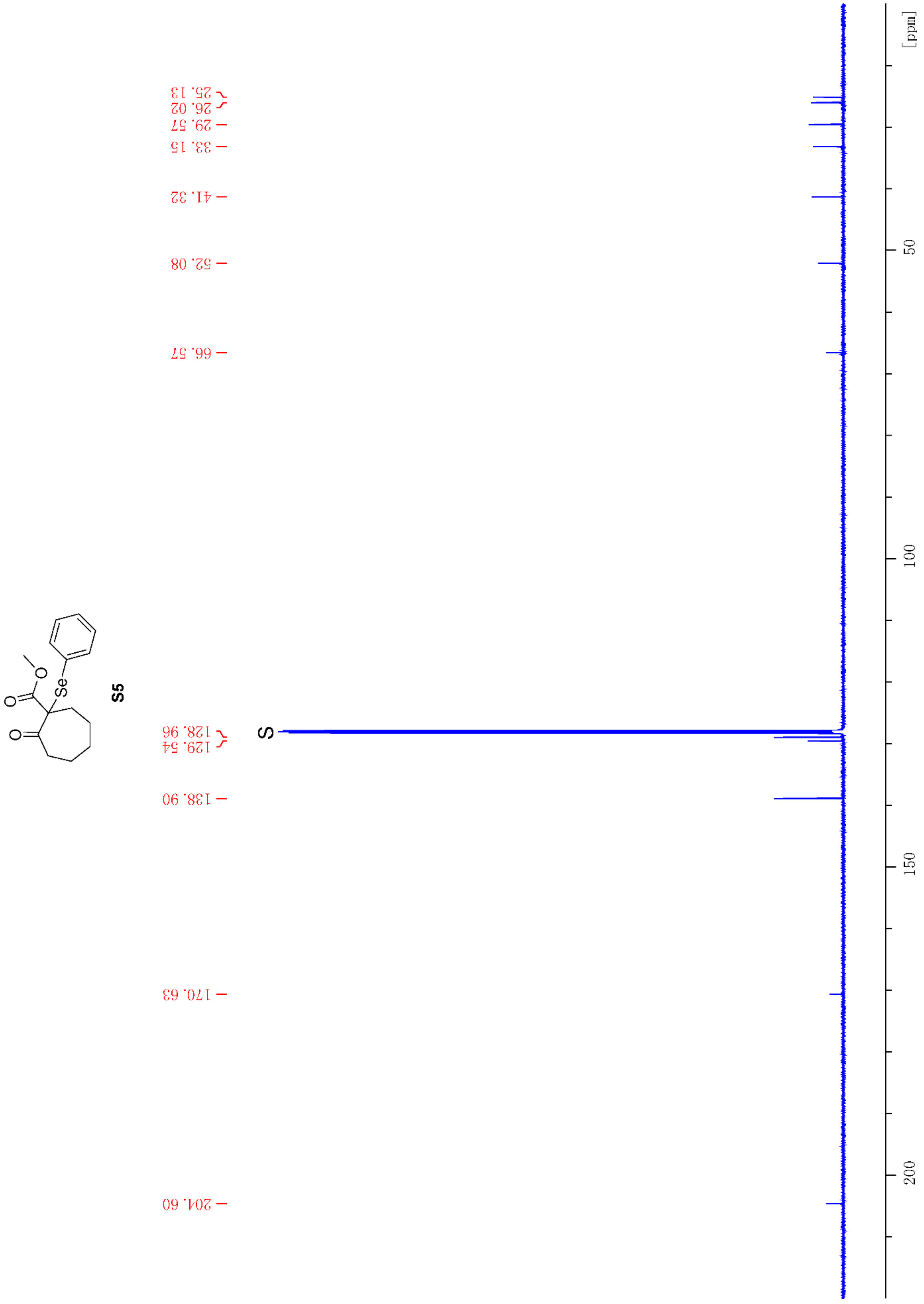

Figure S49: ^{13}C-NMR spectrum (126 MHz, C_6D_6) of S5. S indicates solvent peak.

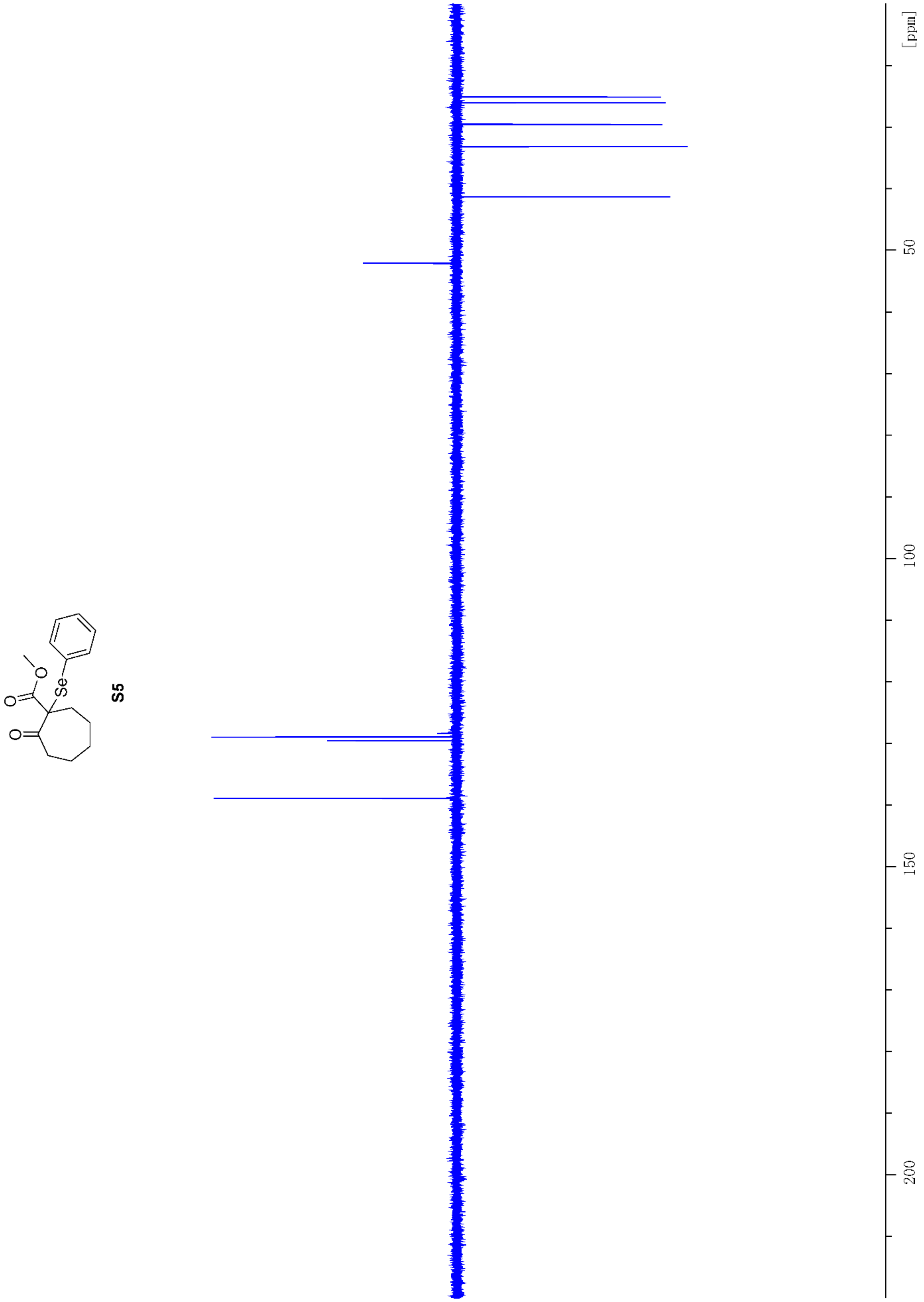

Figure S50: DEPT spectrum (126 MHz, C_6D_6) of S5.

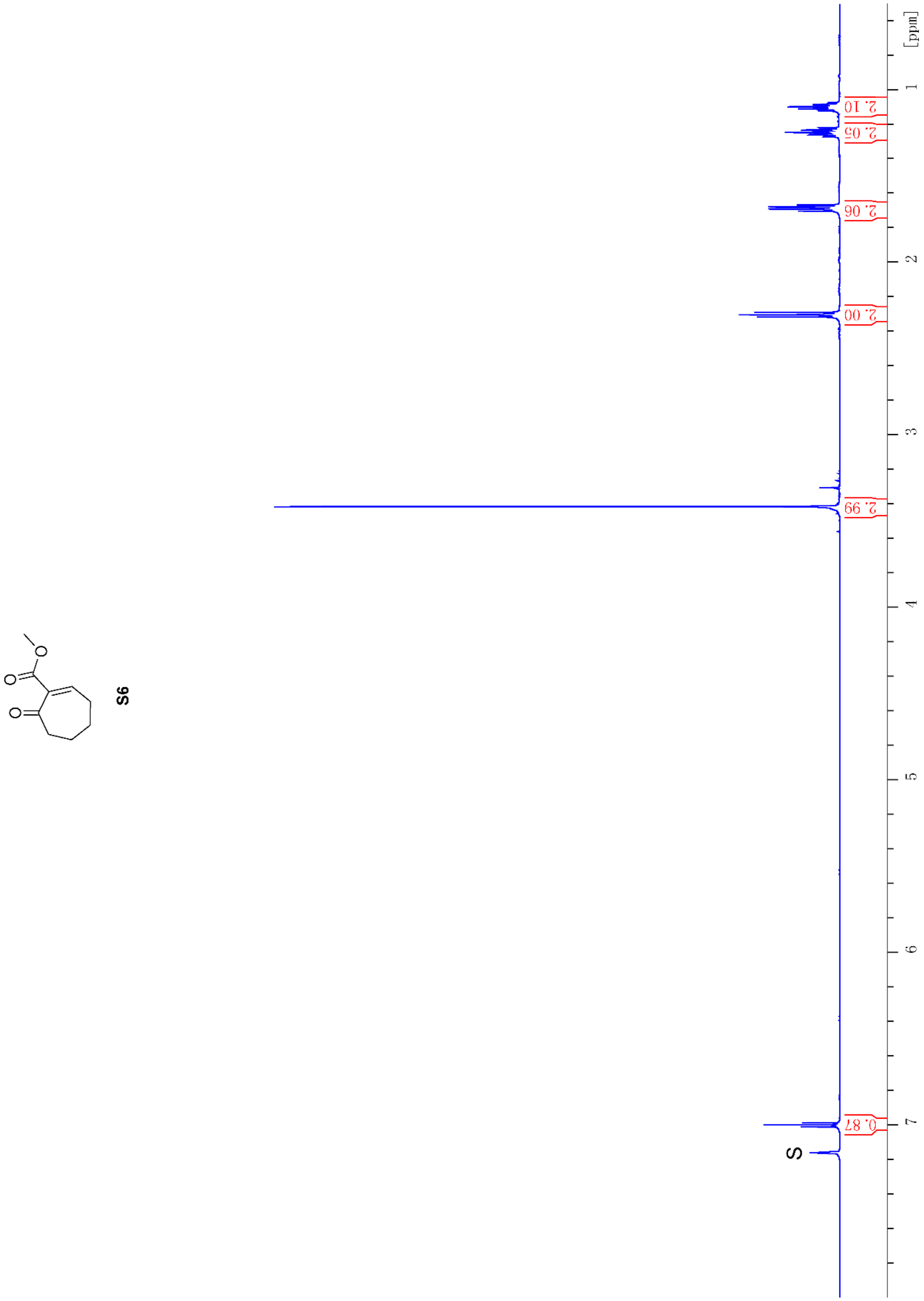

Figure S51: ^{1}H-NMR spectrum (500 MHz, C_6D_6) of S6. S indicates solvent peak.

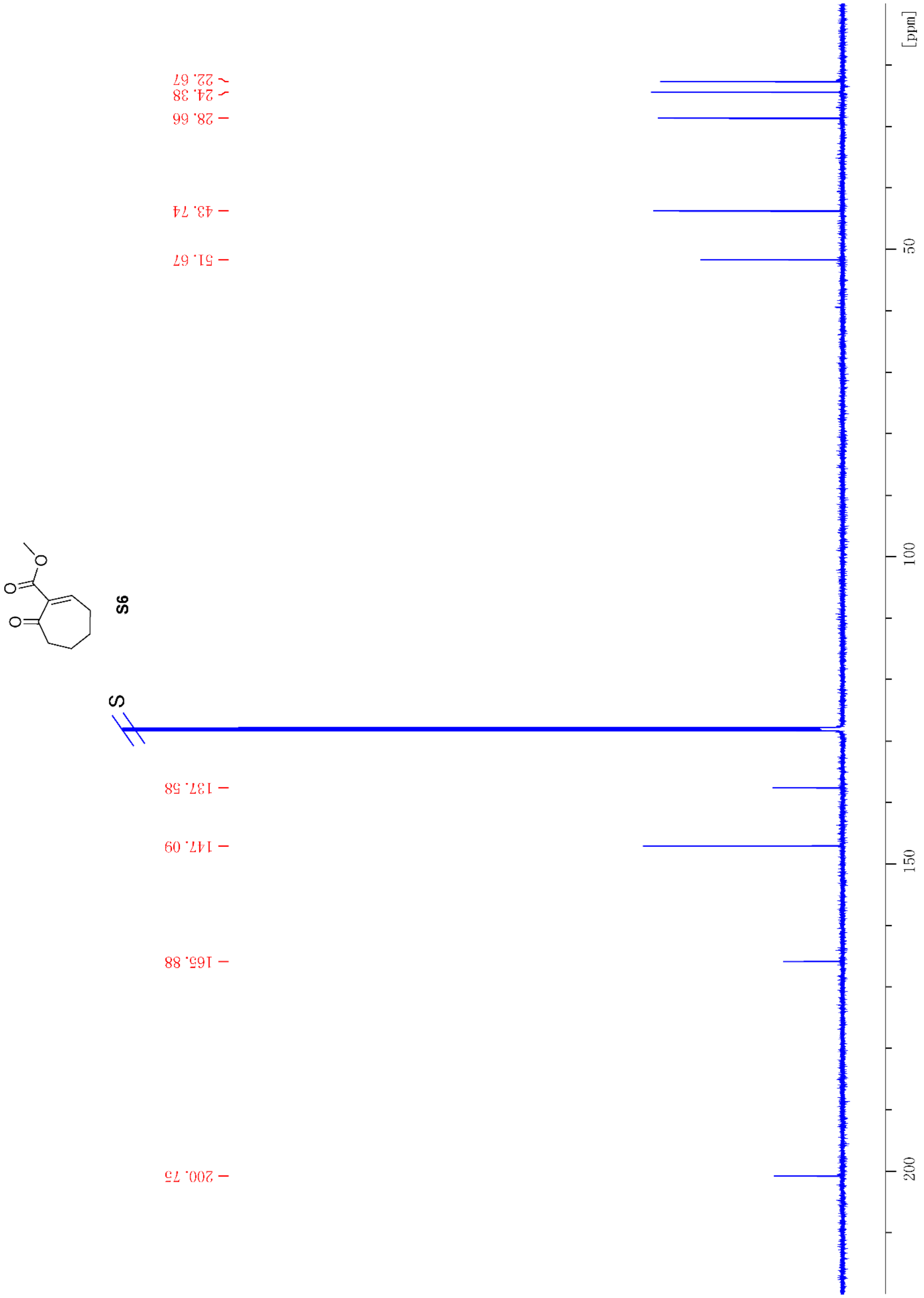

Figure S52: ^{13}C-NMR spectrum (126 MHz, C_6D_6) of S6. S indicates solvent peak.

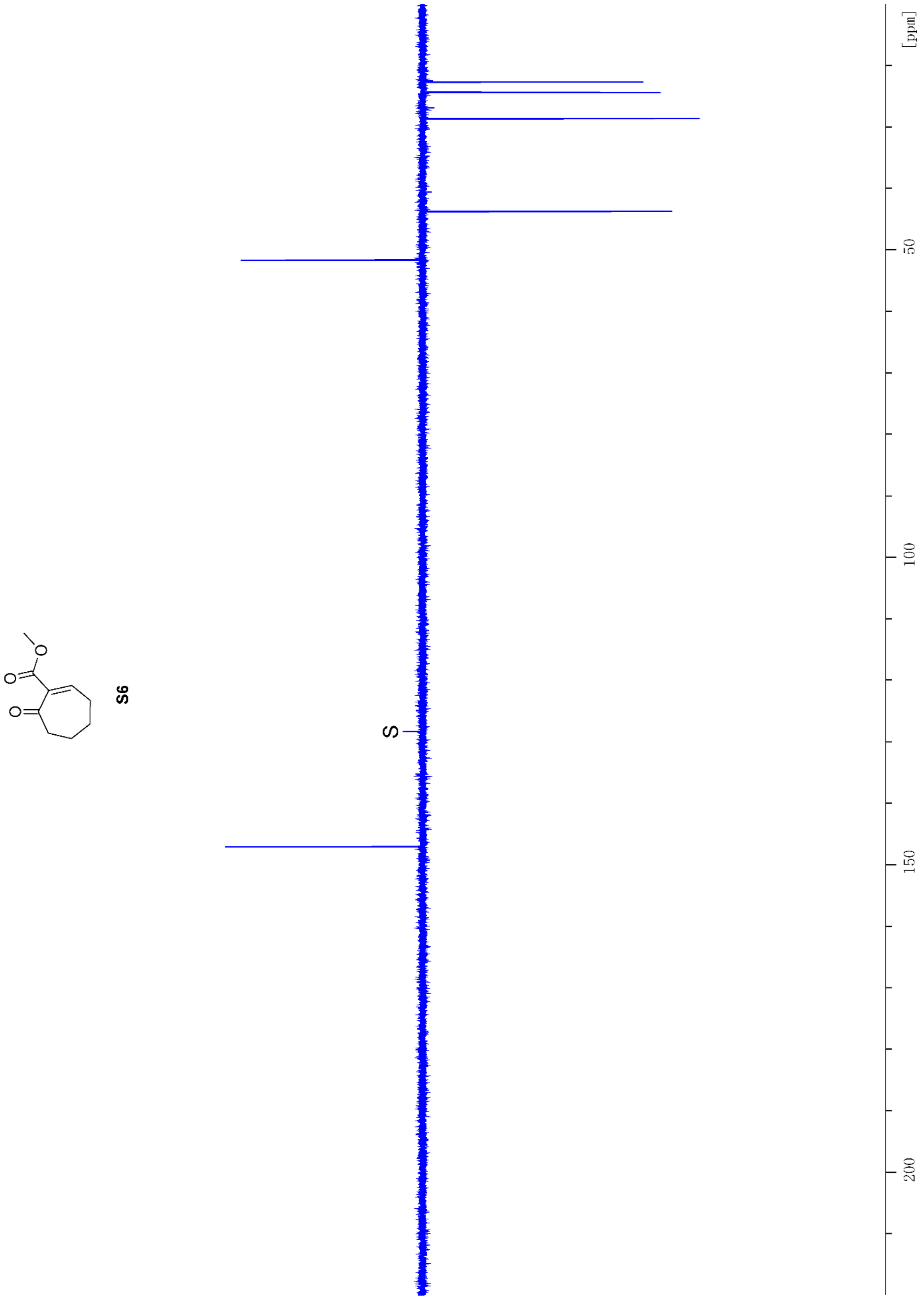

Figure S53: DEPT spectrum (126 MHz, C_6D_6) of S6. S indicates solvent peak.

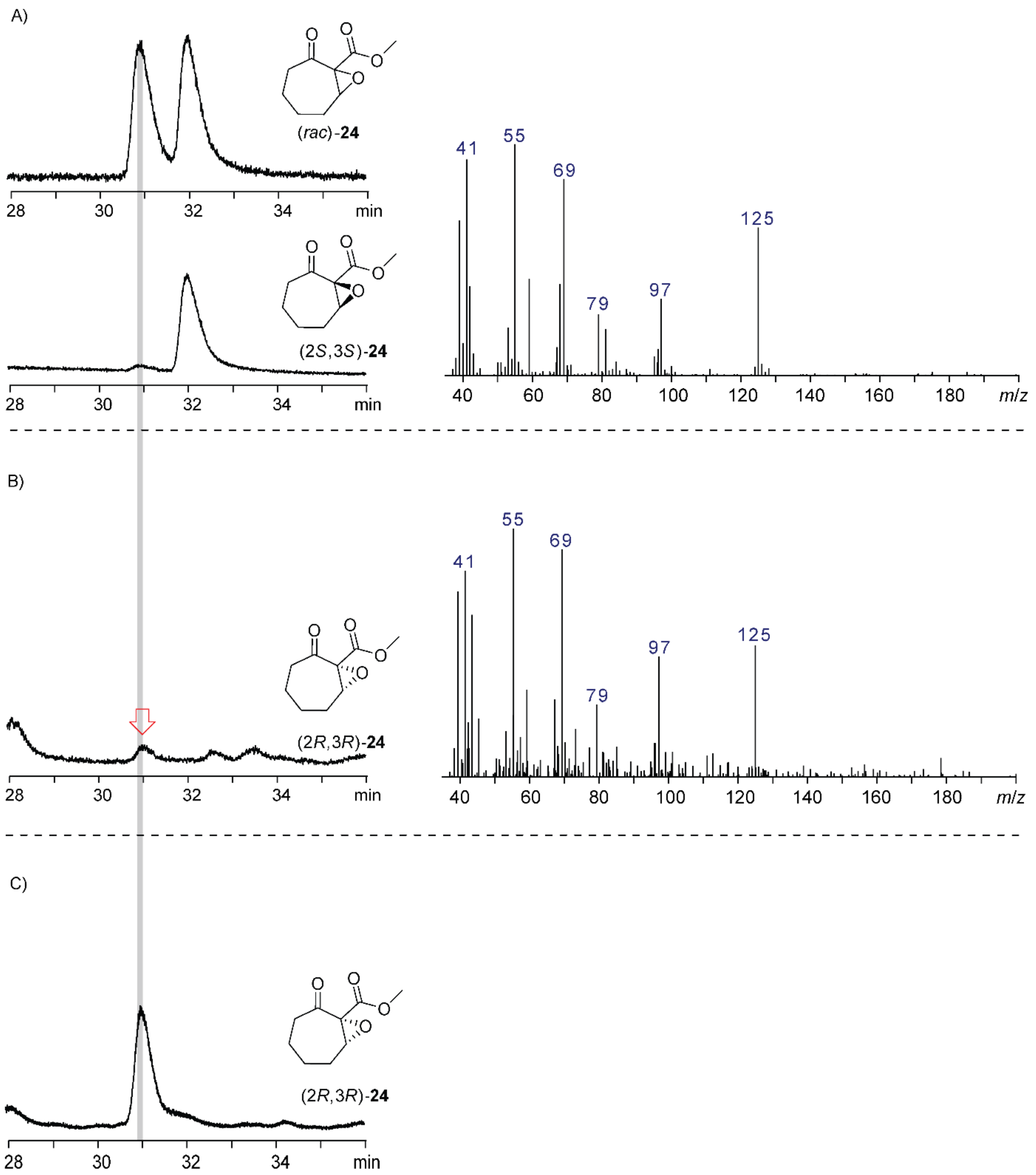

Figure S54: GC/MS analysis using a chiral Cyclosil-B capillary column. A, Total ion chromatograms of synthetic (*rac*)-**24** and (2*S*,3*S*)-**24** (*left*) and EI-MS spectrum of **24** (*right*). **B**, Total ion chromatogram of the derivatization product obtained from enzymatically generated **18** (left). The red arrow indicates the peak that has the same retention time as (2*R*,3*R*)-**24** in the racemate and the same mass spectrum as compound **24** (right). **C**, To enhance the peak intensity, the same sample as in **B** was analyzed by selected ion monitoring (SIM, *m*/*z* = 125).

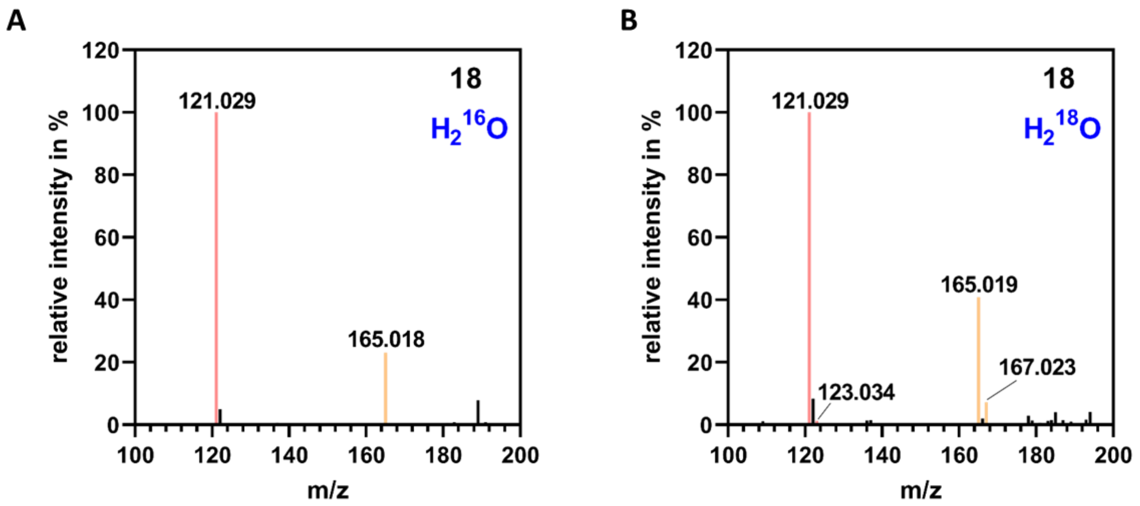

Figure S55: LC-HRMS analysis of $H_2^{18}O$ labeling assays of compound 18 **in negative ion mode.** Isotope labeling assay of compound **18** was performed by mixing compound **7** (250 µM) and PaaZ-E256Q (5 µM) in 50 mM Tris pH 8 in the presence of 50 % $H_2^{18}O$ (v/v) and incubating the reaction at 30 °C for 10 min. Then, TdaE^{Pi} (3 µM) was added and the assay mixture was incubated at 30 °C and 900 rpm for another 30 min. Reactions were quenched and extracted with EtOAc + 1 % FA and the organic layers were concentrated in the speed-vac for 5-20 min. After purification of (labeled) **18** by RP-HPLC, samples were analyzed by LC-HRMS. **A** and **B,** LC-HRMS analysis of compound **18** exposed to $H_2^{16}O$ only (**A,** control) and to 50 % $H_2^{18}O$ (v/v; **B**), indicating that the two oxygen atoms incorporated by TdaE^{Pi} derive from O_2 rather than from $H_2^{18}O$. Note that the small amount of ^{18}O-labeled **18** in **B** resulted from the activity of PaaZ-ECH during formation of **4** (see also Figure S56, isotope labeling of compound **4**).

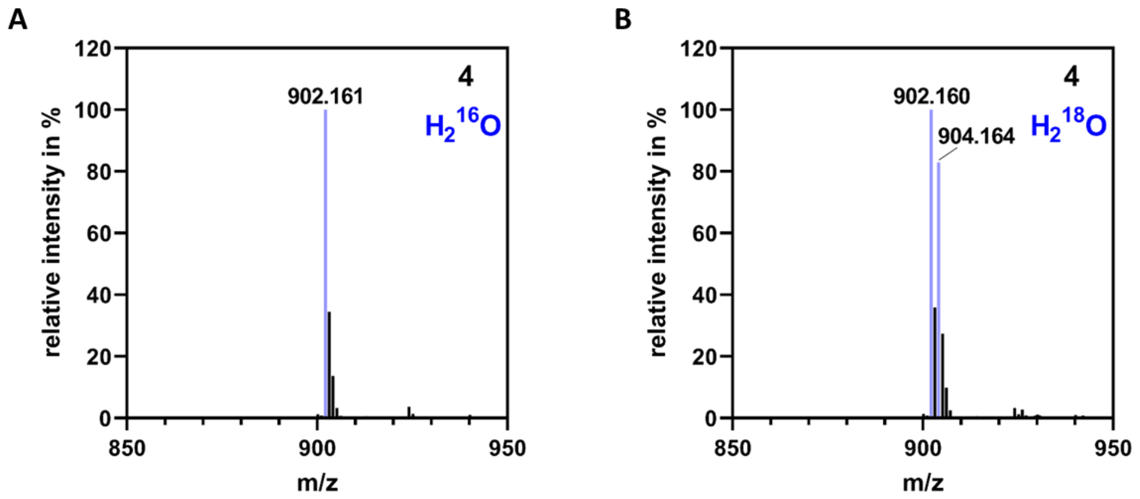

Figure S56: LC-HRMS analysis of $H_2^{18}O$ labeling of compound 4 **in positive ion mode.** Isotope labeling assay of compound **4** was performed by mixing compound **7** (250 µM) and PaaZ-E256Q (5 µM) in 50 mM Tris pH 8 in the presence of 50 % $H_2^{18}O$ (v/v) and incubating the reaction at 30 °C for 10 min. After quenching with abs. MeOH, samples were concentrated in the speed vac for 1.5 h and analyzed by LC-HRMS. **A** and **B,** LC-HRMS analysis of compound **4** exposed to $H_2^{16}O$ only (**A,** control) and to 50 % $H_2^{18}O$ (v/v; **B**), confirming the incorporation of one ^{18}O from $H_2^{18}O$ into compound **4** by the ECH domain of PaaZ-E256Q. Note that the introduced ^{18}O-label at C3 of **4** disappeared over time when incubated in aqueous solvent via spontaneous exchange with water (by ketone hydrate formation).

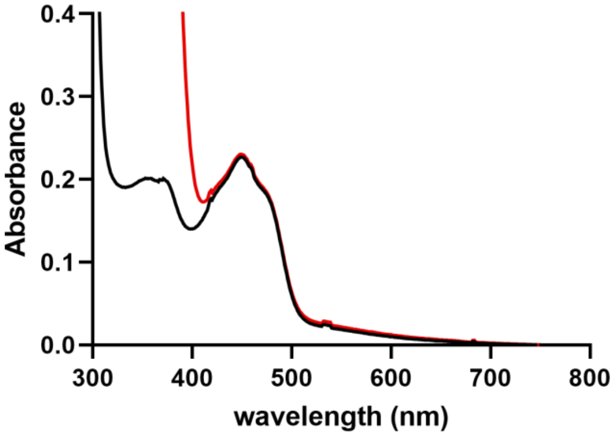

Figure S57: UV-visible absorption spectra of TdaE^{Pi} in the absence (*black*) and presence (*red*) of 1 mM NADPH. The UV-visible absorption spectra of TdaE^{Pi} recorded in 20 mM Tris, 200 mM NaCl, pH 7.4 prior to and 2 min after the addition of 1 mM NADPH are virtually identical, indicating that NADPH is not able to reduce the FAD cofactor bound to TdaE^{Pi}. The addition of compound **16** to the protein-NADPH mixture also did not have any significant effects on the spectrum.

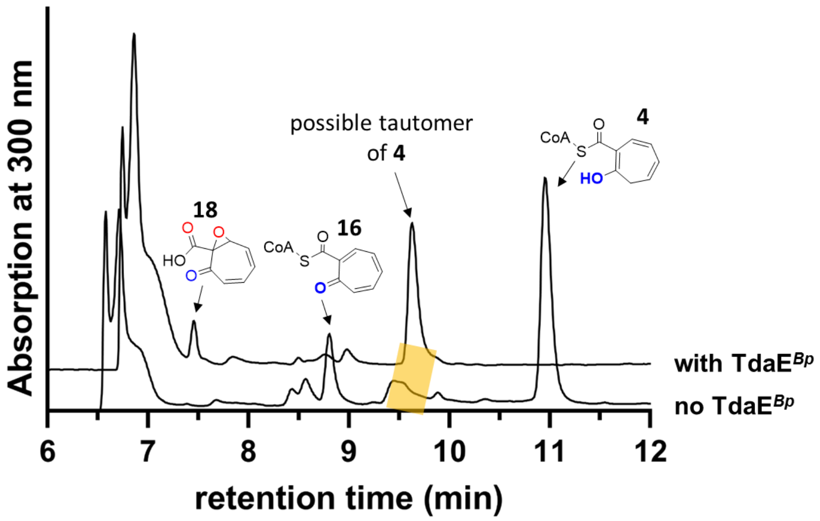

Figure S58: TdaE^{Bp}-catalyzed turnover of compound 4 **into compound** 18**.** HPLC-chromatogram of samples withdrawn from enzyme assays without (no TdaE) and with TdaE^{Bp} after 5 min. Peaks corresponding to the substrate **4**, its possible tautomer, the reaction intermediate **16** and the final product **18** are indicated. Normally, dehydrogenation occurs too fast to observe the proposed tautomerization. However, the shown assays contained TdaE^{Bp} (2-5 µM) with low FAD cofactor loading (to slow down dehydrogenation and subsequent steps), which led to the formation of a new peak, presumably the proposed C2-protonated tautomer of **4** (see Fig. 5). The same peak also formed spontaneously in much lower amounts in control samples (see Figure 3, 9.5 min peak in t(0 min)-sample). This suggests that TdaE may indeed promote the tautomerization of **4** prior to the actual dehydrogenation reaction.

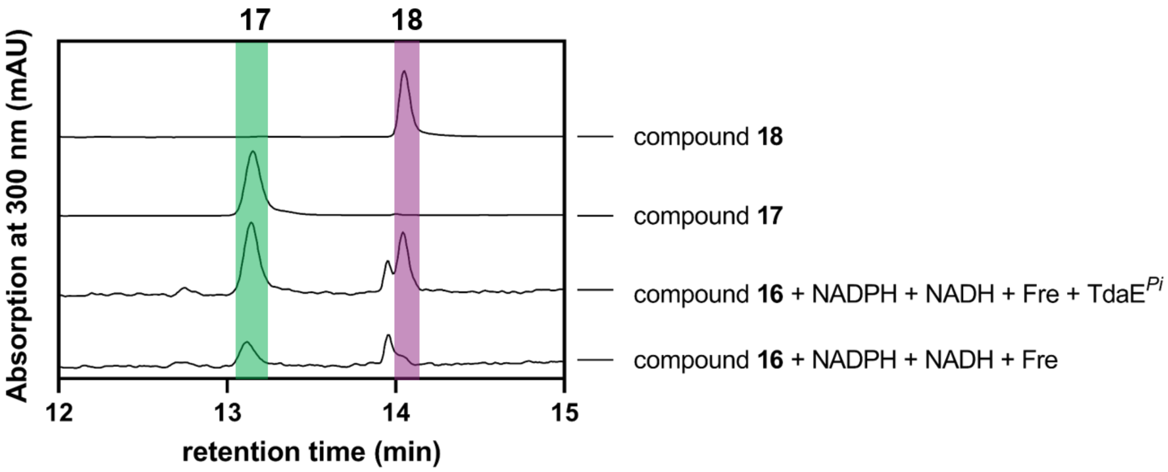

Figure S59: TdaE^{Pi} catalyzed turnover of compound 16 **into compound** 18 **in the presence of NADH-dependent flavin-reductase Fre.** The *purple* line highlights compound **18** and the *green* line compound **17**. The upper lines are two standard compounds of **17** and **18**, respectively. RP-HPLC chromatograms at 300 nm showed that compound **16** can be converted into compound **18** by TdaE^{Pi} when FAD_{red} is proffered by Fre (that normally arises from the TdaE-FAD_{ox} catalyzed oxidation of **4** into **16**). In this assay set-up, **17** also accumulated, which may be due to the reduction of FAD_{N5O} by the added flavin reductase, preventing the second oxygenation step to yield the final product **18**.

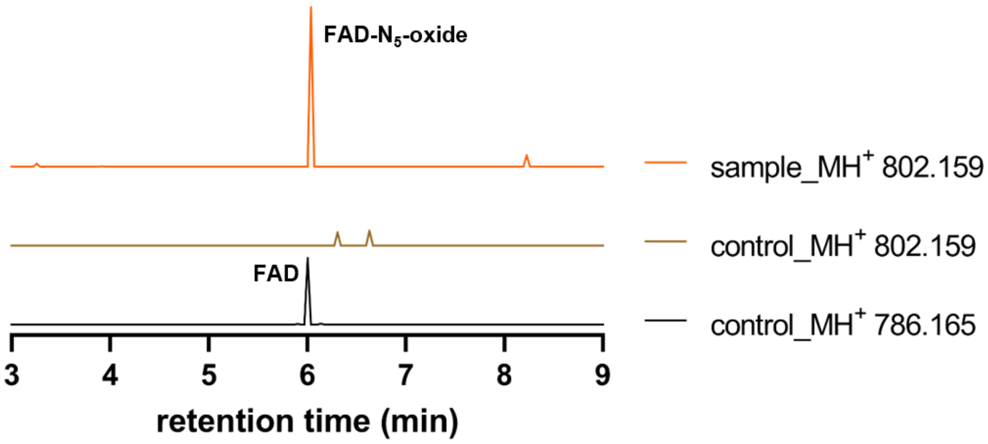

Figure S60: LC-HRMS analysis of FAD-N5-oxide formation by TdaE*Pi* upon 4 **conversion into** 18**.** After *in situ* production of compound **4** from **5**, either 20 µM of free FAD (control) or of TdaE*Pi* with bound FAD (20 µM cofactor concentration; sample) were added to the substrate mixture. Then, assays were incubated at 10 °C for 10 s to trap the putatively formed FAD-N5-oxide before quenching and extracting the samples with EtOAc + 1 % FA. Finally, the organic layers were concentrated in the speed-vac and samples were analyzed by LC-HRMS. Shown are extracted ion chromatograms (EICs) for MH^+ 786.165 of FAD (*black* line) and for MH^+ 802.159 of FAD_{N5O} (*brown* and *orange* lines), indicating the formation of FAD_{N5O} only upon **4** turnover by TdaE*Pi*.

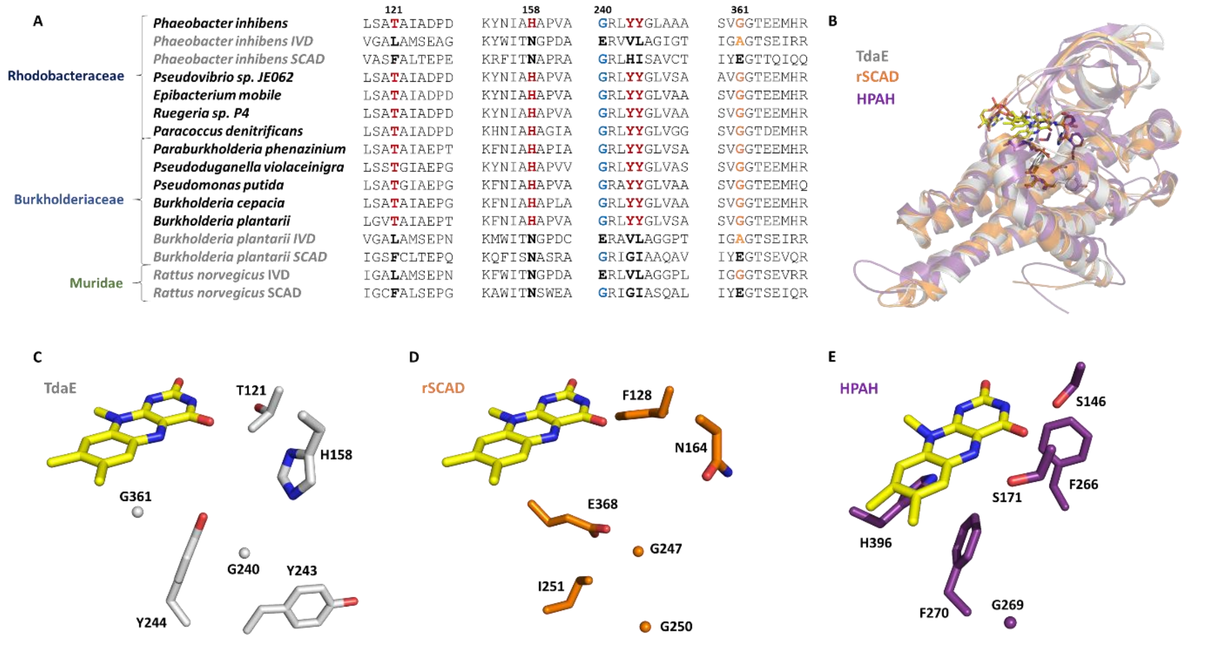

Figure S61: Sequence and structural comparison of TdaE from *Phaeobacter inhibens* (and predicted functional homologues) with a related classical acyl-CoA dehydrogenase and a class D FPMO. A, Structure based multiple sequence alignment of TdaE functional homologs (shown in bold) and classical ACADs (shown in light *grey*) from *P. inhibens*, *B. plantarii* and *Rattus norvegicus* (short-chain acyl-CoA dehydrogenase (SCAD) and isovaleryl-CoA dehydrogenase (IVD), respectively). The sequence alignment (for full sequence alignment see Figure S62 below) clearly demonstrates the strong sequence conservation among TdaE homologs from different species and highlights the differences in the (putative) catalytic residues present in the two different enzyme families. **B**, Overlay of the homology model of TdaE with the crystal structures of rat short-chain acyl-CoA dehydrogenase (rSCAD) and the microbial group D FPMO *p*-hydroxyphenylacetate 3-hydroxylase (HPAH), showing the conserved overall fold of the three proteins. **C-E**, Close-up view of the active sites of TdaE (**C**, *grey;* homology model), rSCAD (**D**, *orange*; PDB-ID: 1jqi) and HPAH (E, *purple*; PDB-ID: 2jbt) with the FAD cofactor (*yellow*) and (putative) catalytically important amino acid residues shown as sticks.

```
                                  1
Phaeobacter inhibens              -----MQLNW TAEQQQTRTA FAAIGKMADP DELHL-GRRA FD--------
Phaeobacter inhibens IVD          -----MFNA- ---------- ---------- ---------- ----SMTFDL
Phaeobacter inhibens SCAD         ---------- ---------- ---------- ---------- ------MSLD
Pseudovibrio sp. JE062            -----MDLQW NERQQALRAK YAQIGSQVDH SALHA-GRQA FD--------
Epibacterium mobile               -----MDLSW TSRQLSIRAE FASLGSRTDR DELRL-GRRA FD--------
Ruegeria sp. P4                   -----MDLSW TSRQLSIRAE FASLGSRTDR DELRL-GRRA FD--------
Paracoccus denitrificans          -----MQMHW NEARAAIRAE YARIGQS--- EAGEP-PPGA FD--------
Paraburkholderia phenazinium      -----MQFDW NTEERVLHER FRQIGAEIAR QRSGA-P-EG FD--------
Pseudoduganella violaceinigra     -----MMLQW TQEQLALRER YAALGAQLA- ARTG--QPEG FD--------
Pseudomonas putida                -----MKLSW TPQQLALLST YRAIGVELAE ARRS--AAPG FD--------
Burkholderia cepacia              -----MPFDW TQEQHATLAR FRDIGTEIAA AERDARAPAG FD--------
Burkholderia plantarii            -----MQLGW NDEERELHDR YRKLGAEIAQ ARQGA-AIDA FD--------
Burkholderia plantarii IVD        ---------- ---------- ---------- ---------M SNLPGVQFML
Burkholderia plantarii SCAD       ---------- ---------- ---------- ---------- -----MDALY
Rattus norvegicus IVD             -----MATAV RLLGR--RVS SWRLRPLPSP LAVPQRAHSM LPVDDDINGL
Rattus norvegicus SCAD            MAAALLARAG GSLGRALRAR DWR------- -----RLHTV Y----QSVEL

                                                                       37
Phaeobacter inhibens              ---------- ---------- ---------- -----QRTWD CLTAAGLWRM
Phaeobacter inhibens IVD          GEDVNALRDM VHRWAQERVR PMAQEIDQKN EFP--AELWQ EMGELGLLGI
Phaeobacter inhibens SCAD         PETLSQFLDM LDRFVRERLV PNEERVADDD AIPD--DLVH EIRDMGLFGL
Pseudovibrio sp. JE062            ---------- ---------- ---------- -----QDTWD RLLEAGLWKI
Epibacterium mobile               ---------- ---------- ---------- -----QQTWD QLGGAGLWQM
Ruegeria sp. P4                   ---------- ---------- ---------- -----QQTWD QLGGAGLWQM
Paracoccus denitrificans          ---------- ---------- ---------- -----RRRWD RLVQAGLWRM
Paraburkholderia phenazinium      ---------- ---------- ---------- -----HAGWR RLGEEGLWRL
Pseudoduganella violaceinigra     ---------- ---------- ---------- -----RDGWR QLGDAGLWRL
Pseudomonas putida                ---------- ---------- ---------- -----HYGWQ RLCDAGLWKL
Burkholderia cepacia              ---------- ---------- ---------- -----AAGWT RLGQEGLWDM
Burkholderia plantarii            ---------- ---------- ---------- -----HDGWR KLKDAGLWRI
Burkholderia plantarii IVD        GEDIEMLRDA IATFAAKEIA PRAAEIDRTD QFP--MDLWR KFGELGVLGM
Burkholderia plantarii SCAD       TEDQRMIRDT VRDFSTEVLA PNAAQWDRDG VLPD--SVVA QLGELGLLGM
Rattus norvegicus IVD             NEEQKQLRHT ISKFVQENLA PKAQEIDQSN DFKNLREFWK QLGSLGVLGI
Rattus norvegicus SCAD            PETHQMLRQT CRDFAEKELV PIAAQLDKEH LFPT--SQVK KMGELGLLAM

                                  52
Phaeobacter inhibens              IVPREFGGDG RDWWGFTAAL EGLAATLRTP GMLLSVIAQA GMVRALDLYG
Phaeobacter inhibens IVD          TVPEEFGGAG MSYLAHTVAV EEIARASASV SLSYGAHSNL -CVNQIKLNG
Phaeobacter inhibens SCAD         SIPEEYGGLG LTMHEEVQAA FVLGQTSPAF RSLVGTNNGI -GSQGLIIDG
Pseudovibrio sp. JE062            IVPSEYGGEG TDWWDFTAAL EGLASSVRTP GILLSVIAQA GMVHALNLFG
Epibacterium mobile               MVPTDFGGTG VDWWDVTAAL EGLASTIRTP GLLLSVIAQA GMAYALDLFG
Ruegeria sp. P4                   MVPTDFGGTG VDWWDVTAAL EGLASTIRTP GLLLSVIAQA GMAYALDLFG
Paracoccus denitrificans          VVPPEHGGAG VDWWNFTAAL EGLASTIRHP GLVLSVIGQA GMVRALGLYG
Paraburkholderia phenazinium      AIPRAYGGDE RGWWSFTAAL DGLAQGIRTP ALLLSAIAQA GMIRAFSEYG
Pseudoduganella violaceinigra     IVPANFGGAG TDWWAFTAAL EGLASSIRTP ELLLSVIAQA GLVRALVHYG
Pseudomonas putida                VIPLEYGGLG EDWWGFSAAL EGLASSIRTP ELLLSVIAQA GMVRALMLYG
Burkholderia cepacia              IVPETYGGDG HGWWHFSAAL EGLASTIRRP ALLLSVIAQA GMVRALERYG
Burkholderia plantarii            AIPQAFGGDE RGWWAFTAAL EGLASGIRTP ALVLSVIAQA GLIRAMTGYG
Burkholderia plantarii IVD        TVGEEYGGAN LGYTAHMVAM EEISRASASV GLSYGAHSNL -CVNQIHRNG
Burkholderia plantarii SCAD       VVPSEWEGSY TDYIAYALAV EEIAAGCASC ATLMSVQNSV -CCGPILGYG
Rattus norvegicus IVD             TAPVQYGGSG LGYLEHVLVM EEISRASAAV GLSYGAHSNL -CINQIVRNG
Rattus norvegicus SCAD            DVPEELSGAG LDYLAYSIAL EEISRGCAST GVIMSVNNSL -YLGPILKFG

                                  102                 121
Phaeobacter inhibens              SDSQKREYLG RILNGE-LSA TAIADPDTGT DVRATSSLLT PGPNETFRLS
Phaeobacter inhibens IVD          NAEQKAKYLP RLVSGEHVGA LAMSEAGAGS DVVSMSLR-A EKRNDHYRLN
Phaeobacter inhibens SCAD         TPQQKESYLP QLATGEMVAS FALTEPEAGS DAGSLRTS-A RKDGDHYILS
Pseudovibrio sp. JE062            TEAQKRDYLG RILKGK-LSA TAIADPDTGT DVRSTSSVLT PGPNETFVLN
Epibacterium mobile               TTAQKSDYFR RILRGE-LSA TAIADPDTGT DVRASSTFLS PRANETFVLN
Ruegeria sp. P4                   TTAQKSDYFR RILRGE-LSA TAIADPDTGT DVRASSTFLS PRANETFVLN
Paracoccus denitrificans          TEAQRRRYLR RILAGE-LSA TAIADPDTGT DVRATSSILA PGPNETFVLN
Paraburkholderia phenazinium      RDAQKEQYFR AILAGE-LSA TAIAEPTTGT DVRSIGTQLV P-DGDAYRLS
Pseudoduganella violaceinigra     SPAQQDKYFA AILRGD-LSS TGIAEPGTGT DVRSIETRLT P-EGDNYVLN
Pseudomonas putida                SDAQKDRYLS AILAGA-LSA TGIAEPGTGT DVRSIHSLLT P-CADGYRLT
Burkholderia cepacia              TATQQDRYFG AILRGE-LSA TAIAEPGTGT DVRSIATKLV E-HGDGYRLT
Burkholderia plantarii            SPAQREKYFG ALLRGE-LGV TAIAEPTTGT DVRSIGTVLE P-QGDGYVLS
Burkholderia plantarii IVD        SEAQKRRYLP KLISGEHVGA LAMSEPNAGS DVVSMKLR-A DKRGSHYVLN
Burkholderia plantarii SCAD       TDAQRDRWLR GLALGRMIGS FCLTEPQAGS EASNLRTR-A VLKDGRWVLD
Rattus norvegicus IVD             NEAQKEKYLP KLISGEFIGA LAMSEPNAGS DVVSMRLK-A EKKGDHYVLN
Rattus norvegicus SCAD            SSQQKQQWIT PFTNGDKIGC FALSEPGNGS DAGAASTT-A REEGDSWVLN
```

```
                                   151       158
Phaeobacter inhibens               GAKYNIAHAP VASFILVVCK LSDH---GRE GISLVLLDAD SPGLTIGAQD
Phaeobacter inhibens IVD           GNKYWITNGP DADTLVVYAK TDPDA--GSK GITAFLIEKE IKGFSTSQHF
Phaeobacter inhibens SCAD          GTKRFITNAP RAGLFTVFAR TDPSS-KTSA GVTAFLVEAD TSGLSLGPID
Pseudovibrio sp. JE062             GAKYNIAHAP VADFTLVVCK LDGH---ARD GISLILLDAG TQGLEVGEYD
Epibacterium mobile                GRKYNIAHAP VASFTLVVCK LEGH---ARD GISLVLVDQN SKGVTIGPQD
Ruegeria sp. P4                    GRKYNIAHAP VASFTLVVCK LEGH---ARD GISLVLVDQN SKGVTIGPQD
Paracoccus denitrificans           GAKHNIAHAG IATMVLIVCK LAGE---GRE GISLVLVDTD RPGLRAGPPD
Paraburkholderia phenazinium       GNKFNIAHAP IASFTLVVTK LVGT---DAD GVTLVVLDHD AAGMTLGQPD
Pseudoduganella violaceinigra      GGKYNIAHAP VVDFMMIVSR --AN---DEG AISLVLLDKD APGLRIGKAD
Pseudomonas putida                 GSKFNIAHAP VADFLLIVTR LATD---SKH NIALVIIDKD TPGLTRGEPD
Burkholderia cepacia               GSKFNIAHAP LARFILVVTR VETL---GRR NTALVIVDRD QPGMTVAAPD
Burkholderia plantarii             GDKFNIAHAP VADFSLVVAK LTGA---ARE GVTLVMLDKD APGIHAGPPD
Burkholderia plantarii IVD         GTKMWITNGP DCDTLVVYAK TEPEA--GAR GMTAFIVEKG MKGFSVAQKL
Burkholderia plantarii SCAD        GSKQFISNAS RAAVAIVFAM TDPEQ--GKR GLSAFIVPTD TPGFQVGKPE
Rattus norvegicus IVD              GNKFWITNGP DADVLVVYAK TDLTAVPASR GITAFIVEKD MPGFSTSKKL
Rattus norvegicus SCAD             GTKAWITNSW EASATVVFAS TDRSR--QNK GISAFLVPMP TPGLTLGKKE

                                   198                                          240
Phaeobacter inhibens               RKLGNLDLPT GQMRFDDVPL HYGHLLGEP- --GAGLRNLV NIVSMGRLYY
Phaeobacter inhibens IVD           DKLGMRGSNT AELVFEDVEV PFENVLGEE- --GKGVRVLM SGLDYERVVL
Phaeobacter inhibens SCAD          RKMGQKGSHT CDVILDGCRV HESAIIGGPD RLGQGFKTAM KVLDRGRLHI
Pseudovibrio sp. JE062             QKLGNLDLPT GPMKFNNIPL HYGDLLGVP- --GKGLRNLI TFVSLGRLYY
Epibacterium mobile                RKLGNLDLPT GQLTFEDVPM HYGHILGVP- --GEGLRNLV RFVSLGRLYY
Ruegeria sp. P4                    RKLGNLDLPT GQLTFEDVPL HYGHILGVP- --GEGLRNLV RFVSLGRLYY
Paracoccus denitrificans           RKLGNADLPT GRLGFDAMAL DYGDLLGEP- --GRGLGNLV NIVSLGRLYY
Paraburkholderia phenazinium       QKFGNRDLPT GPITFDNVPV RRDQILGTP- --GRGLQQLI NIISLGRLYY
Pseudoduganella violaceinigra      DKLGNRNLPT GALHFDNVPV AASQILGKP- --GKGLQQLI DIISLGRLYY
Pseudomonas putida                 DKLGNKHLPT GPLHFDNLPV PAEQVLGEP- --GRGLQQLI RIISLGRAYY
Burkholderia cepacia               RKLGLDDLPT GALHFDDCPI ARGQLLGEP- --GAGLGNLI DIISLGRLYY
Burkholderia plantarii             TKFGNRALPT GPIRYENTPV SREQILGEP- --GRGLQQLI DIISLGRLYY
Burkholderia plantarii IVD         DKLGMRGSHT GELVFQDVEV PEENVLGEV- --GSGVKVLM SGLDYERAVL
Burkholderia plantarii SCAD        KKLGIRASDT CPITLEHCEI PEENLLGAR- --GEGLKIAL SNLEGGRIGI
Rattus norvegicus IVD              DKLGMRGSNT CELVFEDCKV PAANILSQE- --SKGVYVLM SGLDLERLVL
Rattus norvegicus SCAD             DKLGIRASST ANLIFEDCRI PKENLLGEP- --GMGFKIAM QTLDMGRIGI

                                   245
Phaeobacter inhibens               GLAAAWLIEP MLTEAFDFSK KRTTFDVPII EHQYIQKKLT DIRIGSESAK
Phaeobacter inhibens IVD           AGIGTGIMAA CMDEMMPYMK ERKQFGQPIG NFQLMQGKIA DMYTAMNTAR
Phaeobacter inhibens SCAD          SAVCTGNAER LIRDSLEYAM DRKQFGEPIA EKQLVQAMLA DSRTEAFAAR
Pseudovibrio sp. JE062             GLVSAWLVEP MLKAAMSYAQ QRQTFEVPVL EFQYVQKKLT DMRISAEAAK
Epibacterium mobile                GLVAATLCEP MLAEALSYAK TRQTFGQPIV DHQYVQKKLT DMRIAADSAK
Ruegeria sp. P4                    GLVAATLCEP MLAEALSYAK TRQTFGQPIV DHQYVQKKLT DMRIAADSAK
Paracoccus denitrificans           GLVGGWLLEP ALAEALDYAQ RRQTFGVPIL DHQYVQKKLT DIRIGIETSR
Paraburkholderia phenazinium       GLVSAALVEP YLDDALDYAS ARHSFNETIA EHQYVQKRLV DVRIGIERTR
Pseudoduganella violaceinigra      GLVASHLVTP FLADAMAYAK QRISFKDTIA EHQYVQRRLT DLKIGAERGR
Pseudomonas putida                 GLVAANLPTP FLAEAMHYAA NRQSFNSAID THQYVQKRLV DIAIGMERNR
Burkholderia cepacia               GLVAAQVTAP YLRDAIAYCR DRRSFDSTID EHQYVQRRLV DLQIGIERGT
Burkholderia plantarii             GLVSAAIAEP YVEEALDYVA ARTSFGEPVA AHQYVQKRVV DARIGIERTR
Burkholderia plantarii IVD         AGGPTGIMAA CLDAVVPYIH DRKQFGQSIG EFQLIQGKVA DMYTNFQACR
Burkholderia plantarii SCAD        AAQAVGIARA AFDKARRYAA ERTQFGKPIA EHQAIAEKLA DMATELNAAR
Rattus norvegicus IVD              AGGPLGIMQA VLDHTIPYLH VREAFGQKIG QFQLMQGKMA DMYTRLMACR
Rattus norvegicus SCAD             ASQALGIAQA SLDCAVKYAE NRHAFGAPLT KLQNIQFKLA DMALALESAR

                                   295
Phaeobacter inhibens               WVSYGALHQL LSGAPEAAMT ----CSISKL VGAETIVEGA MDLMKLYGSK
Phaeobacter inhibens IVD           SYVYEVAKAC DKG-----TV TRQDAAACCL YASEVAMTQA HQAVQAFGGA
Phaeobacter inhibens SCAD          CMIEETARRK DAGQNVSI-- ---DAACCKM YASEMVGRVA DRAVQILGGA
Pseudovibrio sp. JE062             WTAYGALQQL LAGAPEAGMT ----CSIAKL VGAEAITSGA IDLLKLYGTK
Epibacterium mobile                WVSYGALHQL LSGAPEAVMS ----CSIAKL VGASAITDGA VDLLKLYGSR
Ruegeria sp. P4                    WVSYGALHQL LSGAPEAVMS ----CSIAKL AGASAITDGA VDLLKLYGSR
Paracoccus denitrificans           WTAYGALHQL LGGAPEAAMS ----CSIAKI AGADTVIAAA VDLLRLHGSD
Paraburkholderia phenazinium       WLAYAALDRL LGKQPDSLML ----CSIAKL VGADDLVNSA ISLMKLQGST
Pseudoduganella violaceinigra      WLAYGALTQL MAGDREALLT ----CSAAKL VGAEDLINGA ISLVKLYGSL
Pseudomonas putida                 WMVYAALDQL LGNDPQALMS ----CSIAKL GAAQDFIHSA ISLLKLYGSL
Burkholderia cepacia               WLARGALAQL LTDHPQALMT ----CSIAKL VGAQDLVDGA LGLVRLYGSL
Burkholderia plantarii             WLAYAALDQL LNRRDGSVML ----CSIAKL VGANDLIDTA ISLVRLRGST
Burkholderia plantarii IVD         AYLYAVGRHL DAAGSQHVRQ ARKDCAGVIL YTAERATWMA GEAIQILGGN
Burkholderia plantarii SCAD        LLVHHAARLR TAG-----LP CLSEASQAKL FASEMSERVC TAAIQIHGGY
Rattus norvegicus IVD              QYVYNVARAC DEG-----HI TAKDCAGVIL YTAECATQVA LDGIQCLGGN
Rattus norvegicus SCAD             LLTWRAAMLK DNK-----KP FTKESAMAKL AASEAATAIS HQAIQILGGM
```

```
                                   341                      361
Phaeobacter inhibens               GY-HEGQITT FLRDALAFCS VGGTEEMHRR NIFGQMARLH AK-AKQAADP
Phaeobacter inhibens IVD           GYLSDNPVGR IFRDAKLMEI GAGTSEIRRM LIGRELMSQM ----------
Phaeobacter inhibens SCAD          GYIADYGVER FFRDVRLFRI YEGTTQIQQL VIARGMIRQA SA--------
Pseudovibrio sp. JE062             GY-HQGEVST FLRDALAFCA VGGTEEMHRR NIISQMKRLE AKAAKKAAEP
Epibacterium mobile                GY-HEGEVST FLRDALAFCS VGGTEEMHRR NIMNQMMRKA RPPAKAAVEP
Ruegeria sp. P4                    GY-HEGEVST FLRDALAFCS VGGTEEMHRR NIMNQMMRKA RPAAKAAVER
Paracoccus denitrificans           GY-HEGRVSG FLRDAMAFAS VGGTDEMHRR NIMGQMLRLH RRAQVAPVAA
Paraburkholderia phenazinium       GY-HEGVLSV LARDALGFAS VGGTEEMHRK NIFNQMVRLR ERSQQVQEAA
Pseudoduganella violaceinigra      GY-HNNDIST LARNALGFAS VGGTEEMHRK NIYNQLVRLS K---------
Pseudomonas putida                 GY-HEGDIAT LVKDALGFAS VGGTEEMHQK NIFNQLLRLT QAG-------
Burkholderia cepacia               GY-QAGPVAA FASDALGFMS VGGTEEMHRK NIFNQMMRAG ----------
Burkholderia plantarii             GY-HEGELAV LARDALGFAS VGGTEEMHRK NIFNQMQRLH ARANGAARA
Burkholderia plantarii IVD         GYINEYPVGR LWRDAKLYEI GAGTSEIRRM LIGRELFAET A---------
Burkholderia plantarii SCAD        GYLADYEVER HYRDARITQI YEGTSEVQRM VIARQLLA-- ----------
Rattus norvegicus IVD              GYINDFPMGR FLRDAKLYEI GGGTSEVRRL VIGRAFNADF R---------
Rattus norvegicus SCAD             GYVTEMPAER YYRDARITEI YEGTSEIQRL VIAGHLLRSY RS--------

                                   389
Phaeobacter inhibens               ATEKTLEPA
Phaeobacter inhibens IVD           ---------
Phaeobacter inhibens SCAD          ---------
Pseudovibrio sp. JE062             PQERILETA
Epibacterium mobile                ARD--LETA
Ruegeria sp. P4                    ARD--LETA
Paracoccus denitrificans           QQMRVAG--
Paraburkholderia phenazinium       A--------
Pseudoduganella violaceinigra      ---------
Pseudomonas putida                 ---------
Burkholderia cepacia               ---------
Burkholderia plantarii             ---------
Burkholderia plantarii IVD         ---------
Burkholderia plantarii SCAD        ---------
Rattus norvegicus IVD              ---------
Rattus norvegicus SCAD             ---------
```

Figure S62: Full structure-based multiple sequence alignment showing TdaE-homologs from different bacterial species in comparison with classical acyl-CoA dehydrogenases from *P. inhibens*, *B. plantarii* as well as *R. norvegicus*. The structure-based sequence alignment was generated using the online tool T-coffee[5] and visualized in SeaView[6]. Final color editing to highlight important catalytic residues was carried out in PowerPoint.

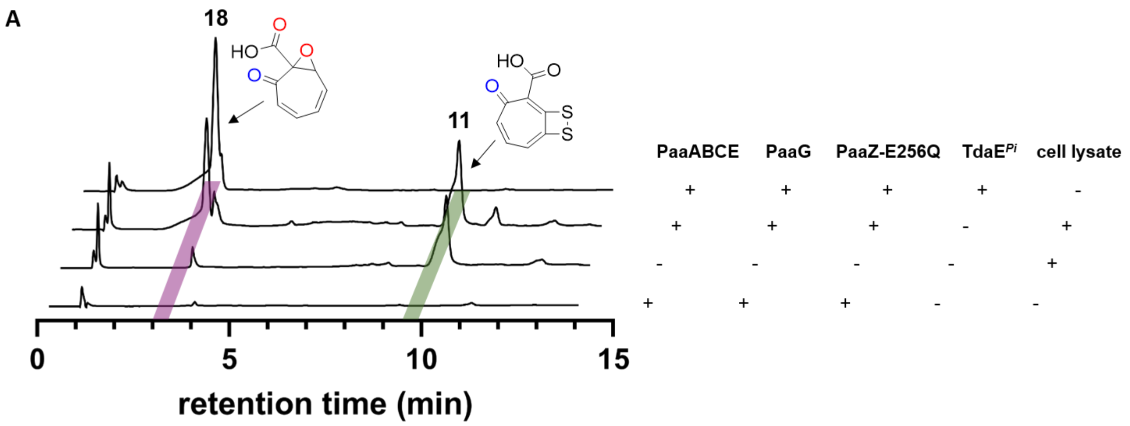

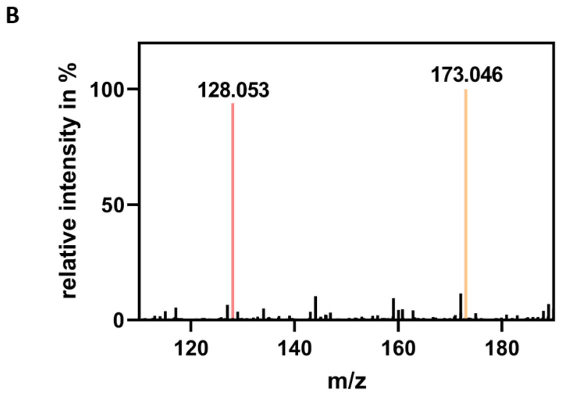

Figure S63: Conversion of compound 4 **into compound** 18 **by the cell-free lysate of *P. inhibens*. A,** RP-HPLC chromatograms at 300 nm. The *purple* line indicates compound **18**, and the *green* line highlights compound **11,** which can be detected in the cell-free lysate of *P. inhibens* after organic extraction with EtOAc. Samples were incubated at 30 °C and 900 rpm for 10 min to obtain compound **4**, before addition of cell-free lysate to the enzyme assay and incubation for another 2 min under the same conditions. The results indicate the rapid production of compound **18** from compound **4** in the presence of cell-free lysate. **B,** LC-HRMS analysis of **($^{13}C_8$)-18** formation in the assay of **($^{13}C_8$)-4** with *P. inhibens* cell-free lysate.

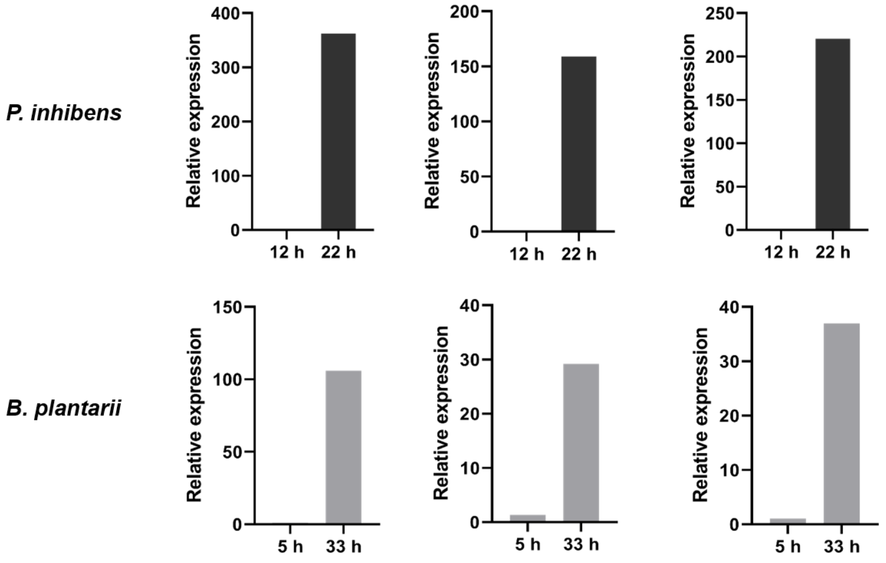

Figure S64: RT-qPCR analysis of mRNA expression levels of *tdaE* at different cultivation points in *Phaeobacter inhibens* and *Burkholderia plantarii*. Total RNA was extracted from cells harvested at compound non-production (12 h for **11** and 5 h for **9**) and production (22 h for **11** and 33 h for **9**) stages. Three biological replicates were performed and are displayed independently (*left*, *middle* and *right*). Note that no standard error was calculated from the three replicates, because the upregulation factor in the first biological replicate (*left*) varies significantly from the second and the third (*middle* and *right*) due to fresher RNA and cDNA used as template for RT-qPCR in the left replicate.

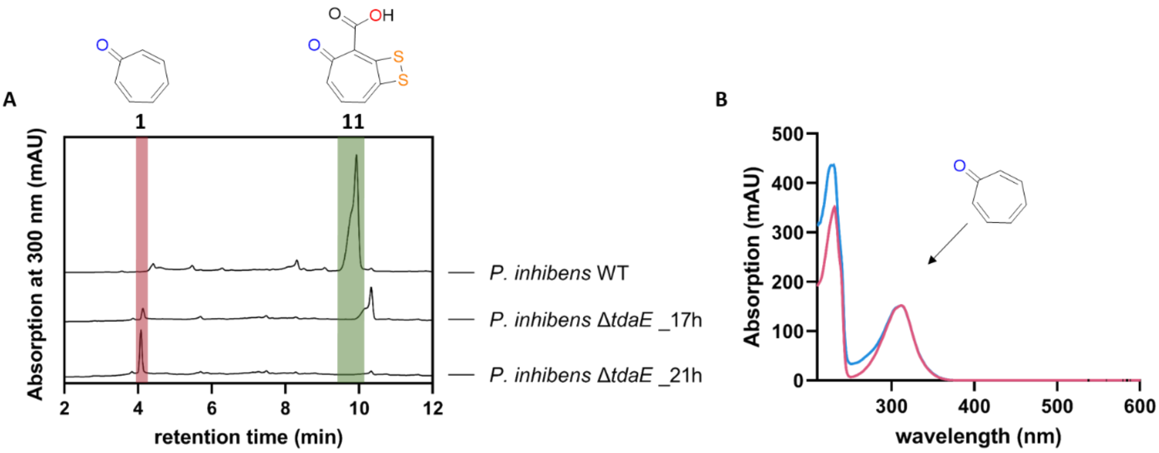

Figure S65: Analysis of compound 11 **production by a wild type and a Δ*tdaE* mutant strain of *P. inhibens*. A,** RP-HPLC analysis at 300 nm showing that compound **11** is produced by the wild type strain, but not by the Δ*tdaE* mutant. Note that the shoulder (~9.9 min) observed in the mutant strain after 17 h of cultivation does not correspond to compound **11** (a major difference in their UV-visible absorption spectra is observed). In contrast, compound **1** was produced by the mutant and its production increased over time (17 *vs* 21 h). **B,** Overlay of the UV-visible absorption spectra of compound **1** standard (*pink*) and the compound (panel A, highlighted in red) accumulating in the Δ*tdaE* mutant (*blue*), indicating that **1** is the main metabolite formed in the Δ*tdaE* mutant.

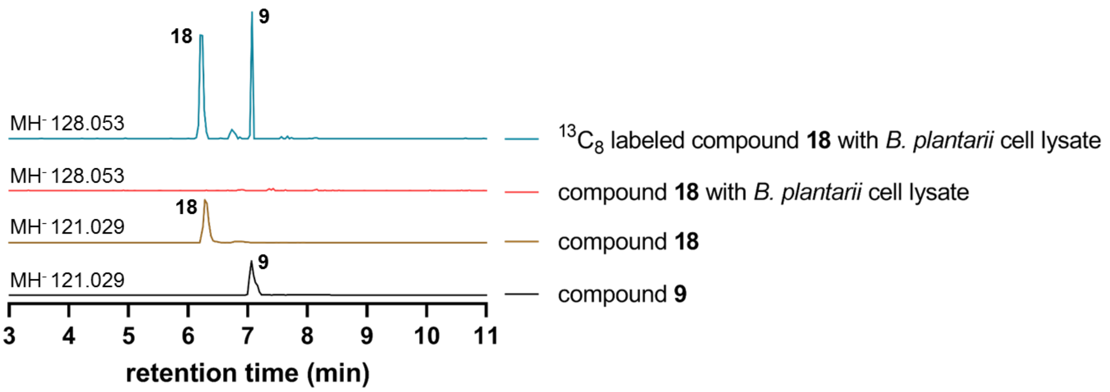

Figure S66: Conversion of compound 18 **into compound** 9 **by *B. plantarii* cell-free lysate.** $^{13}C_8$-**18** or alternatively unlabeled **18** were incubated with cell-free lysates of *B. plantarii* at 30 °C and 900 rpm for 5 min prior to extraction with EtOAC + 1 % FA. Then, the samples were concentrated in the speedvac and analyzed by LC-HRMS. For the assay started with $^{13}C_8$-**18**, the extracted ion chromatogram (EIC) trace for MH^-, 128.053 (*cyan* line) confirms the formation of $^{13}C_7$-**9** at 7.1 min while the peak at 6.2 min results from the in-source decarboxylation of residual $^{13}C_8$-**18** (note that the spontaneous formation of compound **9** from **18** in assays without *B. plantarii* lysate is much slower, see Fig. S14). As expected, these peaks do not show up in the EIC trace for MH^-, 128.053 of the control assay (*pink* line) started with unlabeled **18**. The bottom traces show standards for **18** (*brown* line) and **9** (*black* line).

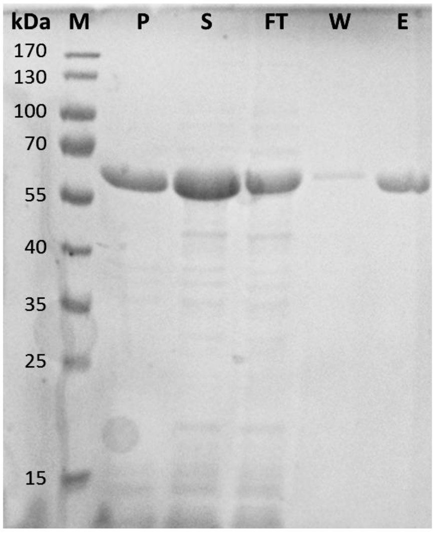

Figure S67: SDS-PAGE analysis of the different fractions collected in the course of the affinity purification of the putative decarboxylase from *B. plantarii*. In lane 1 the marker (**M**; PageRuler Prestained protein ladder, Thermo Scientific), in lane 2 the cell pellet after lysis (**P**), in lane 3 the cleared cell lysate (**S**), in lane 4 the column flow through (**FT**), in lane 5 the wash fraction (**W**) and in lane 6 the elution fraction (**E**) are shown.

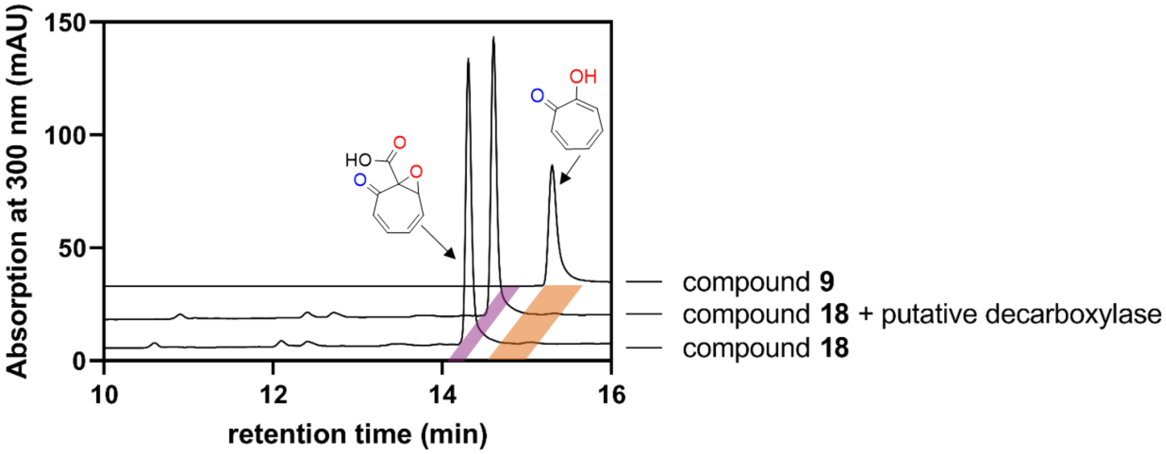

Figure S68: Incubation of compound 18 **with a putative decarboxylase from *B. plantarii* encoded in the "*tda* gene cluster".** Compound **18** was incubated with the putative decarboxylase (10 µM) in 50 mM Tris-HCl pH 8 at 30 °C and 900 rpm for 20 min. Reactions were quenched and extracted with EtOAc + 1 % FA and analyzed by HPLC-DAD. The obtained results indicate that the putative decarboxylase does not catalyze the formation of compound **9** from compound **18**.

References

(1) Teufel, R.; Mascaraque, V.; Ismail, W.; Voss, M.; Perera, J.; Eisenreich, W.; Haehnel, W.; Fuchs, G. Bacterial phenylalanine and phenylacetate catabolic pathway revealed. *Proc. Natl. Acad. Sci. U S A* **2010**, *107*, 14390–14395.

(2) Teufel, R.; Gantert, C.; Voss, M.; Eisenreich, W.; Haehnel, W.; Fuchs, G. Studies on the mechanism of ring hydrolysis in phenylacetate degradation: a metabolic branching point. *J. Biol. Chem.* **2011**, *286*, 11021–11034.

(3) Matthews, A.; Saleem-Batcha, R.; Sanders, J. N.; Stull, F.; Houk, K. N.; Teufel, R. Aminoperoxide adducts expand the catalytic repertoire of flavin monooxygenases. *Nat. Chem. Biol.* **2020**, *16*, 556–563.

(4) Erb, T. J.; Ismail, W.; Fuchs, G. Phenylacetate metabolism in thermophiles: characterization of phenylacetate-CoA ligase, the initial enzyme of the hybrid pathway in Thermus thermophilus. *Curr. Microbiol.* **2008**, *57*, 27–32.

(5) Armougom, F.; Moretti, S.; Poirot, O.; Audic, S.; Dumas, P.; Schaeli, B.; Keduas, V.; Notredame, C. Expresso: automatic incorporation of structural information in multiple sequence alignments using 3D-Coffee. *Nucleic Acids Res.* **2006**, *34*, W604-8.

(6) Gouy, M.; Guindon, S.; Gascuel, O. SeaView version 4: A multiplatform graphical user interface for sequence alignment and phylogenetic tree building. *Mol. Biol. Evol.* **2010**, *27*, 221–224.

(7) Waterhouse, A.; Bertoni, M.; Bienert, S.; Studer, G.; Tauriello, G.; Gumienny, R.; Heer, F. T.; Beer, T. A. P. de; Rempfer, C.; Bordoli, L.; Lepore, R.; Schwede, T. SWISS-MODEL: homology modelling of protein structures and complexes. *Nucleic Acids Res.* **2018**, *46*, W296-W303.

(8) Schachter, D.; Taggart, J. V. Benzoyl coenzyme A and hippurate synthesis. *J. Biol. Chem.* **1953**, *203*, 925–934.

(9) Miwa, S.; Kihira, E.; Yoshioka, A.; Nakasone, K.; Okamoto, S.; Hatano, M.; Igarashi, M.; Eguchi, Y.; Kato, A.; Ichikawa, N.; Sekine, M.; Fujita, N.; Kanesaki, Y.; Yoshikawa, H.; Utsumi, R. Identification of the Three Genes Involved in Controlling Production of a Phytotoxin Tropolone in Burkholderia plantarii. *J. Bacteriol.* **2016**, *198*, 1604–1609.

(10) Gottlieb, H. E.; Kotlyar, V.; Nudelman, A. NMR Chemical Shifts of Common Laboratory Solvents as Trace Impurities. *J. Org. Chem.* **1997**, *62*, 7512–7515.

(11) Wu, G.; Deng, Y.; Wu, C.; Zhang, Y.; Wang, J. Synthesis of α-aryl esters and nitriles: deaminative coupling of α-aminoesters and α-aminoacetonitriles with arylboronic acids. *Angew. Chem.* **2014**, *53*, 10510–10514.

(12) Smith, A. B.; Dorsey, B. D.; Ohba, M.; Lupo, A. T.; Malamas, M. S. Preparation, reactivity, and spectral properties of 1,3-dioxin vinylogous esters: versatile .beta.-ketovinyl cation equivalents. *J. Org. Chem.* **1988**, *53*, 4314–4325.

(13) Ikeda, S.; Shibuya, M.; Kanoh, N.; Iwabuchi, Y. Synthetic studies on daphnicyclidin A: enantiocontrolled construction of the BCD ring system. *Org. Lett.* **2009**, *11*, 1833–1836.

(14) García Ruano, J. L.; Fajardo, C.; Fraile, A.; Martín, M. R. m-CPBA/KOH: an efficient reagent for nucleophilic epoxidation of gem-deactivated olefins. *J. Org. Chem.* **2005**, *70*, 4300–4306.

(15) Hojo, M.; Ueda, T.; Inoue, T.; Ike, M.; Kobayashi, M.; Nakai, H. UV-visible and 1H or 13C NMR spectroscopic studies on the specific interaction between lithium ions and the anion from tropolone

or 4-isopropyltropolone (hinokitiol) and on the formation of protonated tropolones in acetonitrile or other solvents. *J. Phys. Chem. B* **2007**, *111*, 1759–1768.

(16) Lyons, D. J. M.; Empel, C.; Pace, D. P.; Dinh, A. H.; Mai, B. K.; Koenigs, R. M.; Nguyen, T. V. Tropolonate Salts as Acyl-Transfer Catalysts under Thermal and Photochemical Conditions: Reaction Scope and Mechanistic Insights. *ACS Catal.* **2020**, *10*, 12596–12606.
